# Role of methanesulfonic acid in atmospheric particle nucleation and growth

**DOI:** 10.1038/s41586-026-10810-2

**Published:** 2026-06-24

**Authors:** Rima Baalbaki, Jiali Shen, Mario Simon, Hannah Klebach, Samuel Ruhl, Jenna DeVivo, Mingyi Wang, Wiebke Scholz, Lubna Dada, Birte Rörup, Dominik Stolzenburg, Hanna E. Manninen, Eva Sommer, Lucía Caudillo-Plath, Guillaume Marie, Martin Friedrich, Wenjuan Yu, Markus Leiminger, Dina Alfaouri, Antonio Amorim, Tatjana Arnoldi-Meadows, Hannah Beckmann, Moritz Berntheusel, Steffen Bräkling, Zoé Brasseur, Randall Chiu, Jonathan Duplissy, Henning Finkenzeller, Martin Heinritzi, Felix Kunkler, Houssni Lamkaddam, Brandon Lopez, Naser Mahfouz, Vladimir Makhmutov, Monica Martinez, Ruby Marten, Dario Massabo, Roy Mauldin, Bernhard Mentler, Markus Müller, Maxim Philippov, Ana A. Piedehierro, Pedro Rato, Tobias Reinecke, Sarah Richter, Douglas M. Russell, Benjamin Schulze, Mihnea Surdu, Roseline C. Thakur, Yee Jun Tham, Ping Tian, António Tomé, Yandong Tong, Jens Top, Andrea C. Wagner, Dongyu S. Wang, Yonghong Wang, Ryan X. Ward, Stefan K. Weber, André Welti, Yusheng Wu, Marcel Zauner-Wieczorek, Jiangyi Zhang, Joachim Curtius, Neil M. Donahue, Imad El Haddad, Richard C. Flagan, Armin Hansel, Hartwig Harder, Andreas Kürten, Tuukka Petäjä, Siegfried Schobesberger, Mikko Sipilä, Rainer Volkamer, Paul M. Winkler, Douglas R. Worsnop, Theodoros Christoudias, Andrea Pozzer, Markku Kulmala, Jasper Kirkby, Katrianne Lehtipalo, Xu-Cheng He

**Affiliations:** 1 Institute for Atmospheric and Earth System Research/Physics, University of Helsinki, Helsinki, Finland; 2 Climate and Atmosphere Research Center (CARE-C), The Cyprus Institute, Nicosia, Cyprus; 3 Helsinki Institute of Physics, University of Helsinki, Helsinki, Finland; 4 Institute for Atmospheric and Environmental Sciences, Goethe University Frankfurt, Frankfurt am Main, Germany; 5 Atmospheric Chemistry Department, Max Planck Institute for Chemistry, Mainz, Germany; 6 Center for Atmospheric Particle Studies, Carnegie Mellon University, Pittsburgh, PA USA; 7 Department of Chemistry, Carnegie Mellon University, Pittsburgh, PA USA; 8 Department of the Geophysical Sciences, The University of Chicago, Chicago, IL USA; 9 Institute for Ion Physics and Applied Physics, University of Innsbruck, Innsbruck, Austria; 10 Center for Energy and Environmental Sciences, Paul Scherrer Institute, Villigen, Switzerland; 11 Institute of Materials Chemistry, TU Wien, Vienna, Austria; 12 Faculty of Physics, University of Vienna, Vienna, Austria; 13 CERN, European Organization for Nuclear Research, Geneva, Switzerland; 14 IONICON Analytik GmbH, Innsbruck, Austria; 15 Faculty of Sciences, University of Lisbon, Lisbon, Portugal; 16 TOFWERK, Thun, Switzerland; 17 Department of Chemistry and Cooperative Institute for Research in the Environmental Sciences, University of Colorado Boulder, Boulder, CO USA; 18 Lebedev Physical Institute, Russian Academy of Sciences, Moscow, Russian Federation; 19 Moscow Institute of Physics and Technology (National Research University), Moscow, Russian Federation; 20 Department of Physics, University of Genoa, Genoa, Italy; 21 National Institute of Nuclear Physics, INFN-Genoa, Genoa, Italy; 22 Department of Atmospheric and Oceanic Sciences, University of Colorado, Boulder, CO USA; 23 Finnish Meteorological Institute, Helsinki, Finland; 24 Division of Chemistry and Chemical Engineering, California Institute of Technology, Pasadena, CA USA; 25 School of Marine Sciences, State Key Laboratory of Environmental Adaptability for Industrial Products, Sun Yat-sen University, Zhuhai, China; 26 Beijing Weather Modification Center, Beijing, China; 27 Instituto Dom Luiz (IDL), University of Beira Interior, Covilhã, Portugal; 28 Aerosol Physics Laboratory, Physics Unit, Faculty of Engineering and Natural Sciences, University of Tampere, Tampere, Finland; 29 Laboratory of Atmospheric Environment and Pollution Control, Research Center for Eco-Environmental Sciences, Chinese Academy of Sciences, Beijing, China; 30 Department of Chemical Engineering, Carnegie Mellon University, Pittsburgh, PA USA; 31 Department of Technical Physics, University of Eastern Finland, Kuopio, Finland; 32 Aerodyne Research Inc., Billerica, MA USA; 33 Joint International Research Laboratory of Atmospheric and Earth System Sciences, School of Atmospheric Sciences, Nanjing University, Nanjing, China; 34 Aerosol and Haze Laboratory, Beijing Advanced Innovation Center for Soft Matter Science and Engineering, Beijing University of Chemical Technology, Beijing, China

**Keywords:** Atmospheric science, Climate change

## Abstract

Dimethyl sulfide (DMS; CH_3_SCH_3_) from marine phytoplankton is a notable source of atmospheric sulfur^1^. Its oxidation products include sulfuric acid (SA; H_2_SO_4_) and methanesulfonic acid (MSA; CH_3_SO_3_H), which has a higher yield than SA below 10 °C (ref. ^2^). Although SA is known to drive the formation of new particles^3^, which may subsequently grow and act as cloud condensation nuclei (CCN), the role of MSA remains unclear^4^. Here, in experiments performed under atmospheric conditions at the CERN CLOUD (Cosmics Leaving OUtdoor Droplets) chamber, we show that MSA nucleates together with ammonia (NH_3_) below −10 °C, at rates comparable with SA-NH_3_. Moreover, MSA and SA nucleate synergistically below −10 °C, forming multi-acid molecular clusters with NH_3_. Even at ultralow NH_3_ levels, MSA drives particle growth at or near the kinetic limit below 9 °C and above 40% relative humidity (RH). Because MSA and SA generally coexist at similar concentrations in cool marine regions, our findings indicate that nucleation rates may be accelerated up to tenfold and growth rates up to twofold compared with SA-NH3 alone. Our global model simulations indicate that MSA can enhance CCN concentrations, especially in polar regions. We propose that MSA might be an important driver of biogenic particles in cool, pristine marine regions of both the present-day and pre-industrial atmospheres and yet is unaccounted for in global climate models^5^.

## Abstract (web summary)

Experiments carried out at atmospheric conditions show that methanesulfonic acid is an important driver of particle formation and growth in cold marine environments, with simulations indicating enhanced cloud condensation nuclei concentrations, especially in polar regions.

## Main

The importance of DMS from phytoplankton for marine aerosols and clouds has long been recognized. Pioneering work^6^ in 1978 established the first association between DMS, at pungent concentrations near the Great Barrier Reef, and elevated particle number concentrations. Subsequent observations in marine environments have consistently found an association between DMS and particle formation^7–10^. This connection underpinned the CLAW hypothesis, which proposed a biogenic feedback loop for climate regulation linking marine DMS emissions, particle formation and cloud brightening^11^. Although DMS emissions are estimated to contribute to radiative cooling^12^, the magnitude of their effect remains uncertain^13^ owing to incomplete understandings of emission sources^14^, chemical processes^15,16^ and the role of DMS oxidation products in particle nucleation and growth^5,17^.

New particle formation occurs when low-volatility vapours reach sufficient supersaturation to condense and form stable molecular clusters around 1–2 nm in size. Through a combination of condensation and coagulation, these clusters may grow to sizes above about 50 nm, at which they can act as CCN. Two DMS oxidation products that influence new particle formation are SA and MSA, which are widely found in the marine boundary layer at concentrations of 10^4^–10^7^ cm^−3^. Although MSA originates mainly from DMS oxidation and, to a lesser extent, from methanethiol (CH_3_SH)^18^, SA has further sources, such as volcanic sulfur dioxide (SO_2_), hydrogen sulfide (H_2_S)^19^ and anthropogenic SO_2_. Nevertheless, in remote marine regions, MSA concentrations are comparable with SA concentrations and may even exceed them in polar regions^20–25^ and at high altitudes^26,27^.

Whereas the importance of SA for atmospheric particle nucleation and growth is well established^3,28^, the role of MSA remains inconclusive. Numerous ambient studies have shown that MSA contributes to particle growth and mass^21,29–34^. However, the contribution of MSA to particle nucleation under atmospheric conditions remains uncertain. Theoretical studies suggest that, although MSA is a weaker acid than SA, it can facilitate nucleation by clustering with SA and base vapours^35,36^. Experimentally, MSA has been shown to form particles with amines and ammonia (NH_3_) at high (ppbv) precursor concentrations and warm temperatures, with water playing an important role in the process^37–39^. More recent flow tube experiments measured SA-MSA and MSA-MSA dimers at 25 °C and 20% RH with 2 × 10^8^ cm^−3^ MSA, (2–8) × 10^8^ cm^−3^ SA and base vapours (ammonia and amines)^4^. However, only relatively small (20–30%) increases (or even decreases) in particle counts were measured when injecting 2.5 × 10^8^ cm^−3^ MSA into SA-base vapour mixtures^4^. In summary, ambient observations show that MSA contributes substantially to particle mass and laboratory studies indicate that high RH promotes MSA particle formation at warm temperatures in the presence of base vapours and acid concentrations exceeding typical atmospheric levels. However, MSA remains unrepresented in global climate models because of the lack of laboratory measurements of its contributions to particle nucleation and growth rates under atmospheric conditions.

Here we report experiments performed at the CERN CLOUD chamber^3^ to study the role of MSA in new particle formation under atmospheric conditions between −52 °C and +9 °C. For most experiments, MSA was produced in the chamber from oxidation of DMS with hydroxyl (OH) radicals produced by photolysis of ozone. However, for several experiments at +9 °C, MSA was directly injected into the chamber from a temperature-controlled evaporator. SA was produced from oxidation of both DMS and SO_2_, separately injected into the chamber, to allow independent control of the SA and MSA concentrations. Experiments were carried out with NH_3_ between contaminant level (<1 pptv to 4 pptv, depending on temperature) and 760 pptv. Some experiments also involved photolysis of molecular iodine to produce iodine oxoacids (IA_*x*_), which we define here as the sum of iodic acid (IA; HIO_3_) and iodous acid (IOA; HIO_2_)^40^. Further details of the CLOUD facility and its analysing instruments are provided in Methods.

## Particle nucleation rates

**Fig. 1:**
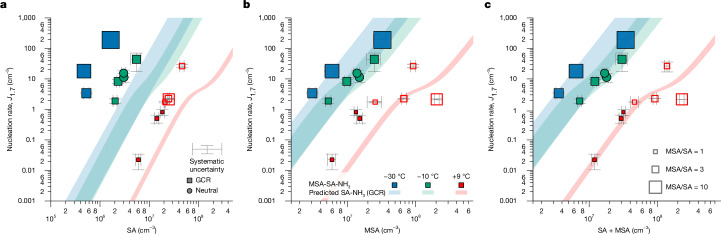
MSA-SA-NH_3_ particle nucleation rates, *J*_1.7_, versus acid concentrations. **a**–**c**, Particle nucleation rates at 1.7 nm (*J*_1.7_) under neutral (circles) and GCR (squares) conditions versus SA (**a**), MSA (**b**) and summed acid (SA + MSA) (**c**) concentrations at −30 °C (blue), −10 °C (green) and +9 °C (red). The filled symbols indicate experiments in which SA and MSA were generated by DMS oxidation. The open red squares indicate experiments at +9 °C in which SA was produced by SO_2_ oxidation and MSA was injected directly from an evaporator. The coloured bands indicate the expected SA-NH_3_ nucleation rates under GCR conditions according to Dunne et al.^28^. The bands are identical across all panels (with the *x*-axis interpreted as SA) and are colour-coded by temperature. The band widths reflect the range of NH_3_ concentration for the filled symbols at each temperature: 46–111 pptv at +9 °C, 139–460 pptv at −10 °C and 4–121 pptv at −30 °C. Ammonia was not measured during the −30 °C experiments but instead estimated using a box model (Methods). The experimental conditions for the filled symbols were 5–280 ppbv O_3_, 0–0.5 ppbv SO_2_, 0.5–2.5 ppbv DMS and 40–80% RH. For the open red squares, the experimental conditions were 38–44 ppbv O_3_, 5.8–6.2 ppbv SO_2_, <4 pptv NH_3_, <1 pptv DMA and 62% RH. Here, and throughout this study, measurements labelled +9 °C correspond to a temperature range of 9–10 °C. Each data point represents the mean values of the steady-state measurement period of a single experiment. Vertical and horizontal error bars show ±1*σ* measurement uncertainty of *J*_1.7_ and acid concentrations, respectively. The systematic scale uncertainties for SA and MSA concentrations (50%, 200%) and *J*_1.7_ (±30%) are shown separately in panel **a**.

In Fig. 1, we compare our measured particle nucleation rates at 1.7 nm (*J*_1.7_) for MSA-SA-NH_3_ (data points) at −30 °C, −10 °C and +9 °C with those expected for SA-NH_3_ (coloured bands), based on previous measurements at CLOUD^28^. Water is implicit in all of the chemical systems described here. The nucleation rates versus SA, MSA and summed acids (SA + MSA) are shown in Fig. 1a–c, respectively. Experiments in which SA and MSA were both generated from DMS are indicated by filled symbols. To extend the MSA:SA ratio range at +9 °C, some experiments were also carried out with direct injection of MSA vapour and with SA produced by oxidation of SO_2_ (indicated by hollow red squares). The latter experiments include contaminant NH_3_ (<4 pptv) and contaminant dimethylamine (DMA; <1 pptv), estimated from an atmospheric pressure interface time-of-flight (APi-TOF) mass spectrometer. Despite such low base vapour concentrations, they were sufficient to reach nucleation rates comparable with those for SA-NH_3_ with 46–111 pptv NH_3_ at +9 °C (as seen by the alignment of the filled and hollow red squares in Fig. 1a). All but two measurements (indicated by circle symbols in Fig. 1) were made under galactic cosmic ray (GCR) conditions, so these data are insufficient to distinguish ion-induced effects at low nucleation rates.

For all temperatures, nucleation rates in the presence of MSA exceed those expected from SA-NH_3_ alone. The nucleation rates of the filled squares are around 100 times faster than those expected from SA-NH_3_ at −30 °C and ten times faster at −10 °C and +9 °C (Fig. 1a). This enhancement reflects the higher MSA:SA ratio at −30 °C (5.8–20) compared with −10 °C (2.6–4.9) and +9 °C (0.7–1.2), owing to increased MSA production at lower temperatures^2^. Figure 1a,b shows that the nucleation rates at −30 °C and −10 °C depend on MSA as well as SA and they align with those expected for SA-NH_3_ when MSA is considered equivalent to SA (Fig. 1c). However, the behaviour is different at +9 °C; here the three hollow points at fixed 2 × 10^7 ^cm^−3^ SA show no change of nucleation rate after increasing the MSA concentration tenfold from 2.6 × 10^7^ cm^−3^ to 2 × 10^8^ cm^−3^ (Fig. 1b). By contrast, a doubling of the SA concentration under the same base vapour conditions increases the nucleation rate by one order of magnitude (Fig. 1a). The discrepancy between the measured and predicted SA-NH_3_ nucleation rates at +9 °C (Fig. 1a) is within the combined systematic uncertainties of the measurements and parameterization. We conclude that MSA at these concentrations is contributing to nucleation on an equal basis as SA below −10 °C but is not contributing to nucleation above +9 °C.

**Extended Data Fig. 1:**
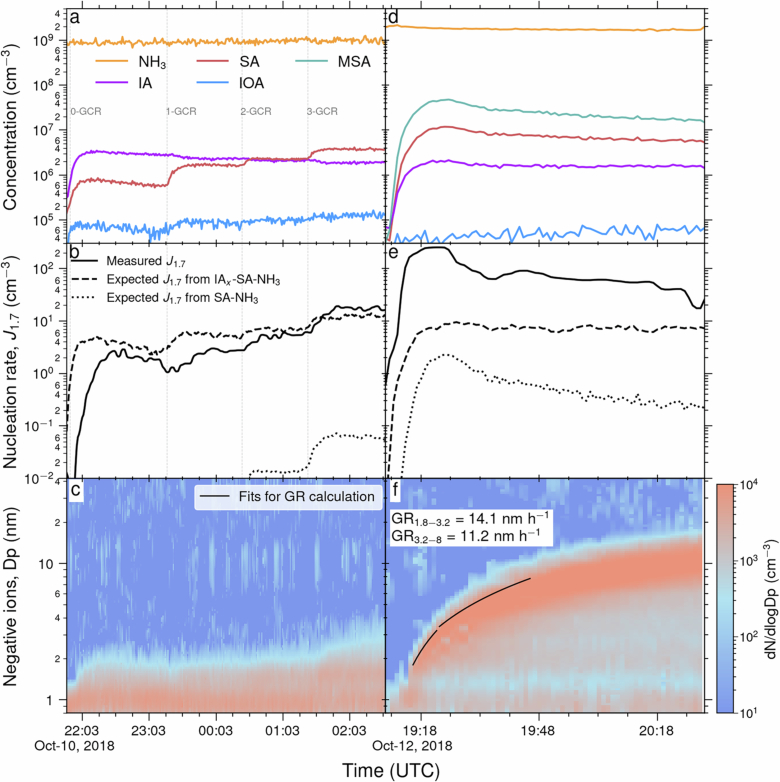
New particle formation from IA_*x*_-SA-NH_3_(-MSA) at −10 °C. The upper panels show the evolution of vapour concentrations, the middle panels show the particle nucleation rates at 1.7 nm, *J*_1.7_, and the lower panels show the size distributions (d*N*/dlog*D*_p_) of negatively charged particles of mobility diameter between 0.8 and 42.0 nm. MSA was absent for the left-hand panels (**a**–**c**) and present for the right-hand panels (**d**–**f**). The vertical dashed lines in panels **a** and **b** represent new stages in which SA was increased. The curves in panels **b** and **e** show the measured *J*_1.7_ (solid curve) and those expected for SA-NH_3_ (dotted curve) and IA_*x*_-SA-NH_3_ (dashed curve). The experimental conditions for panels **a**–**c** are 41 ppbv O_3_, 1.6 ppbv SO_2_ and 72% RH. MSA was generated in the right-hand experiments through the oxidation of DMS. The experimental conditions for panels **d**–**f** are 161 pptv DMS, 4.7 ppbv O_3_, 1.6 ppbv SO_2_ and 67% RH. The strong enhancement of particle nucleation and growth rates after addition of around 3 × 10^7^ cm^−3^ MSA demonstrates that MSA participates in multi-acid MSA-IA_*x*_-SA-NH_3_ particle formation.

We also performed experiments to investigate the interaction of MSA with IA_*x*_-SA-NH_3_, as iodine oxoacids are important for new particle formation in marine and polar atmospheres^40,41^. In Extended Data Fig. 1 (left-hand panels), we show an experiment at −10 °C with fixed IA_*x*_ (1.9–3.6 × 10^6^ cm^−3^) and NH_3_ (0.9 × 10^9^ cm^−3^, equivalent to 35 pptv), with SA increased in four steps^40^ from 5 × 10^5^ to 4.1 × 10^6^ cm^−3^. The nucleation rate, *J*_1.7_, increases from about 1 to 20 cm^−3^ s^−1^, as shown in our earlier study^40^. In the right-hand panels, this experiment is repeated with similar IA_*x*_ (1.7 × 10^6^ cm^−3^), SA (7.5 × 10^6^ cm^−3^) and NH_3_ (1.8 × 10^9^ cm^−3^, equivalent to 69 pptv) but now including MSA (about 3 × 10^7^ cm^−3^). Under these conditions, the nucleation rate increases by more than one order of magnitude, reaching about 200 cm^−3^ s^−1^. The nucleation rates expected from the parameterization of the IA_*x*_-SA-NH_3_ and SA-NH_3_ systems are around one-tenth of the measured values (Extended Data Fig. 1e), whereas the nucleation rates expected for the experiment without MSA agree with the measured values (Extended Data Fig. 1b). These experiments demonstrate that MSA participates in multi-acid MSA-IA_*x*_-SA-NH_3_ nucleation at −10 °C and below. It implies that all three acid vapours (SA, IA_*x*_ and MSA) need to be considered together when assessing marine new particle formation.

**Extended Data Fig. 2:**
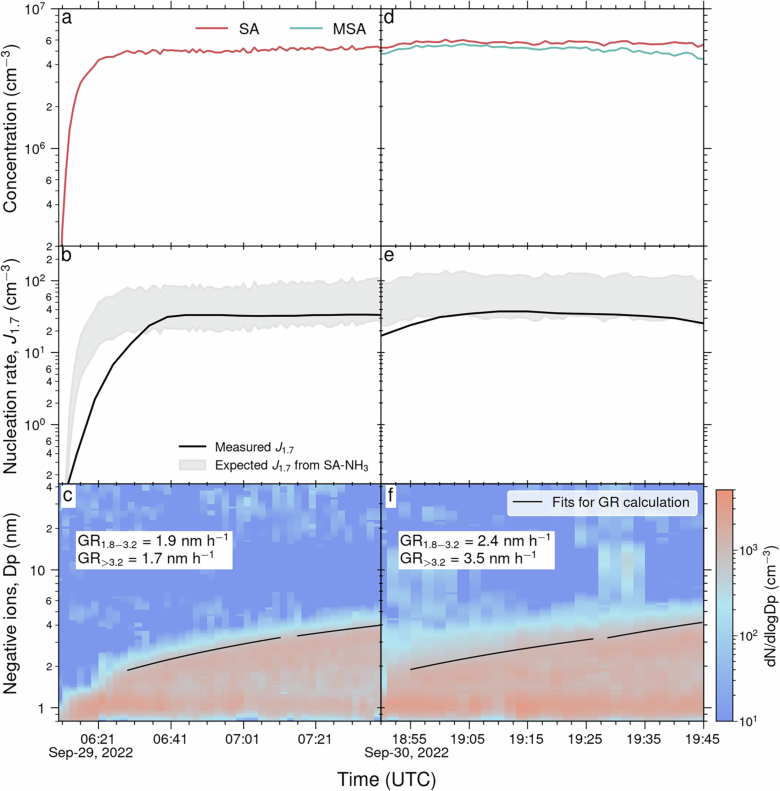
Effect of MSA on nucleation and growth at −52 °C, in the absence of NH_3_. The upper panels show the evolution of vapour concentrations, the middle panels show the particle nucleation rates at 1.7 nm, *J*_1.7_, and the lower panels show the size distributions (d*N*/dlog*D*_p_) of negatively charged particles of mobility diameter between 0.8 and 42.0 nm. MSA was absent for the left-hand panels (**a**–**c**) and present for the right-hand panels (**d**–**f**). The experiments were performed with NH_3_ below the limit of detection, with estimated contaminant concentrations <1 pptv. All nucleation rates, *J*_1.7_, are consistent with those expected for SA-NH_3_ with 1–4 pptv NH_3_ (grey bands in panels **b** and **e**). Addition of MSA has a negligible influence on nucleation rates, *J*_1.7_ (panels **b** versus **e**), but contributes equally to SA for particle growth rates above 3.2 nm (panels **c** versus **f**). These experiments show that MSA is unable to contribute to nucleation in the absence of base vapours such as NH_3_, even at temperatures as low as −52 °C. However, MSA can contribute to particle growth under these conditions, as the Kelvin (curvature) barrier falls away. The experimental conditions for panels **a**–**c** are 320 ppbv O_3_, 6.1 ppbv SO_2_ and 71% RH. SA was produced in this experiment from the oxidation of SO_2_. The experimental conditions for panels **d**–**f** are 1,584 ppbv O_3_, 0.07 ppbv SO_2_, 6.5 ppbv DMS and 70% RH. MSA and SA were generated in this experiment through the oxidation of DMS.

We also conducted experiments at −52 °C without ammonia (< 1 pptv contaminant level) to simulate upper tropospheric conditions. Extended Data Fig. 2 presents an experiment with 5 × 10^6^ cm^−3^ SA, both before (Extended Data Fig. 2a–c) and after (Extended Data Fig. 2d–f) adding 5 × 10^6^ cm^−3^ MSA. The measured nucleation rate, *J*_1.7_, did not increase, despite doubling the acid concentration (Extended Data Fig. 2b,e). However, the particle growth rate above 3.2 nm was doubled (Extended Data Fig. 2c,f), reaching the kinetically limited condensation rate. The lack of any enhancement of the nucleation rate by MSA in the near absence of NH_3_ contrasts with the strong enhancements measured at −10 °C and −30 °C in the presence of NH_3_ (Fig. 1), despite a reduction of MSA saturation vapour pressure by at least an order of magnitude between −30 °C and −52 °C. We also conducted a sensitivity test in which MSA was directly injected into the chamber at concentrations up to 1.4 × 10^7^ cm^−3^ at −30 °C, in the absence of NH_3_ and SA. No nucleation was observed until NH_3_ was introduced to the chamber. These experiments show that MSA at concentrations below around 10^7^ cm^−3^ does not contribute to particle nucleation in the atmosphere in the absence of a base vapour, even in the presence of SA and at temperatures as low as −52 °C.

## Composition of molecular clusters

**Fig. 2:**
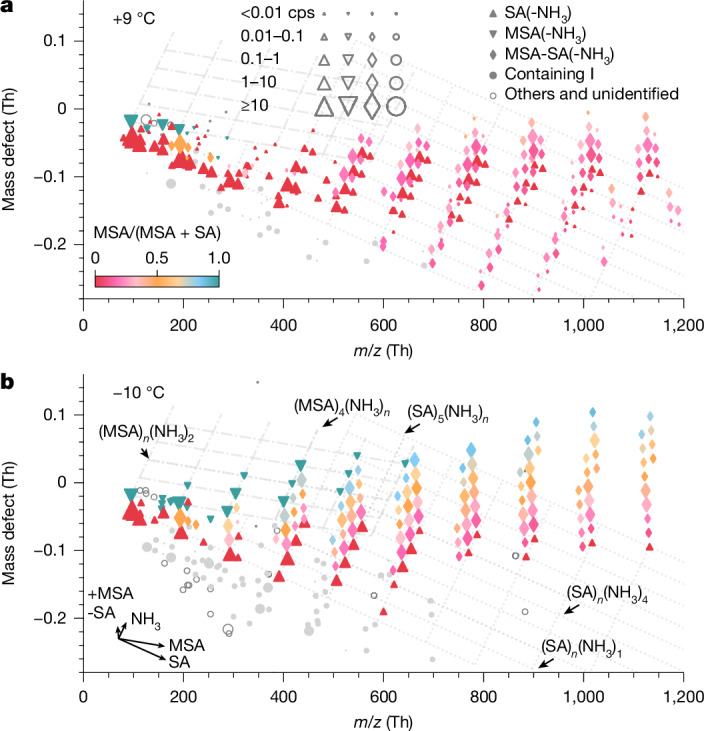
Molecular composition of negatively charged clusters during MSA-SA-NH_3_ nucleation. **a**,**b**, Mass defect (difference from integer mass) versus *m*/*z* (Th) during nucleation events at +9 °C (**a**) and −10 °C (**b**) for negatively charged molecular clusters measured with the APi-TOF mass spectrometer. The marker size shows ion signal intensity in counts per second (cps), as indicated in the legend. The vector arrows and dotted grid lines indicate the molecular increments and cluster locations, respectively. The symbol shape indicates the chemical composition: SA-NH_3_ (triangles), MSA-NH_3_ (inverted triangles) and MSA-SA-NH_3_ (diamonds). Clusters that contain contaminant iodine from earlier experiments are indicated by filled grey circles. The symbol colour indicates the MSA molar fraction, MSA/(MSA + SA). The experimental conditions for **a** are 1.0 × 10^7^ cm^−3^ MSA, 1.6 × 10^7^ cm^−3^ SA, 1.2 × 10^6^ cm^−3^ IA, 771 pptv DMS, 123 pptv NH_3_, 253 ppbv O_3_, 0.7 ppbv SO_2_ and 42% RH. The experimental conditions for **b** are 1.1 × 10^7^ cm^−3^ MSA, 0.8 × 10^7^ cm^−3^ SA, 8.3 × 10^5^ cm^−3^ IA, 30 pptv DMS, 71 pptv NH_3_, 7.6 ppbv O_3_, 0.5 ppbv SO_2_ and 65% RH.

Figure 2 presents the molecular composition of negatively charged clusters during MSA-SA-NH_3_ nucleation at +9 °C (Fig. 2a) and −10 °C (Fig. 2b), measured with an APi-TOF mass spectrometer. The vapour concentrations were comparable for both experiments: 1.0 × 10^7^ cm^−3^ MSA, 1.6 × 10^7^ cm^−3^ SA and 123 pptv NH_3_ (Fig. 2a) and 1.1 × 10^7^ cm^−3^ MSA, 0.8 × 10^7^ cm^−3^ SA and 71 pptv NH_3_ (Fig. 2b). Contaminant iodine oxoacid was also present from previous measurements at concentrations around 10^6^ cm^−3^ but represented a minor nucleation channel. The clusters contain up to ten SA, eight MSA and nine NH_3_ molecules and reveal 1:1 acid–base pairing as the nucleation mechanism^3,42^.

**Extended Data Fig. 3:**
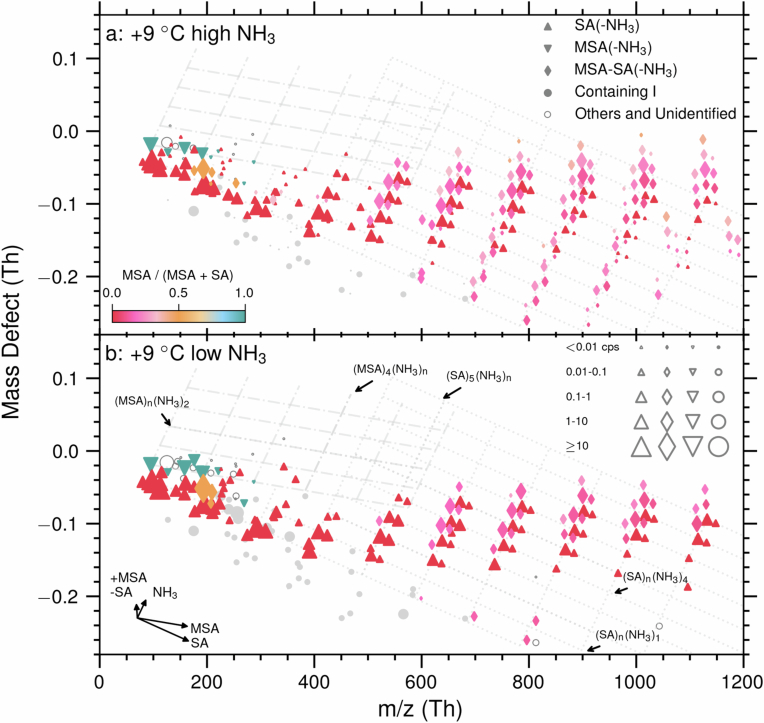
Molecular composition of negatively charged clusters during MSA-(IA_*x*_)-SA-NH_3_ nucleation at +9 °C at varying NH_3_ concentration. Mass defect versus *m*/*z* during nucleation events at 123 pptv NH_3_ (**a**) and 43 pptv NH_3_ (**b**) for negatively charged molecular clusters measured with the APi-TOF. The marker size shows ion signal intensity in counts per second (cps), as indicated in the legend. The symbol shape indicates the chemical composition. The symbol colour indicates the MSA content, expressed as MSA/(MSA + SA). MSA was generated in both experiments through the oxidation of DMS. The experimental conditions for panel **a** are 1.04 × 10^7^ cm^−3^ MSA, 1.6 × 10^7^ cm^−3^ SA, 1.2 × 10^6^ cm^−3^ IA, 771 pptv DMS, 123 pptv NH_3_, 253 ppbv O_3_, 0.7 ppbv SO_2_ and 42% RH. The experimental conditions for panel **b** are 1.4 × 10^7^ cm^−3^ MSA, 2 × 10^7^ cm^−3^ SA, 6.8 × 10^5^ cm^−3^ IA, 827 pptv DMS, 43 pptv NH_3_, 261 ppbv O_3_, 0.72 ppbv SO_2_ and 42% RH.

At +9 °C (Fig. 2a), MSA was detected as a monomer and a dimer but did not appear in larger clusters until at least five SA molecules had accreted with NH_3_, supporting our earlier conclusion that MSA does not contribute to nucleation under these conditions. To further explore the nucleation potential of MSA-NH_3_ at +9 °C in the absence of SA, we performed further experiments in which elevated MSA (up to 2 × 10^8^ cm^−3^) was directly injected into the chamber at up to 190 pptv NH_3_ and 60% RH. No particle formation was observed, confirming that MSA-NH_3_ cannot drive nucleation at +9 °C. Nevertheless, in the experiment shown in Fig. 2a, MSA was detected in clusters containing five SA, showing that MSA can contribute to particle growth immediately after the nucleation barrier represented by the negatively charged SA tetramer (*m*/*z* = 400–450 Th in Fig. 2a) has been crossed. Further insights are provided in Extended Data Fig. 3, which compares the molecular composition of the experiment in Fig. 2a with another experiment at +9 °C with similar MSA concentration but three times lower NH_3_ concentration (123 pptv versus 43 pptv). The lower NH_3_ produces a marked reduction of MSA in (SA)_5_(NH_3_)_*n*_ clusters, indicating that MSA is rapidly evaporating from the five SA clusters at +9 °C and is highly sensitive to NH_3_ concentration, even at base:acid ratios of more than 100. We infer from these molecular measurements that the temperature at which MSA begins to nucleate under atmospheric conditions is not far below +9 °C and it requires the presence of relatively high NH_3_ concentrations.

**Extended Data Fig. 4:**
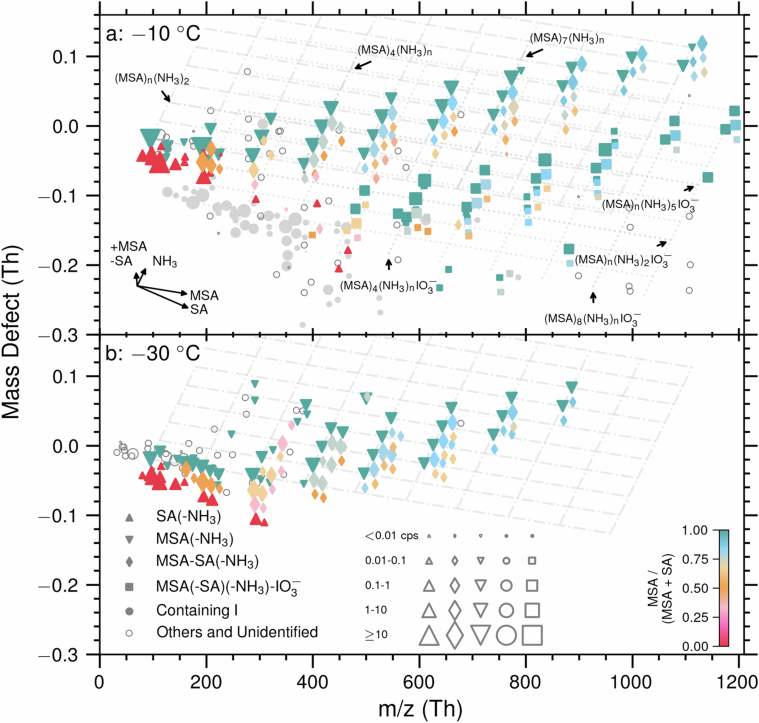
Molecular composition of negatively charged clusters during MSA-(IA_*x*_)-SA-NH_3_ nucleation at high MSA:SA ratio. Mass defect versus *m/z* during nucleation events at −10 °C (**a**) and −30 °C (**b**) for negatively charged molecular clusters measured with the APi-TOF and MION2-APi-TOF. The marker size shows ion signal intensity in counts per second (cps), as indicated in the legend. The symbol shape indicates the chemical composition. The symbol colour indicates the MSA content, expressed as MSA/(MSA + SA). MSA was generated in both experiments through the oxidation of DMS. The experimental conditions for panel **a** are 8.1 × 10^6^ cm^−3^ MSA, 5 × 10^5^ cm^−3^ SA, 1.8 × 10^6^ cm^−3^ IA, 2.9 ppbv DMS, 88 pptv NH_3_, 122 ppbv O_3_, 0.5 ppbv SO_2_ and 68% RH. The experimental conditions for panel **b** are 5.8 × 10^6^ cm^−3^ MSA, 4.5 × 10^5^ cm^−3^ SA, 2.7 ppbv DMS, 47 ppbv O_3_, 0.06 ppbv SO_2_ and 63% RH. NH_3_ was estimated from MFC settings to be between 4 and 119 ppbv (see Methods for further details).

The cluster composition is very different at −10 °C (Fig. 2b). MSA is now seen in the initial nucleating clusters and a clear sequence of pure MSA-NH_3_ clusters is observed (inverted green triangles), extending up to (CH_3_SO_3_H)_5_·(NH_3_)_4_·CH_3_SO_3_^−^. This demonstrates that ion-induced nucleation of MSA-NH_3_ takes place at −10 °C, without requiring SA. The first MSA cluster with NH_3_ is the dimer, that is, CH_3_SO_3_H·NH_3_·CH_3_SO_3_^−^, whereas the first SA cluster with appreciable NH_3_ is the tetramer, that is, (H_2_SO_4_)_3_·NH_3_·HSO_4_^−^, which is probably because of CH_3_SO_3_^−^ being a weaker Lewis base than HSO_4_^−^. Pure SA-NH_3_ clusters are also detected (red triangles). However, the dominant nucleation pathway involves multi-acid MSA-SA-NH_3_ clusters (diamonds), which follow a 1:1 acid:base ratio, in which acid is the sum of MSA and SA and they become increasingly abundant as the clusters grow. This illustrates the symbiotic and freely mixing nature of these two acids in forming molecular clusters at −10 °C and below. To further explore the effect of acid composition, we increased the MSA:SA ratio from 1.4:1 (Fig. 2b) to 16:1 at −10 °C and to 13:1 at −30 °C (Extended Data Fig. 4a,b, respectively). At these high MSA:SA ratios, the dominant nucleation mechanism becomes MSA(-NH_3_), with only a minor presence of SA in the clusters, once again demonstrating the interchangeability and synergy between MSA and SA in multi-acid nucleation.

The experiments shown in Extended Data Fig. 4a included contaminant iodine oxoacids (1.8 × 10^6^ cm^−3^ IA and 9.6 × 10^4^ cm^−3^ IOA), leading to the formation of IA-MSA-NH_3_ and IA-MSA-SA-NH_3_ clusters (squares). Here IA may either provide the core ion (IO_3_^−^) or else assist cluster formation as a neutral molecule. Notably, our nitrate chemical ionization mass spectrometer (CIMS) measurements revealed a prominent MSA-IOA dimer, indicating that IOA may substitute for ammonia, as seen previously for SA-IA nucleation^40,41^. This could be important for low-NH_3_ environments, such as the upper free troposphere and remote polar regions, away from localized sources such as seabird colonies. Quantifying the kinetics and stability of MSA-IOA clusters will be critical to understanding their atmospheric relevance.

## Particle growth rates

**Fig. 3:**
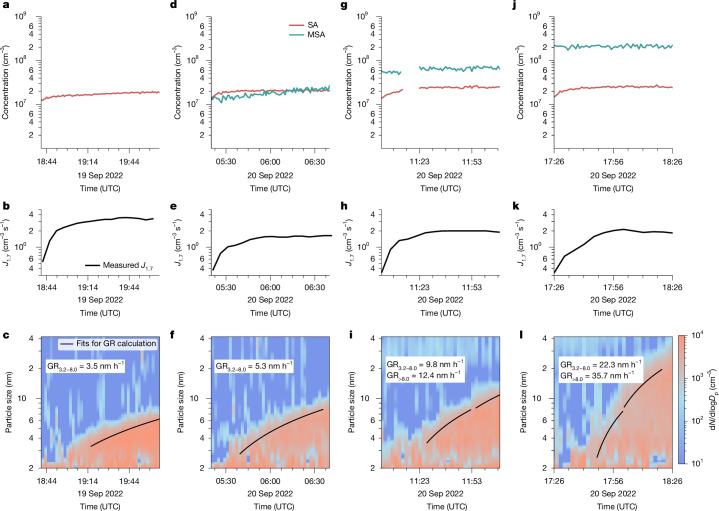
Influence of MSA on particle growth rates at +9 °C. **a**,**d**,**g**,**j**, Evolution of the SA and MSA concentrations for four experiments at constant SA and increasing MSA. **b**,**e**,**h**,**k**, Particle nucleation rates at 1.7 nm, *J*_1.7_. **c**,**f**,**i**,**l**, Corresponding particle size distributions (d*N*/dlog*D*_p_) between 2 and 42 nm. For these experiments, SA was generated by oxidation of SO_2_, while MSA vapour was directly injected into the chamber from an evaporator so that it could be adjusted without changing SA. The base vapours for these experiments consisted of <1 pptv contaminant DMA and <4 pptv contaminant NH_3_. The experimental conditions for **a**–**c** are 38 ppbv O_3_, 4.9 ppbv SO_2_ and 63% RH. The experimental conditions for **d**–**f** are 38 ppbv O_3_, 6.4 ppbv SO_2_ and 62% RH. The experimental conditions for **g**–**i** are 39 ppbv O_3_, 6.7 ppbv SO_2_ and 62% RH. The experimental conditions for **j**–**l** are 44 ppbv O_3_, 6.3 ppbv SO_2_ and 62% RH. The UV intensity was held constant during all experiments.

In Fig. 3, we show four experiments to study particle growth at +9 °C and constant SA (2 × 10^7^ cm^−^^3^) but with different MSA concentrations: <3 × 10^5^ cm^−3^ (Fig. 3a), 2 × 10^7^ cm^−3^ (Fig. 3d), 7 × 10^7^ cm^−3^ (Fig. 3g) and 2 × 10^8^ cm^−3^ (Fig. 3j). Here we injected MSA vapour directly from an evaporator, so its concentration could be adjusted without changing SA. For these experiments, the base vapours consisted of around <1 pptv contaminant DMA plus <4 pptv NH_3_. The particle growth rates between 3.2 and 8.0 nm (GR_3.2–8.0_) increased progressively from 3.5 nm h^−^^1^ with low MSA (Fig. 3c) to 5.3, 9.8 and 22.3 nm h^−^^1^ (Fig. 3f,i,l, respectively). These growth rates correspond to 0.9–0.96 nm h^−^^1^ per 10^7^ cm^−^^3^ of MSA, which indicates kinetically limited growth by MSA condensation, as we previously measured for SA^43^ and IA^41^.

**Fig. 4:**
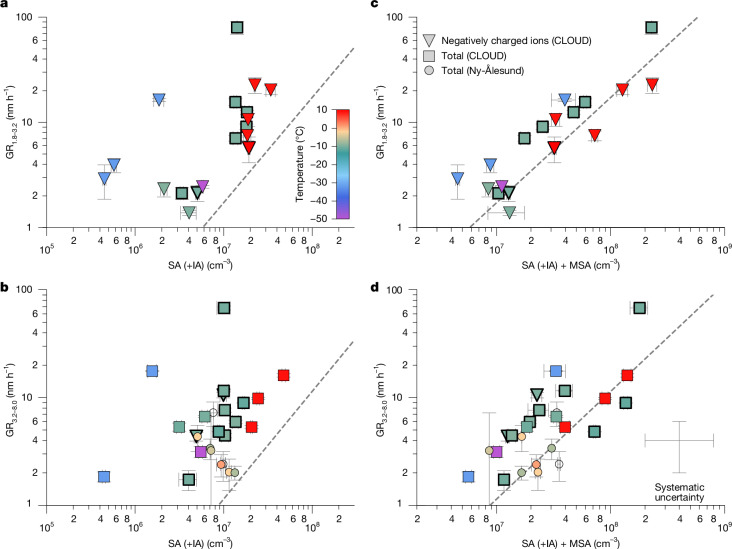
Growth rates versus acid concentrations. **a**–**d**, Particle growth rates versus SA (+IA) (**a**,**b**) and SA (+IA) + MSA (**c**,**d**) concentrations. The ‘(+IA)’ is enclosed in parentheses to denote that it was only present in certain experiments (indicated by symbols with a thick outline). The upper panels show the growth rates between 1.8 and 3.2 nm, whereas the bottom panels show the growth rates between 3.2 and 8.0 nm. The CLOUD measurements are represented by inverted triangles and squares (corresponding to negatively charged and total particles, respectively). For comparison, ambient observations from Ny-Ålesund are represented by circles (total particles). The symbol colour indicates temperature. Open circles indicate that temperature measurements were not available from Ny-Ålesund. The dashed lines show the theoretical growth rates for SA condensation at the kinetic limit, after accounting for van der Waals interactions, which we have verified experimentally^43^. The alignment of all measurements near the dashed lines when all acids are summed (**c**,**d**) indicates that MSA drives particle growth above 1.8 nm at or near the kinetic limit for all temperatures below +9 °C. Each data point represents the growth rate measured in a single experiment. The acid concentrations plotted on the *x* axis are the mean values measured during the corresponding growth period. Vertical error bars represent ±1*σ* statistical uncertainties in the growth rate measurement, whereas the horizontal error bars represent ±1*σ* uncertainty in vapour concentrations. The systematic scale uncertainties for acid concentrations (50%, 200%) and growth rates (±50%) are shown separately in panel **d**. The experimental condition ranges are 40–79% RH, 0–1.8 × 10^7^ cm^−^^3^ IA, <1 to 492 pptv NH_3_, 4–255 ppbv O_3_ and 0–7 ppbv SO_2_. Ammonia was not measured during the −30 °C experiments but instead estimated using a box model (Methods).

In Fig. 4, we show all of our measurements of particle growth rates with various mixtures of SA, IA_*x*_ and MSA between −52 °C and +9 °C. The upper panels show growth rates from 1.8 to 3.2 nm and the lower panels from 3.2 to 8.0 nm. In Fig. 4a,b, the measured growth rates greatly exceed those expected solely from kinetic condensation of SA and IA_*x*_ (indicated by the dashed lines). However, once MSA is included in the total acid concentration, the measurements either align with or approach the kinetic limit (Fig. 4c,d), indicating that MSA drives particle growth above 1.8 nm size at or near the kinetic limit, below +9 °C. Growth rates measured above the kinetic limit result from the accretion of acid–base molecular clusters at base:acid ratio >1, which are unaccounted by the acid monomer concentration (*x* axis)^41^. Two measurements (green squares) at −10 °C that fall slightly below the kinetic line in Fig. 4d correspond to experiments with base:acid ratios near or below unity (NH_3_ < 4 pptv) and with nucleation rates below 10 cm^−3^ s^−1^. Under these conditions, molecular clusters are not expected to contribute substantially to particle growth rate. The situation is similar at +9 °C because, at this temperature, MSA does not contribute appreciably to small molecular clusters. In summary, our measurements show that MSA drives particle growth at or near the kinetic limit across the full temperature range from −52 °C to +9 °C, even at extremely low NH_3_ concentrations with a base:acid ratio below unity.

We have also compared our growth rate measurements with ambient observations from Ny-Ålesund, Svalbard (circle symbols in Fig. 4b,d). To enable a direct comparison with our results, we reanalysed the ambient data^21^, to extract the growth rates for 3.2–8.0 nm. The measured growth rates during spring in Ny-Ålesund exceed those expected from SA alone and closely match our experimental results, within measurement uncertainties, supporting the important role of MSA for particle growth in polar environments.

**Extended Data Fig. 5:**
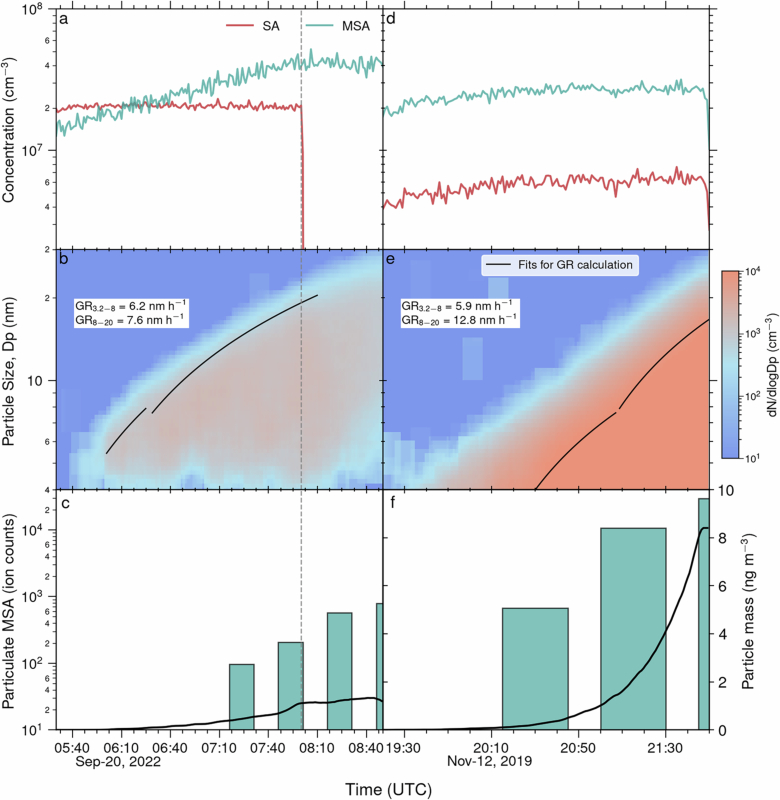
Measurement of particle composition below 10 nm. Evolution of vapour concentrations (upper panels), particle size distributions (d*N*/dlog*D*_p_) for mobility diameters between 4 and 30 nm (middle panels, measured by the nanoSMPS) and particulate MSA signals and mass concentrations (lower panels, measured by the FIGAERO-CIMS). The experiments were performed at +9 °C (**a**–**c**) and −10 °C (**d**–**f**). MSA was either directly injected into the chamber (**a**–**c**) or else generated by oxidation of DMS (**d**–**f**). The bin widths in panels **c** and **f** represent the periods when particles were collected on the filters of the FIGAERO-CIMS. The dashed vertical line in the left-hand panels indicates when the UV illumination was switched off and SA production ended; nevertheless, MSA continued to drive particle growth. At −10 °C, MSA was detected in the particle phase using a Br-FIGAERO-CIMS when the particles were all below 10 nm (panel **f**). Although SA was measured in the particle phase, it is not included in panels **c** and **f** because direct comparison with MSA requires compound-specific calibrations, which were not available. The experimental conditions for panels **a**–**c** are 38 ppbv O_3_, 6.4 ppbv SO_2_, <4 pptv NH_3_, <1 pptv DMA and 62% RH. The experimental conditions for panels **d**–**f** are 247 ppbv O_3_, 0.6 ppbv SO_2_, 491 pptv DMS, 472 pptv NH_3_ and 73% RH.

Finally, we have directly confirmed the presence of MSA in the particle phase by analysing thermally evaporated particles with a Filter Inlet for Gases and AEROsols (FIGAERO) coupled to a CIMS. Extended Data Fig. 5 shows our measurements at +9 °C (Extended Data Fig. 5a–c) and −10 °C (Extended Data Fig. 5d–f). MSA is detected in particles smaller than 10 nm and at particle mass concentrations of only 1 ng m^−^^3^ (Extended Data Fig. 5c,f). The production of SA is turned off during the experiment at +9 °C but the particles continue to grow and particulate MSA continues to increase (Extended Data Fig. 5a–c), demonstrating that MSA can drive particle growth independently of SA. The only particle-phase compounds detected by the FIGAERO in these experiments were SA and MSA. This provides direct evidence that particle growth is driven by SA and MSA alone rather than by some other, unidentified compound.

## Atmospheric implications

In summary, we find that MSA nucleates together with SA-NH_3_ at −10 °C and below, at atmospheric concentrations. Moreover, MSA and SA show comparable strengths, demonstrating their interchangeability and synergy when quantitatively assessing acid–base nucleation rates. However, at slightly warmer temperatures (+9 °C), MSA does not nucleate with NH_3_, although our molecular measurements suggest that the onset of nucleation is not far below +9 °C. Furthermore, MSA does not nucleate in the absence of a base vapour. We also find that MSA drives rapid growth of particles above 1.8 nm at or near the kinetic limit across the full temperature range from −52 °C to +9 °C. Rapid growth rates persist even at extremely low NH_3_ concentrations, with base:acid ratios well below unity, indicating that NH_3_ is not required for particle growth.

**Fig. 5:**
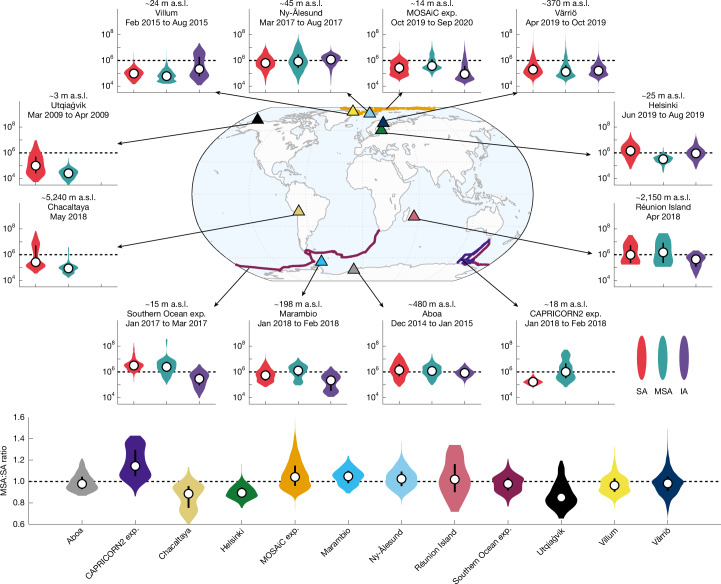
Global SA, MSA and IA observations from ground stations and ship expeditions. Violin plots showing ground-based and ship-based measurements of SA (red), MSA (green) and IA (purple) at the observatories or along the ship paths indicated on the map. Dates and locations (or titles) are indicated on each plot. The lower plot shows the simultaneous MSA:SA ratio for each site, with a linear *y* scale. The width of a violin distribution is proportional to the frequency of observation at the corresponding vapour concentration indicated on the *y*-axis. The white circles represent the median of the distribution and the attached vertical lines span the 25th to 75th percentiles. All data presented here have hourly time resolution. Details of the measurement sites and data sources are provided in Methods.

**Extended Data Fig. 6:**
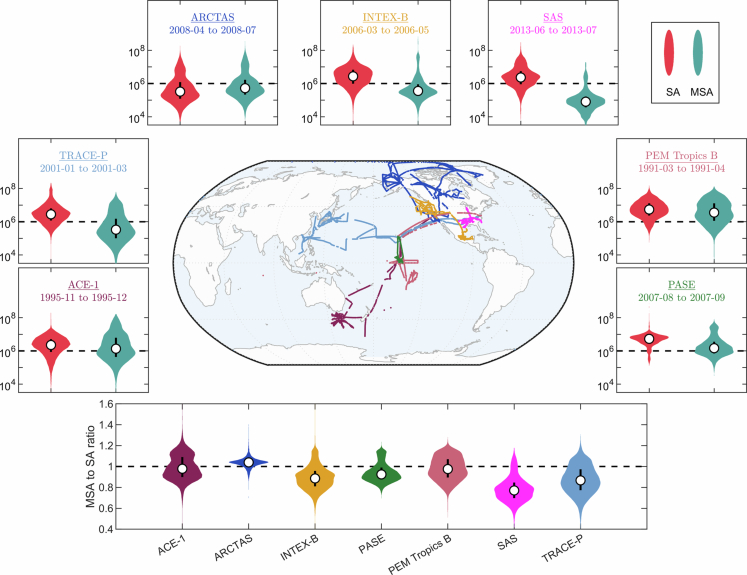
Global SA and MSA observations from aircraft measurements. Violin plots showing airborne measurements of SA (red) and MSA (green) along the flight paths indicated on the map. Dates and campaign names are indicated on each plot. The lower plot shows the simultaneous MSA:SA ratio for each campaign, with a linear *y* scale. The width of each violin is proportional to the frequency of observation at the corresponding vapour concentration indicated on the *y*-axis. The white circles represent the median and the attached vertical bars denote the interquartile range (25th to 75th percentiles). Details on the flight campaigns and data sources are provided in Methods.

Together, our findings therefore suggest that MSA should be considered as being comparable with SA when evaluating acid–base new particle formation in cool regions of the marine atmosphere. To assess the potential impact of MSA on new particle formation, we have assembled global measurements of SA, MSA and IA in cool marine regions, made from ground stations and ships (Fig. 5) and from aircraft (Extended Data Fig. 6). These vapours generally coexist at all of these locations with concentrations between 10^4^ and 10^7^ cm^−3^. Moreover, the MSA and SA concentrations are closely matched, with simultaneous MSA:SA ratios predominantly in a narrow range between 0.7 and 1.3 (bottom panels in Fig. 5 and Extended Data Fig. 6). When combined with our results, it implies that present model studies may be underestimating the effective acid concentrations that drive particle nucleation and growth in cool marine regions by around a factor of 2, which translates to underestimating the nucleation rates by an order of magnitude and growth rates by twofold.

This conclusion for nucleation rates depends on the presence of base vapours. However, we note that SA-NH_3_ ternary nucleation is also considered to be the dominant mechanism for SA-driven nucleation in the remote atmosphere^44^. An important source of NH_3_ in polar regions is guano from ubiquitous seabird colonies, which can produce between 10 and 1,000 pptv NH_3_ in both the Arctic^45^ and Antarctic^44^ boundary layers. The ammonia emissions persist even after migration of the birds, as they leave behind so-called ornithogenic soils, enriched with guano and vast amounts of organic matter and nutrients brought from the ocean to the land. In the Arctic, long-range transport of anthropogenic emissions can further increase NH_3_ levels^46^. In the cold temperatures of the free troposphere, MSA is produced with a high yield from DMS oxidation^2^ and can then drive new particle formation after mixing with NH_3_ convectively transported from the boundary layer.

On the other hand, even in the absence of base vapours, we find that MSA accelerates the growth rates of particles above 1.8 nm in size, below +9 °C. This may be important for CCN concentrations over extensive regions of the cool boundary layer and free troposphere, because the main loss mechanism of small, highly mobile new particles is scavenging by pre-existing larger particles. Accelerated growth rates will result in reduced losses of the newly formed particles and a higher likelihood that they survive to reach CCN sizes.

**Extended Data Fig. 7:**
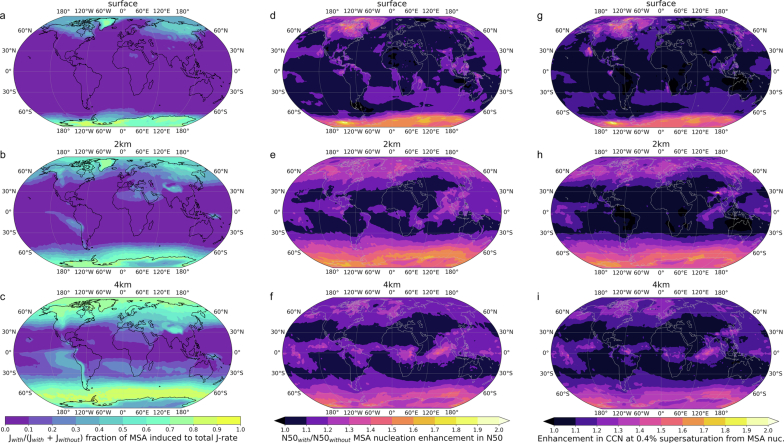
Yearly averaged contribution of MSA to particle nucleation rates, N50 and CCN concentration simulated with the EMAC model. Panels **a**–**c** show the fraction of the total particle nucleation rates (*J*) attributable to MSA (*J*_with MSA_/(*J*_with MSA_ + *J*_without MSA_)) at the surface (**a**), 2 km altitude (**b**) and 4 km altitude (**c**) for the period 2016–2019. Panels **d**–**f** show the corresponding yearly averaged enhancement in N50 concentration owing to MSA at the surface (**d**), 2 km altitude (**e**) and 4 km altitude (**f**) for the period 2016–2019. Panels **g**–**i** show the corresponding yearly averaged enhancement in CCN concentration at 0.4% supersaturation owing to MSA at the surface (**g**), 2 km altitude (**h**) and 4 km altitude (**i**) for the period 2016–2019. The CCN enhancement ratio represents CCN concentrations relative to the baseline simulation, after accounting for SA-MSA-NH_3_ nucleation and MSA-driven particle growth measured in this study (see Methods for details). The baseline simulation considers only SA(-NH_3_) particle formation, calculated according to the Dunne et al. parameterization^28^, together with all primary aerosol emissions^103^.

To evaluate the impact of MSA on global new particle formation, we have carried out an initial study with the EMAC (ECHAM/MESSy Atmospheric Chemistry) Earth system model (Extended Data Fig. 7). Our baseline simulation considers only SA(-NH_3_) nucleation^28^, (labelled ‘without’ MSA). We compare this with a simulation that adds MSA-SA-NH_3_ nucleation (labelled ‘with’ MSA). Here the sum of MSA and SA drives nucleation when the temperature is below −10 °C and [NH_3_] > 3 × [MSA]; outside these conditions, MSA does not contribute to nucleation. Under conditions in which MSA volatility is low^5^, MSA is considered to contribute equally with SA to particle growth, whereas it is treated as semi-volatile at higher temperatures and dry conditions (see Methods for further details). Our simulations indicate that MSA enhances nucleation rates and CCN concentrations especially strongly in the Arctic and Antarctic coastal regions, from the boundary layer up to at least 4 km altitude (Extended Data Fig. 7). These results are supported by recent observations of elevated gaseous MSA from aerosol evaporation during katabatic outflow from the Antarctic continent, which draws in upper tropospheric air from over the Southern Ocean^25^. Earth system models systematically underestimate CCN by more than 50% over the Southern Ocean, producing a large positive radiation bias in this climatically important region^47,48^. Our findings suggest that inclusion of MSA-driven new particle formation, which is not considered in present climate models, may substantially reduce their radiation bias in the Southern Ocean.

Our results indicate that purely biogenic marine sources produced more CCN in the pristine pre-industrial marine atmosphere than thought at present. Evaluating the influence of MSA on the baseline pre-industrial aerosol state will require careful consideration of the effects of cooler temperatures, which would enhance the yield of MSA from DMS compared with the present day, and of a greater extent of sea ice, which would displace phytoplankton and their DMS emissions. Increased biogenic CCN will tend to reduce estimates of Earth’s effective radiative forcing from present-day anthropogenic aerosol and, in turn, affect projections of warming later this century. SO_2_ emissions are now decreasing, which may return the future aerosol state of the atmosphere towards pre-industrial levels, buffered by biogenic particle formation in a warmer climate. Although future globally averaged seawater DMS concentrations are predicted to decrease, emissions of DMS into the atmosphere are expected to increase as a result of rising surface wind speeds and sea surface temperatures^49^. Together with falling SO_2_ levels, this suggests an increasing importance of MSA and of ammonia emissions for aerosol effective radiative forcing in the post fossil fuel climate.

## Methods

### The CLOUD chamber

The experiments presented in this study were performed in the CERN CLOUD chamber. CLOUD is a cylindrical stainless-steel chamber measuring approximately 3.5 m in height and 2.5 m in diameter, with a total volume of 26.1 m^3^. It provides a controlled environment for studying the nucleation and growth processes of aerosol particles featuring an advanced environmental control system that regulates temperature, humidity and trace gas concentrations across the full range of boundary layer, tropospheric and lower-stratospheric conditions. The thermal housing surrounding the chamber ensures uniformity and stability of the chamber temperature over a wide range. In this study, experiments were conducted at temperatures of +9 to 10 °C, −10 °C, −30 °C and −52 °C. The chamber is operated in a continuous mode with ultrapure synthetic air, produced by mixing cryogenic liquids of oxygen (21% O_2_) and nitrogen (79% N_2_), continuously injected into the chamber. The gas-handling system is equipped with a variety of mass flow controllers (MFCs) covering different ranges and several dilution steps that enables the precise control and injection of trace gases at the desired level. Sulfur dioxide (100 ppmv SO_2_ in N_2_), ammonia (1% NH_3_ in N_2_), carbon monoxide (3% CO in N_2_) and dimethyl sulfide (1,000 ppmv DMS in N_2_) were injected into the chamber from pressurized gas cylinders, whereas iodine (I_2_) and MSA were injected into the chamber from a temperature-controlled evaporator containing crystalline iodine (Sigma-Aldrich, 99.999% purity) and liquid MSA (Sigma-Aldrich, >99.95% purity), respectively. Ozone (O_3_) was introduced into the chamber by passing synthetic air through an ozone generator. O_3_ was photolysed in the chamber to produce OH radical using four 200-W Hamamatsu mercury–xenon ultraviolet (UV) lamps of wavelength range between 250 and 450 nm or using a xenon fluoride excimer laser (UVX) at 248 nm. To prevent cross-contamination, all gases are delivered through independent gas lines. Further, the chamber undergoes a thorough cleaning process involving rinsing the chamber walls with ultrapure water and subsequent heating to 373 K for more than 24 h in between different experimental programmes to maintain minimal cross-contamination between different runs.

The chamber is also operated at different ionization levels. In neutral (N) experiments, a powerful electric field (±30 kV) is applied, rapidly eliminating all small ions from the chamber within a second to establish an ion-free environment. In ground-level GCR experiments, the natural ionization from cosmic rays is maintained. Furthermore, in BEAM experiments, the chamber is exposed to a 3.5 GeV *c*^−1^ secondary pion beam from the CERN Proton Synchrotron, which substantially increases the ionization to levels akin to those observed in the upper troposphere–lower stratosphere. The experiments presented here were performed largely at GCR conditions, with few experiments performed at neutral conditions. Uniform mixing of particles and vapours within the chamber is achieved using two magnetically coupled stainless-steel fans positioned at the top and bottom of the chamber. This set-up ensures rapid and efficient mixing within a few minutes.

The data presented in this study were collected during CLOUD 13, CLOUD 14, CLOUD 15 and two experiments from CLOUD 16. The CLOUD 13 and CLOUD 14 campaigns were conducted consecutively in 2018 and 2019, whereas CLOUD 15 and CLOUD 16 took place in 2022 and 2023, following the renovation of the building housing the CLOUD chamber. The renovation focused on upgrading the facilities in the building, whereas the CLOUD chamber itself remained unchanged. To ensure consistency, pure SA and iodine oxoacid new particle formation experiments were conducted during each campaign. The results were compared with well-established parameterizations, confirming the consistency of the CLOUD chamber before and after the renovation.

### The new particle formation experiments

To investigate the role of MSA in new particle formation, we conducted experiments at different temperatures. Before each experiment, precursor gases (O_3_, SO_2_, DMS) were introduced into the chamber and allowed to stabilize, with fans operating at maximum speed (100%) to ensure efficient turbulent mixing and light illumination switched off. Once particle-free conditions and stable precursor concentrations were established, the experiments were initiated by reducing the fan speed to 12% and turning on the lights. This triggered photochemical OH radical production, leading to the oxidation of DMS and the subsequent formation of SA and MSA. Varying the MSA:SA ratio from DMS oxidation over a wide range is challenging, particularly for achieving higher MSA:SA ratios at warmer temperatures. To address this, further experiments were conducted at +9 °C, at which MSA was directly injected into the chamber from an evaporator, while SA was generated through the photo-oxidation of SO_2_. Following particle generation in the chamber, the experiment was continued until a stable particle nucleation rate (for nucleation rate experiments) or the desired particle size growth (for growth rate experiments) was achieved.

During the MSA injection experiments at +9 °C, high concentrations of MSA may accumulate near the bottom of the chamber before mixing throughout its total volume. To evaluate the effect of this phenomenon, blank experiments with pure MSA injection were conducted, confirming that no particles formed in the absence of SA. Furthermore, in some experiments, a small amount of iodine was added alongside MSA and SA to simulate the marine and polar atmospheric conditions. During these experiments, a green light (528 nm) was used to photolyse I_2_ into iodine atoms, which were then rapidly oxidized in the presence of water vapour and ozone, leading to the formation of both IA and IOA.

Throughout the experiments, environmental conditions, trace gas concentrations, particle concentrations and composition were continuously monitored in real time, as explained in subsequent sections. To quickly clean the chamber between experiments at the same temperature, we turned off the lights or gas injection, increased the fan speed to 100%, activated the high-voltage clearing field in alternating field mode and sometimes increased the ionization rate for small particles to facilitate their removal by the alternating field. The experiments were always conducted from the highest to the lowest temperatures to prevent contamination release from the chamber walls.

### Instrumentation

#### SA, MSA and iodine species

The concentrations of SA, MSA, IOA and IA are critical in this study and were monitored using the nitrate CIMS (NO_3_-CIMS; TOFWERK AG) coupled with a corona nitrate ion source^50^ and the bromide CIMS coupled with a multi-scheme chemical-ionization inlet (Br-MION-CIMS)^51,52^. The NO_3_-CIMS, identical across the CLOUD 13, CLOUD 14, CLOUD 15 and CLOUD 16 campaigns, is described in detail in a previous publication^53^. The Br-MION-CIMS was not used during CLOUD 14 and we used different instruments and inlets for CLOUD 13 (Br-MION1-CIMS) and CLOUD 15 and CLOUD 16 (Br-MION2-CIMS), although the underlying principles are identical. Therefore, we relied on the NO_3_-CIMS measurements for the reported concentrations of SA, MSA, IA and IOA, with cross-checks against Br-MION-CIMS or against parameterizations of new particle formation from iodine oxoacid (IA_*x*_)^40,41^.

For CLOUD 13, CLOUD 15 and CLOUD 16, we performed several SA calibration experiments using two independent calibration systems before, during and after the campaigns^54^. Although the instrument is mainly calibrated for SA, the same calibration factor is applied to quantify IA^55^, IOA^40^ and MSA^2^, as extensive research has shown that these compounds can be detected at the collision limit. After applying the respective calibration factors, we compared the measured concentrations of SA, MSA and IA from both instruments across different temperatures. These comparisons demonstrated strong consistency, validating the accuracy of the SA calibrations. The calibration coefficients applied for the CLOUD 13 and CLOUD 15 campaigns were 1.1 × 10^10^ and 6.2 × 10^9^ cm^−3^ per normalized signal (cps cps^−1^), with uncertainties of 50–150% (ref. ^2^) and 88–164% (ref. ^56^), respectively. During CLOUD 16, two SA calibration experiments were performed before and after the experiments, yielding calibration factors of 1.25 × 10^10^ and 1.85 × 10^10^ cm^−3^ cps cps^−1^. An independent SA calibration of the Br-MION2-CIMS yielded a calibration factor that was 74% of the lower value (1.25 × 10^10^ cm^−3^ cps cps^−1^). Accordingly, we used a final SA calibration factor of 1.25 × 10^10^ cm^−3^ cps cps^−1^ with an estimated uncertainty of 74–148%.

During CLOUD 14, we were unable to cross-check the measured acids concentration with a second instrument owing to the unavailability of Br-MION-CIMS. However, we conducted the same set of iodine oxoacid nucleation experiments at −10 °C during CLOUD 13, CLOUD 14 and CLOUD 15. The measured nucleation rates as a function of IA_*x*_ concentration across these campaigns were consistent, indicating that the SA calibration experiments performed for NO_3_-CIMS were reliable and accurate. The calibration factor used during the CLOUD 14 campaign was 4.8 × 10^10^ cm^−3^ cps cps^−1^, with uncertainty of 50–150% (ref. ^2^).

#### DMS

DMS concentration was measured during our experiments using two separate instruments: a proton-transfer reaction mass spectrometer 3 operating in hydronium chemical-ionization mode (H_3_O-PTR3-CIMS)^57^ and a proton-transfer reaction mass spectrometer using hydronium as the reagent ion (H_3_O-PTR-MS)^58^. Throughout all campaigns, the instruments were regularly calibrated with a standard gas mixture containing various volatile organic compounds to correct for potential drifts in transmission efficiency or variations owing to absolute humidity. The estimated uncertainty in DMS concentrations is approximately 30% (ref. ^59^). During CLOUD 15 at −30 °C, DMS concentration was measured with a FUSION PTR-TOF^60^ (IONICON Analytik GmbH). Quantification was based on the calibrated sensitivity to acetone, which was 3,600 cps ppbv^−1^. The DMS signal, recorded counts per second, was normalized by acetone sensitivity and corrected for the reaction rate constants, mass-dependent transmission efficiency and isotope abundance. However, because DMS serves as the precursor to SA and MSA, both of which are directly measured, the concentration of DMS is not crucial to the results reported in this study.

#### NH_3_

Real-time measurements of gas-phase ammonia concentrations is challenging, especially at low concentrations owing to its high water solubility and strong surface affinity. In this study, NH_3_ concentrations were either directly measured or estimated from the injection rate. During the CLOUD 13 campaign, NH_3_ was measured using a protonated water cluster CIMS (CI-APi-TOF), which achieved a background level of approximately 4 pptv at 278 K and 80% RH. Detailed descriptions of the measurement set-up at CERN, including calibration and quantification procedures, are described elsewhere^61^.

During the CLOUD 14 and CLOUD 15 campaigns, NH_3_ was measured using the H_3_O-PTR3-CIMS, which is not optimized for NH_3_ measurements. The NH_3_ concentrations in the chamber at low pptv level were within the same range as the background signal of the instrument. The instrument background was also not stable but influenced by NH_3_ interactions with the internal surfaces, which depend not only on the surface materials used but also by changes in absolute humidity and temperature in the sample air. Specifically, ammonia can accumulate on the instrument surfaces and be released when humidity and temperature increase. In this study, we must also consider the stainless-steel chamber walls, which can store ammonia and subsequently release it at high temperatures. By contrast, under very cold and dry conditions, the chamber walls act almost as a perfect sink for NH_3_. Detailed information on instrument set-up, calibration and the effects of humidity and temperature is available elsewhere^62^. Given these measurement challenges and instrument limitations, the NH_3_ concentrations reported from H_3_O-PTR3-CIMS represent an upper limit of expected concentrations.

Furthermore, the H_3_O-PTR3-CIMS was unavailable during the CLOUD 15 −30 °C experiments, presented in this study. Therefore, for these experiments, the NH_3_ concentration in the chamber was estimated by numerically integrating a simple box model (gas and wall) with 1-s time steps. The model accounts for (1) continuous dilution owing to chamber flow, (2) reversible uptake to and re-evaporation from the chamber walls and (3) the NH_3_ injection rate (ppt s^−1^). The two equations solved are:

1 $$\frac{{{\rm{d}}C}_{(t)}}{{\rm{d}}t}={S}_{(t)}-{\lambda }_{(t)}{C}_{(t)}-{k}_{{\rm{on}}}{C}_{(t)}+{k}_{{\rm{off}}}$$

2 $$\frac{{\rm{d}}W}{{\rm{d}}t}={k}_{{\rm{on}}}{C}_{(t)}-{k}_{{\rm{off}}}$$

in which *C*_(*t*)_ denotes the gas-phase NH_3_ mixing ratio (pptv), *S*_(*t*)_ is the NH_3_ injection rate calculated from the MFCs (ppt s^−1^), ${\lambda }_{(t)}=\frac{Q}{V}\,=$ $\frac{\text{Chamber flow}}{\text{Chamber volume}}$ is the dilution rate (s^−1^) and *k*_on_ and *k*_off_ are the effective gas to wall uptake and wall to gas release rates, respectively (s^−1^). These values were estimated by fitting NH_3_ decay at −30 °C when NH_3_ measurements were present. The fit was performed using a weighted least-squares approach that accounted for the 30% measurement uncertainty of the H_3_O-PTR3-CIMS signal, yielding best-fit values and confidence intervals for the *k*_on_ and *k*_off_ rate constants. These uncertainties combined with a 10% uncertainty in the MFC-derived injection rate were propagated through the model using Monte Carlo analysis to obtain confidence intervals for predicted ammonia concentrations.

The H_3_O-PTR3-CIMS was also not available during CLOUD 16. However, the CLOUD 16 experiments were conducted under low-NH_3_ conditions, with no NH_3_ injected into the chamber in the preceding months. The resulting background NH_3_ concentration remained below 4 pptv, consistent with previous characterizations of the chamber contaminant level^61^.

#### Charged clusters

Naturally charged clusters were measured using two APi-TOF mass spectrometers from Aerodyne Research Inc., both operating in negative ion mode. The first instrument, a standard APi-TOF^63^, was used in the CLOUD 13, CLOUD 14 and CLOUD 15 campaigns. It was connected to the chamber by means of a standard stainless-steel inlet. During the CLOUD 15 campaign, the APi-TOF mode^51^ of the Br-MION2-CIMS was also used to measure negatively charged clusters by deactivating the inlet voltages responsible for directing the charged reagent ions into sample flow. The MION2-APi-TOF provided higher sensitivity compared with the standard APi-TOF. Consequently, the charged cluster data for CLOUD 15 were mainly obtained from the MION2-APi-TOF, with the standard APi-TOF serving as a validation instrument.

#### Particle-phase measurements

To analyse the chemical composition of small particles, we used a FIGAERO coupled with a CIMS. This technique involves collecting particles on a polytetrafluoroethylene filter, followed by a controlled heating process to thermally desorb lower-volatility molecules back into the gas phase^64^. We confirmed complete desorption in our experiments by observing the return of all signals to baseline levels at the end of each heating cycle. Although FIGAERO-CIMS typically uses iodide (I^−^) chemical ionization for detecting low-volatility compounds, we used bromide (Br^−^) ionization during CLOUD 14, using the same instrument set-up, to differentiate between the chemical ionization reagent and iodine species within the CLOUD chamber. The collection times for particle samples varied across experiments: 15 min for CLOUD 15 and 30 min for both CLOUD 13 and CLOUD 14.

#### Trace gases

Trace gas concentrations were monitored using a Thermo Environmental Instruments TEI 49C chemiluminescence analyser for measuring O_3_ and a Thermo Fisher Scientific 42i-TLE pulsed fluorescence analyser for measuring SO_2_.

#### Relative humidity

A chilled dew-point mirror hygrometer (DewMaster, Edgetech Instruments) measured the dew point (above 0 °C) or frost point (below 0 °C) inside the CLOUD chamber. The measured dew or frost point was converted to the actual water vapour pressure using the saturation vapour pressure over liquid water or ice, respectively, calculated from the Goff–Gratch equations^65^. RH was then calculated at the chamber temperature either with respect to liquid water or with respect to ice, as specified.

#### Temperature

The chamber temperature was determined by averaging readings from six thermocouple sensors and one platinum resistance thermometer (PT100). These sensors are mounted at equal intervals along a horizontal line, positioned radially near the midplane of the chamber. The chamber temperature uniformity is within less than ±0.06 °C in the radial direction and ±0.1 °C in the vertical direction for temperatures ranging from −70 °C to 20 °C (ref. ^66^).

#### Particle number size distribution

Several instruments were used to measure the number size distribution of particles and ions across a range of about 1 to 1,000 nm.

The Airmodus A11 nano Condensation Nucleus Counter (nCNC) measured particle number size distributions between 1 and 3 nm (ref. ^67^). This system consists of a particle size magnifier (PSM; Airmodus A10) and a condensation particle counter (CPC; Airmodus A20). The PSM operated in a scanning saturator flow mode between 0.1 and 1.3 l min^−1^, with data processed using the step inversion method. To minimize particle losses in the sampling line, a core sampling inlet was used, increasing total flow from 2.5 to 7.5 l min^−1^, with 5 l min^−1^ directed to a bypass and 2.5 l min^−1^ sampled by the PSM. The data were further corrected for losses within the 12-cm-long, 6-mm outer diameter core sampling piece.

The Neutral cluster and Air Ion Spectrometer (NAIS) from Airel OÜ was used to determine the number size distribution of ions within a mobility diameter range of 0.8–42 nm, as well as the total aerosol particles (both naturally charged and neutral) in the range of about 2–42 nm (refs. ^68,69^). Like any other electrical mobility spectrometers, the NAIS classifies and detects ions based on their electrical mobility. The total flow rate of the instrument is 54 l min^−1^, with 20 l min^−1^ sampled from the CLOUD chamber. The remaining flow was supplied by the NAIS Integrated Dilution System from Airel. The number size distributions obtained from the NAIS were corrected for line losses, dilution and the ion transmission correction developed by Wagner et al.^70^, which is applicable to the NAIS model used in the CLOUD experiments. The NAIS was operated only in the ion mode measuring naturally charged particles during CLOUD 13 and CLOUD 14 whereas in CLOUD 15 and CLOUD 16, it was operated in both ion mode (measuring naturally charged particles) and total mode (measuring both naturally charged and neutral particles).

Two commercially available Scanning Mobility Particle Sizer (SMPS) spectrometers^71^ from TSI were used to measure particle size distributions in the range 6–1,000 nm. The first instrument, termed the nanoSMPS, is a model 3936 equipped with a 3085 Nano DMA and a 3750 CPC. The second instrument, referred to as the longSMPS, is a model 3983 that featured a 3082 electrostatic classifier, a 3081a DMA and a 3775 CPC for particle detection. Both systems were connected to the chamber by means of core-sampling inlets to minimize particle losses. The measured particle size distributions were then corrected for expected line losses.

During the CLOUD 13 campaign, a differential mobility analyser-train (DMA-train) was also available. The DMA-train measures the particle number size distribution within a mobility diameter range 1.8–8 nm (ref. ^72^). It consists of six DMAs operating in parallel, each connected to a CPC for particle detection. The DMA-train has an inlet flow rate of 11 l min^−1^. To increase the flow in the main sampling line and reduce diffusional sampling losses, an extra 9 l min^−1^ of air was drawn in through a core-sampling inlet. In this study, the DMA-train was used to cross-validate the growth rates determined from the NAIS (see below).

#### Determination of the particle nucleation rate (*J*_1.7_)

The particle nucleation rate reported here corresponds to the flux of nanoparticles that grow to a mobility diameter of 1.7 nm. This follows previous CLOUD studies, as most chemical systems are expected to have formed stable clusters by this size, at which evaporation is negligible under the studied conditions. The rate is derived from the time evolution of the particle concentration $({N}_{{d}_{{\rm{p}}}})$, under the assumption that new particle formation is the dominant source term^73^. Other potential sources, such as coagulation of smaller clusters or evaporation from larger particles, are typically negligible in new particle formation chamber experiments and are therefore neglected.

3 $$\frac{{{\rm{d}}N}_{{d}_{{\rm{p}}}}}{{\rm{d}}t}={\rm{production}}-{\rm{loss}}\approx {J}_{{d}_{{\rm{p}}}}-{\rm{loss}}$$

In chamber experiments, particle losses occur through three main mechanisms: (1) coagulation with equal-sized or larger particles; (2) deposition onto chamber walls; and (3) dilution resulting from chamber volume exchanges. *J*_1.7_ is therefore expressed as:

4 $${J}_{1.7}=\frac{{\rm{d}}{N}_{1.7}}{{\rm{d}}t}+{S}_{{\rm{c}}{\rm{o}}{\rm{a}}{\rm{g}}{\rm{u}}{\rm{l}}{\rm{a}}{\rm{t}}{\rm{i}}{\rm{o}}{\rm{n}}}+{S}_{\text{wall loss}}+{S}_{{\rm{d}}{\rm{i}}{\rm{l}}{\rm{u}}{\rm{t}}{\rm{i}}{\rm{o}}{\rm{n}}}$$

in which $\frac{{\rm{d}}{N}_{1.7}}{{\rm{d}}t}$ is the time derivative of the total particle concentration above 1.7 nm, *S*_coagulation_ is the particle loss owing to coagulation with equal or bigger sized particles, *S*_wall loss_ is the particle loss to the chamber wall and *S*_dilution_ is the particle loss owing to dilution, which depends on the chamber volume and the continuous replenishment flow into the chamber. Further details on calculating each term for the CLOUD chamber are provided in ref. ^74^. To calculate the coagulation sink, we used the combined particle size distribution from three instruments (NAIS, nanoSMPS and longSMPS), whereas the nCNC data were used to provide the number concentration of particles larger than 1.7 nm. The measured particle nucleation rates were compared with those expected for the SA-NH_3_ system using the parameterization in ref. ^28^ and with those expected for the IA_*x*_-SA-NH_3_ system based on ref. ^40^. In the latter case, *J*_1.7_ was estimated using the empirical relationship between the nucleation rate and the concentrations of SA, IA and IOA. Specifically, He et al.^40^ showed that log_10_(*J*) scales almost linearly with log_10_((HIO_3_ + H_2_SO_4_) × HIO_2_), such that the nucleation rate can be approximated as:

5 $$J={10}^{-12.54}{(({{\rm{HIO}}}_{3}+{{{\rm{H}}}_{2}{\rm{SO}}}_{4})\times {{\rm{HIO}}}_{2})}^{1.15}$$

#### Determination of the growth rate

The growth rates were calculated using the 50% appearance time method^43,74,75^ over two size intervals: 1.8–3.2 nm and 3.2–8.0 nm in mobility diameter, consistent with the approach in ref. ^43^. The main instrument used for growth rate determination was the NAIS. However, during the CLOUD 13 campaign, when the DMA-train was available, further growth rates were calculated using DMA-train data. The DMA-train growth rates represent the growth of the total particle population, whereas the NAIS provided growth rates for both negatively and positively charged particles (ions) during CLOUD 13, CLOUD 14 and CLOUD 15. Furthermore, during CLOUD 15 and CLOUD 16, the NAIS was also operated in total mode, enabling calculating the growth rate for the total particle population in the 3.2–8.0 nm range. As shown in Extended Data Table 1, the growth rates calculated from the two instruments demonstrate strong agreement, even when comparing total particle growth rates to those of naturally charged particles. We also compared growth rates between the DMA-train and the NAIS in total mode for experiments with α-pinene and SA, confirming that growth rates measured by the NAIS in total mode closely align with those measured by the DMA-train.

The appearance time method can overestimate growth rates when coagulation growth is pronounced relative to condensational growth^76^. To evaluate this, we calculated the coagulation growth rate using the equations in ref. ^76^ for selected runs with GR_3.2–8.0_ values ranging from 1.79 to 26.0 nm h^−1^. The results showed that coagulation growth contributed at most 0.01% to the growth rate derived from the appearance time method. Consequently, coagulation growth is negligible in our experiments and can be disregarded.

### Atmospheric observations

Global observations of SA, MSA and IA from ground-based stations and shipborne expeditions, as shown in Fig. 5, were compiled from the literature. To facilitate comparison across sites, all data were averaged to hourly resolution. The following locations and sources were included: Villum (81.6023° N, −16.6620° E, 24 m above sea level (a.s.l.)) and Ny-Ålesund (78.9255° N, 11.9323° E, 45 m a.s.l.) from ref. ^21^; the MOSAiC expedition in the central Arctic Ocean from ref. ^77^; Värriö (67.7481° N, 29.6102° E, 370 m a.s.l.) from ref. ^78^; Helsinki (60.2049° N, 24.9636° E, 25 m a.s.l.) from ref. ^79^; Réunion Island (−21.0800° N, 55.3830° E, 2,150 m a.s.l.) from ref. ^27^; the Southern Ocean expedition from ref. ^20^; Aboa (−73.0500° N, −13.4167° E, 480 m a.s.l.) from ref. ^23^; Marambio (−64.2411° N, −56.6267° E, 198 m a.s.l.) from ref. ^24^; Chacaltaya (−16.3498° N, −68.1307° E, 5,240 m a.s.l.) from ref. ^59^; Utqiaġvik (formerly Barrow) (71.3248° N, −156.6739° E, 3 m a.s.l.) from ref. ^80^; and CAPRICORN2 expedition from ref. ^25^.

The aircraft measurements presented in Extended Data Fig. 6 were retrieved from several open-access repositories, in which ACE-1 stands for the First Aerosol Characterization Experiment^81^, PEM-Tropics B stands for the Pacific Exploratory Missions^82^, TRACE-P stands for the NASA Transport and Chemical Evolution over the Pacific^83^, INTEX-B stands for the Intercontinental Chemical Transport Experiment – Phase B^84^, PASE stands for the Pacific Atmospheric Sulfur Experiment^85^, ARCTAS stands for the Arctic Research of the Composition of the Troposphere from Aircraft and Satellites^86^ and SAS stands for Southeast Atmosphere Study^87^.

The sampling periods as well as corresponding temperature and RH conditions of all atmospheric observations presented in Fig. 5 and Extended Data Fig. 6 are summarized in Extended Data Table 2.

### EMAC model simulation

Global simulations were performed using the EMAC model, a numerical chemistry and climate simulation system that includes submodels describing tropospheric and middle atmosphere processes and their interaction with oceans, land and human influences^88^. The core atmospheric model is the fifth-generation European Centre Hamburg general circulation model (ECHAM5 (ref. ^89^)), nudged using Newtonian relaxation towards ECMWF ERA5 reanalysis over the years 2016–2019. For the present study, we applied EMAC with a spherical truncation of T63 (corresponding to a quadratic Gaussian grid of approximately 1.88° at the equator) and 31 vertical hybrid pressure levels up to 10 hPa (about 32 km). The applied model set-up consisted of the submodels GMXe for aerosol microphysics using seven log-normal modes^90^, including: (1) treatment of multi-phase processes on deliquescent aerosols within the GMXe sub-submodel AERCHEM^91^; (2) NAN for the nucleation mechanisms^92^; (3) IONS for ion pair production rates from GCRs and radon decay^92^; (4) AEROPT for aerosol optical properties^93^; (5) MECCA for gas-phase chemistry^94^, using detailed DMS oxidation mechanism with extended halogen DMS reactions from the literature^2^; (6) JVAL for photochemistry^95^; (7) SCAV for cloud droplet multi-phase chemistry and the wet deposition of gases and aerosols^96^; (8) DRY-DEP for dry deposition^97^; (9) SEDI for aerosol sedimentation^97^; and (10) AIRSEA for emissions over the ocean^98^, following surface DMS climatology^14^. SA binary and ternary new particle formation is included^28^.

For MSA nucleation, we use the parameterization based on the E-AIM thermodynamic model to determine the MSA volatility transition^5^, further requiring that the ambient NH_3_ concentration is greater than three times MSA to nucleate. Also, we restrict MSA nucleation to only happen at ambient temperatures below −10 °C. Under these conditions, the sum of MSA and SA concentrations available for nucleation are given to the ‘ternary’ (neutral and ion-induced) *J*_1.7_ nucleation channel described in ref. ^28^. If the conditions are not met, only SA concentrations are used to calculate the ‘ternary’ *J*_1.7_ rate.

Particle growth is processed by GMXe’s modal 2 moments aerosol scheme, which includes condensation and coagulation processes. Gasaerosol partitioning is treated in ISORROPIA II (ref. ^99^) for semi-volatile inorganic species, whereas the submodel ORACLE^100^ treats the partitioning of organic species using a volatility-based approach. Condensation of inorganic and organic vapours increases particle mass and size within modal size bins, whereas coagulation transfers mass to larger modes and reduces number concentration. Under cold conditions, MSA condensation is treated analogously to SA, following ref. ^101^, otherwise MSA is treated as semi-volatile inorganic species. Low-volatility vapours that do not condense onto existing particles are supplied to the NAN submodel, which computes nucleation rates to form new particles added to GMXe’s nucleation mode. Finally, AERCHEM affects aerosol composition by means of non-equilibrium aqueous phase chemistry, indirectly influencing particle growth by altering hygroscopicity and water uptake. This integrated approach enables realistic simulation of aerosol size distribution dynamics and growth^102,103^.

## Online content

Any methods, additional references, Nature Portfolio reporting summaries, source data, extended data, supplementary information, acknowledgements, peer review information; details of author contributions and competing interests; and statements of data and code availability are available at 10.1038/s41586-026-10810-2.

## Supplementary information

Peer Review File

## Figures and Tables

**Extended Data Table 1:**
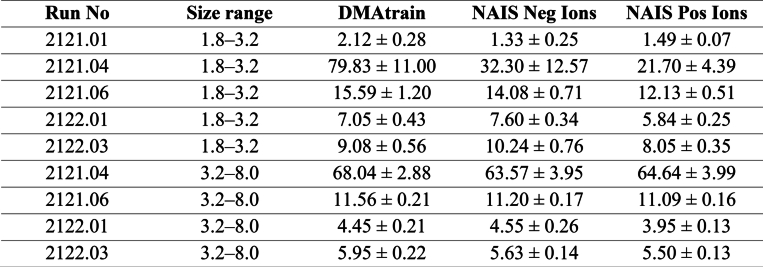
Comparison of growth rates measured using the DMA-train with those obtained from the NAIS in both negative and positive ion modes

**Extended Data Table 2:**
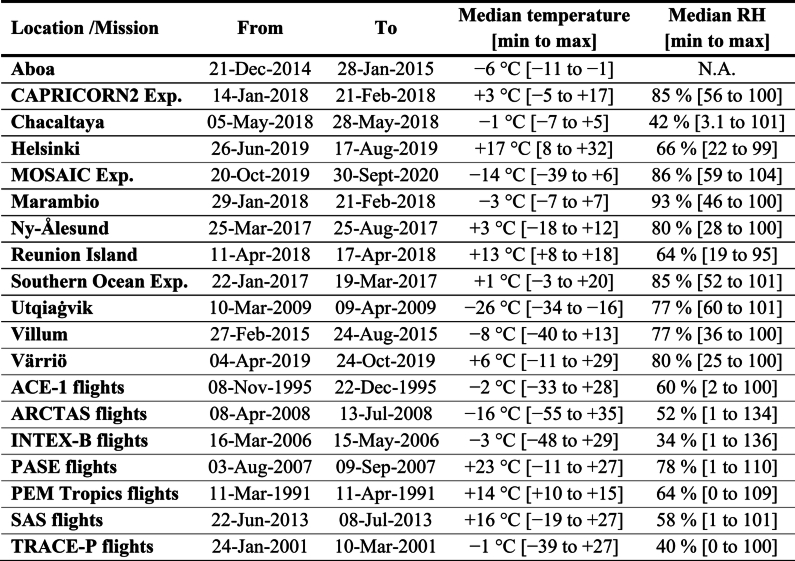
Sampling periods and corresponding temperature and RH conditions for the atmospheric observations in Fig. 5 and Extended Data Fig. 6

## Data Availability

The datasets underlying the experimental figures presented in this study are publicly available from Zenodo at 10.5281/zenodo.18997474 (ref. ^104^). Global atmospheric observations of sulfuric acid, methanesulfonic acid and iodic acid used in Fig. 5 are publicly available from the following repositories: Villum and Ny-Ålesund (10.5281/zenodo.4292239 (ref. ^105^)), MOSAiC expedition (https://doi.pangaea.de/10.1594/PANGAEA.963321 (ref. ^106^)), Värriö (10.5281/zenodo.5879549 (ref. ^107^)), Helsinki (10.5281/zenodo.6426198 (ref. ^108^)), Réunion Island (10.5281/zenodo.10302533 (ref. ^109^)), Southern Ocean expedition (10.5281/zenodo.2636771 (ref. ^110^); 10.5281/zenodo.3265832 (ref. ^111^); 10.5281/zenodo.5176217 (ref. ^112^)), Marambio (10.5281/zenodo.6560413 (ref. ^113^)), Chacaltaya (10.5281/zenodo.7429639 (ref. ^114^)), Utqiaġvik (10.5065/D6TM787P (ref. ^80^)) and CAPRICORN2 (10.25919/enc5-wh07 (ref. ^115^)). Aircraft measurements used in Extended Data Fig. 6 are publicly available from open-access repositories associated with the following campaigns: ACE-1 (10.26023/FRZK-NVH9-CT0S (ref. ^81^)), PEM-Tropics B (https://asdc.larc.nasa.gov/data/PEM/Tropics-B/TraceGas_AircraftInSitu_P3B_Data_1 (ref. ^82^)), TRACE-P (https://www-air.larc.nasa.gov/pub/TRACEP/P3B-AIRCRAFT/AIRCRAFT/EISELE.NCR/ (ref. ^83^)), INTEX-B – Phase B (https://www-air.larc.nasa.gov/cgi-bin/ArcView/intexb?C130=1#MAULDIN.LEE/ (ref. ^84^)), PASE (10.26023/P72H-HDDT-2Z0M (ref. ^85^)), ARCTAS (https://asdc.larc.nasa.gov/data/ARCTAS/TraceGas_AircraftInSitu_DC8_Data_1/ (ref. ^86^)) and SAS (10.26023/A58C-EF19-A80V).

## Code availability

The custom MATLAB and Python codes used for data analysis and figure generation in this study are available from the corresponding author on reasonable request.
