# Supplementary material for: Role of methanesulfonic acid in atmospheric particle nucleation and growth
Source: Nature. 2026 Jun 24;655(8125):1204–11. doi: 10.1038/s41586-026-10810-2 (PMC13421329; doi:10.1038/s41586-026-10810-2)
Supplement: Supplementary file 1 — Peer Review File [file 41586_2026_10810_MOESM1_ESM.pdf]

nature portfolio

Peer Review File

Role of methanesulfonic acid in atmospheric particle nucleation and growth

Corresponding Author: Dr Xu-cheng He

The original referee #1 and #2 were unable to review the revised version. The revised version was reviewed by the original referee #3 and additional referees #4 and #5

Any redactions in this file are there to maintain patient confidentiality, the confidentiality of unpublished data, or to remove third-party material.

This file contains all reviewer reports in order by version, followed by all author rebuttals in order by version.

Version 0:

Reviewer comments:

Referee #1

(Remarks to the Author)
This study investigated the role of methanesulfonic acid (MSA) in new particle formation and growth under different temperatures, in the presence of sulfuric acid (SA) and ammonia (NH3). Experiments conducted with MSA-SA-NH3 show that the nucleation rates at -30 oC and -10 oC are enhanced in the presence of MSA, by more than 2 orders of magnitude and tenfold, respectively, and scaling with MSA/SA ratio. The corresponding increase is weaker at 9 oC. At -10 oC, MSA is observed in the initial nucleating clusters with the dominating nucleation pathway involving multi-acid MSA-SA-NH3 clusters. It is found that MSA does not drive nucleation at 9 oC, though it contributes to particle growth across the temperature range of -52 oC to 9 oC. Experiments were also performed with iodine oxoacids (IAx), in which the presence of MSA increases the nucleation rate by more than an order of magnitude compared to SA-NH3-IAx alone. Overall, these results highlight the potential contributions of MSA to new particle formation and particle growth in cold pristine marine regions.

This is an interesting study and provides new insights into how MSA impacts new particle formation and growth at different temperatures. The experiments are well conducted. However, several aspects weaken the manuscript and reduce the novelty of this work. This is not the first study to investigate the role of MSA in atmospheric particle formation. Several prior studies are mentioned in the introduction (e.g., references 47-50), however, it is simply noted that those experiments were conducted above 20 oC and at MSA concentrations above 108 cm-3 (Though Johnson and Jen, ACS ESC, 2023, noted that their experiments were conducted with MSA concentrations ranging from 107 to 1010 cm-3) Chen et al. (J Phys Chem B, 2015) demonstrated the enhanced J>2.5nm in MSA-SA-NH3 systems. While these prior studies were conducted at higher temperatures and MSA concentrations, there is no discussion regarding how the results of this work should be interpreted in the context of prior findings, with respect to our understanding of the role of MSA in new particle formation and growth. A more in-depth comparison and discussion will better highlight the novelty of the present study. (In general, majority of the manuscript focuses on describing and discussing results from the current study only, without placing them in a broader context).

The chemical composition measurements at different temperatures are valuable in illustrating how MSA participates in new particle formation and growth. It appears that experiments were conducted at several discrete temperatures only (Figure 1), leaving the reader uncertain about the system's behavior between – 10 oC and 9 oC. It would be useful to provide some mechanistic insight and understanding to explain the differing observations at these two temperatures. For instance, Chen et al. performed chemical quantum calculations to determine the stability, formation energies, and enthalpies of initial MSA clusters.

The extrapolation of the experimental results to the role of MSA in new particle formation in ambient environments falls short. Figure 5 shows the concentrations of SA, MSA, and IA measurements from various ground stations, ship expeditions, and high-altitude observatories. However, these measurements alone do not support that "MSA is a key biogenic precursor for

new particle formation in cold marine regions". In-depth analysis is needed to clearly demonstrate that MSA plays a key role in new particle formation in these ambient datasets.

Overall, this manuscript has the potential to be published, provided that all the comments are fully addressed. In particular, in light of prior studies on MSA-SA-NH3 systems, it is important that the unique atmospheric relevance of the findings presented in this study can be clearly demonstrated to support its suitability for publication in Nature.

Specific comments

1. The title of the manuscript should be revised to better reflect the specific focus of this study, as there are a number of prior studies investigating the role of MSA in atmospheric particle formation.
2. Abstract
a. Line 66. It is stated that "Moreover, at temperatures below 9 oC, MSA and SA nucleate synergistically by forming multi-acid molecular clusters together with NH3." While Figure 4 shows the growth rate vs. acid concentration across a wide range of temperatures, the nucleation rates are only presented for three discrete temperatures (Figure 1, -30 oC, -10 oC, and 9 oC). Thus, the above statement is not supported as the data only demonstrates that MSA and SA nucleate synergistically at -10 oC (and lower temperature), and that MSA does not drive nucleation at 9 oC, i.e., no information is provided for the intermediate temperature range between -10 oC and 9 oC.
b. Lines 74-76 (and Line 326) This conclusion is overreaching and not supported by analysis. The results here show that MSA can potentially be a major driver of biogenic new particle formation in cold pristine marine regions, but this work did not demonstrate that it is a major driver in such ambient environments. [For data from Ny-Alesund shown in Figure 4D, they only support the role of MSA in particle growth in polar environments, but not in the nucleation process]
3. Line 130. What is the base-to-acid ratio of the diamond data points?
4. Line 136. Were any experiments conducted with higher MSA/SA ratios at 9 oC? (just like those at -30 oC and -10 oC)? It appears that all experiments at 9 oC have relatively similar MSA/SA ratios (Figure 1), making it a little challenging to conclude that "such scaling was observed at +9 oC". Related to this, Lines 437-439, what is the highest MSA/SA ratio achieved?
5. Line 137. With respect to "a weaker role of MSA at warm temperatures", it would be important to provide some mechanistic insights into the drastically different roles of MSA at -10 oC (and lower) vs. 9 oC, such that one can better understand what to expect for the temperatures in between and the applicability of the results over a wide range of ambient temperatures and conditions. For instance, have theoretical calculations been conducted to investigate the stability of the clusters at different temperatures?
6. Line 182. Were any experiments conducted without NH3 (containment level) at -10 oC and -30 oC?
7. Line 187 and Figure 2 caption appear to be inconsistent. Line 187 noted that Figure 2 shows the molecular composition of negatively-charged clusters during MSA-SA-NH3 nucleation. This is mainly to show how the chemical composition differs at -10 oC vs. 9 oC. However, Figure 2 caption noted that iodic acid (IA) and iodous acid (IOA) are also presented in the -10 oC experiment in panel B? If the experimental conditions (chemical species) are different between panels A and B, how does one draw conclusions regarding the effect of temperature only?
8. Line 267. It would be useful to elaborate further on the "hidden pool" of growth materials. What are these "hidden" clusters potentially?
9. Line 516. As the H3O+-PTR3-CIMS was not available during the -30 oC experiments, shouldn't the uncertainties in NH3 measurements be larger in these experiments? (Figure 1, etc.)
10. It would be very helpful to include an experimental table, listing the experimental conditions of all experiments presented in the figures. As of now, some experimental conditions are listed in the figure captions. However, it is not clear if the experiments in one figure are the exact same experiments as those shown in other figures.
11. What is the role of RH in the observations (does RH impact the observations)? It seems that there is some variation in RH between different experiments, but its role is not explored and discussed. Chen et al. (J Phys Chem B, 2015) demonstrated a strong dependence of MSA-SA-NH3 new particle formation on water vapor concentration.
12. Figure 5. It would be useful to note the temperature and RH for these datasets.

Referee #2

(Remarks to the Author)
This paper presents novel and important new experimental evidence for the role of methyl sulfonic acid (MSA) in the formation of new particles in the atmosphere. The role of reduced sulfur in new particle formation has been known for several decades and this has been recognised as playing an essential role in repopulating the background atmosphere with new particles after loss via precipitation has occurred. Thus far, the main species identified as driving this cycle has been sulfuric acid, an oxidation product of di-methyl sulfide (DMS). This cycle has been speculated as being a very important climate feedback since warmer temperatures increase biological productivity and the increase in resulting emissions leads to greater particle formation rates and hence cloud droplet numbers, enhancing cloud brightness. This feedback, known as the CLAW hypothesis, has remained an important scientific challenge since it is the main particle generation mechanism over the ocean in the background, pre-industrial state. Hence ensuring it is well represented in global models is important. However, much remains uncertain.

What the paper demonstrates for the first time is that another oxidation product of DMS, MSA, can, at low temperatures, also play an important role in particle formation in marine environments. The role of MSA in new particle formation has been speculated previously and some experimental and theoretical work has indicated it may be playing a role but this has never been unequivocally demonstrated previously. The experiments, carried out in the globally unique CLOUD experimental facility, show that at temperatures below -10C, MSA and ammonia gases can drive a particle formation, or nucleation,

process that is competitive with that of the sulfuric acid-ammonia system previously considered in global models to drive marine particle formation. The paper also shows for the first time, that MSA can take part in synergistic multi-acid nucleation along with sulfuric acid at rates that are over an order of magnitude larger than the particle formation rates of sulfuric acid alone, since the total available vapour concentrations are elevated. Lastly, the paper shows that at temperatures up to 9C (the warmest temperatures presented in the work) MSA enhances particle growth at sizes above 1.8 nm at the kinetic limit and even when ammonia is limited. This latter finding reinforces the earlier work of Johnson and Jin (reference 47) but covers a wider range of temperatures. As the authors point out this is important since growing newly formed particles rapidly reduces losses by diffusion to larger particles and increases the fraction growing to sizes capable of acting as cloud condensation nuclei. Taken together, these findings are very important results that have wide importance across fields of global atmospheric and marine science, climate science and physical chemistry and would certainly be worthy of publication in Nature.

The experimental work is of extremely high quality, the methodology is clear and well described. The facility used and the instrumentation deployed have been published extensively elsewhere. Though they are state of the art and allow insight into the chemical drivers of systems such as this, the novelty is in the particular chemical system and its wide ranging importance to the global atmosphere. These are certainly the right experiments and methods to address the problem.

My main comments are on the discussion and atmospheric implications. The current focus of the discussion is on high latitude marine boundary layers. This is for obvious reasons since DMS oxidation increasingly favours the MSA channel over that of sulfuric acid at colder temperatures and the results show MSA nucleation becomes important at temperatures lower than -10C. However, the impact may be significantly less than the discussion section concludes (see below). On the other hand, the findings presented in the paper may have important ramifications in other regions such as the upper troposphere and the role of MSA in growing particle clusters formed by sulfuric acid-ammonia may be very important globally. These two important aspects are not considered in the same detail in the discussion yet may well be as, if not even more important. I have detailed some of this below. This is not to diminish the findings but rather it is important to explore the wider importance.

In the discussion section, the authors make a strong case for MSA being important in new particle formation at high latitudes. The case for this is based on their findings that MSA plays an important role in elevating nucleation rates when combined with sulfuric acid (and also iodine acids if available) along with the availability of a base at temperatures of -10C and below. They also show field observations of MSA from a number of coastal stations at high latitudes that show MSA exists at equivalent concentrations to sulfuric acid. Their conclusion is therefore that “Our measurements show that MSA is a key biogenic precursor for new particle formation in cold marine regions yet remains unaccounted in global climate models.”. However, this is less convincing than first appears. Marine biochemistry is strongly seasonal, with maximum emissions of DMS taking place in summer months when sea-ice recedes, biological activity is at its strongest and temperatures are warmer. Under such conditions, air temperatures over the ocean in the polar regions are typically much warmer than -10C, often closer to, or even above, +10C. Under these conditions the experimental work presented shows no role for MSA in nucleation alone, though clear evidence of effective contribution to growth of particles above 1.8 nm. Of course, temperatures at boundary layer top will be cooler, likely by 5 to 10C, even so it is not obvious that the cold temperatures and reduced sulfur source strengths are coincident in space and time to have widespread impact. It would have been really good to see some model predictions of the effect of including MSA in nucleation under the prevailing conditions and emissions, which would offer more evidence of the scale of the effects. At the very least the above needs careful discussion in the final section to highlight the need for further work to address the key questions. I also note that the data sets included in figure 5 all come from the co-authors yet there are many other data sets from other studies covering wider parts of the marine boundary layer across the globe that contextualise the global picture. These should be included or at least discussed.

The upper atmosphere may well be an important environment. There are some ground based observations at altitude presented (Scholz et al from Chacaltaya), though the air masses sampled are not of recently uplifted air. Nevertheless, it is worth considering these data since MSA is relatively low compared to SA suggesting less of a role. It is worth including an argument in the paper that there is a clear need to sample air masses of marine air that have recently been lifted to mid and high altitudes to examine ratios and abundances of MSA and SA in these environments. This is worth noting since there is a major question over whether MSA is important in these regions.

In the discussion, less emphasis is placed on the role of MSA in growing newly formed clusters at rates that exceed those observed in the sulfuric acid system alone. The authors observed this to happen at all temperatures and in the absence of any base in the experiments. Since the biggest loss of very small particles is diffusion to existing particle surfaces, faster growth leads to higher survival probabilities and hence the likelihood that more particles survive to act as cloud condensation nuclei. This may yet be of greater important over many geographical regions than its role in nucleation directly and a discussion of this would be very good to see.

As climate is warming, the pre-industrial background may well be different to the background of today since the MSA formation pathway is quite temperature dependent and increases at low temperature. This, combined with the enhanced role of MSA at colder temperatures and the greater expanse of sea ice may well mean that particle formation in the pre-industrial may need to be considered carefully and include MSA. The authors may want to include a short discussion on this.
Lastly, there is no discussion of the typical base concentrations at high latitudes. This would be good to see in the context of the importance of the processes being discussed in different parts of the atmosphere.

Minor comments
Lines 71-72: “At temperatures of 9 °C or lower and relative humidities (RH) above 40 %, MSA drives kinetic growth of

particles above 1.8 nm diameter. These rapid growth rates were measured……" in the systems considered, would this be true of the ambient environment? The preceding sentence offers no insight into whether the growth rates were fast or slow. It would be helpful to say how much the rate is enhanced. Possibly something like: By a factor of 1.5 at equal concentrations of MSA and SA or similar.

Lines 114-116: "In summary, while laboratory and observational studies indicate that MSA contributes to the growth of pre-existing particles, the questions of its contribution to particle nucleation and early growth under atmospheric conditions remain open." The Johnson and Jin reference (#47) conducted experiments on nucleating particles and their conclusion, as stated on line 111, is that MSA does enhance particle growth at small sizes under warm temperature conditions, so perhaps the above statement needs re-wording to reflect that earlier finding.

Lines 301-303: Whilst the plot undoubtedly shows that MSA and SA vapours do contribute to particle growth, it is less clear why these vapours are "responsible for the particle growth" since there is no closure between the FIGAERO observations and the particle mass change. How have some other, undetectable species that co-varies with MSA and SA been ruled out?

Lines 306-307: This statement rather misrepresents the geographical variations in the concentrations of the different precursors vapours. Iodine acids are widely seen in enhanced concentrations in coastal regions, however, are at much lower concentrations over the clean, open ocean. Since many of the sites in figure 5 are at coastal sites the state is true but doesn't reflect the spatial distribution observed. The cruise data in the figure show substantially lower iodine as does the Reunion site, which has very little near shore and lacks a continental shelf in the immediate ocean. This needs to be reflected somewhat better.

Line 331: this should read the "marine biosphere"

Line 332: Figure 5 does not show data from the upper troposphere, it would be good to include a discussion of concentrations in the upper troposphere based on published literature and examine potential impacts in more detail in these regions.

Line 332: As currently presented the colour of the triangles in figure 5 have no meaning. This could be used to better illustrate which plot is from which location or even better use different colours to represent different sites.

Line 661: Why does the MSA decrease by 50% during the course of the experiment (Ext data fig 2 panel D) but that of H2SO4 does not?

Referee #3

(Remarks to the Author)
General Comments:
Baalbaki et al. present new and convincing measurements of the role of MSA in particle nucleation. It is well known that MSA contributes to particle growth to CCN sizes, but direct evidence that MSA contributes to particle nucleation has been elusive. This paper provides clear experimental proof and should certainly be published. It is an editorial decision of whether this is sufficient for publication in a broad interest journal such as Nature.

I would expect for publication in Nature that the authors would show that this enhanced nucleation rate had an impact on radiative cooling or at least on CCN concentrations.

Specifically, I was waiting to read this in the last section of the paper. The authors could use the measurements of MSA/SA (shown in Figure 5) to determine what the expected enhancement in the nucleation rate would be in these locations. They could then partner with an aerosol process level modeling team to determine the impact of that enhancement in nucleation rates on CCN (at least and ideally on radiative cooling). That would certainly be a nature paper if there was a sizeable impact. While this paper is certainly a great advance and is publishable as is in a specialized journal, it is not clear what the impact of an increase in the nucleation rate is on CCN number (and hence climate) in these regions of the atmosphere.

Detailed / Specific Comments:

Line 57: While DMS is a very large natural source of sulfur, passive volcanic emissions have recently been shown to be larger (at least in the Arctic).Reference is Jongebloed et al. GRL 2023, https://doi.org/10.1029/2022GL102061

Line 87: I am not sure that references 15 and 16 support the addition of "and, especially". That implies that the nucleation rate is the key source of uncertainty in the impact of DMS on radiative cooling. I am not sure these references support that. I'd recommend removing "and, especially" unless there is a reference that states that nucleation rate is the limits our understanding. If that was the case, I'd actually recommend expanding on that point. It is not abundantly clear to me at this point in the paper what impact a doubling the nucleation rate in these regions would have on radiative cooling. It would be good to expand a bit on this point.

Line 95: Related to my comment on line 57, I'd add volcanic H2S/SO2 emissions.
Figure 1: I really struggled to understand the coloured bands in panels B and C. As I understand, those coloured bands are for SA only. It makes it really hard to understand panels B and C since the x-axis as labeled is not SA. I get that the caption

says all this, but I doubt the reader will understand this. I wonder if it would be better to remove the shaded regions from B and C? The point is clearly made in panel A when comparing the observed nucleation rate to SA. I think all readers will understand that the rate is faster than that supported by SA chemistry alone.

Line 182: I’m not sure the experiments support the statement that MSA does not contribute to nucleation in the absence of base vapors like NH3 even in the upper troposphere. Perhaps I am misunderstanding the experiment. Was nucleation not observed for MSA only expriments at -50C or was it that nucleation was not enhanced when MSA and SA were present in the chamber relative to SA alone? Is this still true at very high MSA/SA ratios that might be possible in the UT? I don’t think there are many measurements of MSA/SA at 10km in convective outflow to be able to constrain what this ratio looks like? At -50C DMS oxidation proceeds almost exclusively down the OH addition pathway and it is possible that there are locations in the UT that have high MSA/SA ratios (this would require low sources of SO2).

Figure 5: This figure is quite interesting, but I think some additional information or discussion is needed here. The violin plots cover an enormous dynamic range, so it is not clear that MSA/SA is similar in all locations. That is also not very intuitive given the wide range in altitude and temperatures. I think focusing on the MSA/SA ratio in these plots and discussing how that ratio connects back to the experiments would be of more interest.

Version 1:

Reviewer comments:

Referee #3

(Remarks to the Author)
I have reviewed the revised manuscript and appreciate the authors careful attention to the three reviewers comments. It is especially noteworthy that they were able to connect the laboratory measurements of MSA nucleation to global estimates of CCN number in the revision. I support publication of the revised MS.

Referee #4

(Remarks to the Author)
In my view, the authors have thoroughly and satisfactorily addressed nearly all of the points raised by Referee #1 in the previous review round. I commend the authors for their careful and detailed responses. I would, however, suggest clarification of the following points:

(1) Page 4, second paragraph: The authors conclude that MSA does not contribute to nucleation above 9 °C, partly based on the statement: “The discrepancy between the measured and predicted SA–NH_3 nucleation rates at +9 °C (Fig. 1a) is within the combined systematic uncertainties of the measurements and parametrisation.” While I agree with the authors’ overall conclusion based on all evidence presented in the paper, I suggest that, if feasible, the systematic uncertainties associated with both the measured and parameterized nucleation rates be included in the Fig. 1a. In addition, please clarify whether the discrepancy falls outside the combined systematic uncertainties at the other two temperatures, particularly at –10 °C.

(2) Lines 172–173: Please provide an appropriate reference for the parameterization of the nucleation rate for the IAx–SA–NH_3 system. Alternatively, the parameterization could be briefly described in the Methods section.

(3) Line 712: It appears that the last term in the equation should read “–k_off” rather than “+k_off.” I also suggest revising “dC/dt” in Line 711 to “dC_(t)/dt” for consistency in notation.

Referee #5

(Remarks to the Author)
Review of He et al (2026)

This is an interesting paper and I have concentrated my focus on the modelling of MSA's impact on CCN with EMAC. Broadly the results are reasonable and make the impact of the new nucleation mechanism clearer. However, I have a few comments which should be attended to before the manuscript is considered for publication.

1. The phrase "after incorporating our new results (see Methods for further details)" should be more specific given it is the main text. It should state concisely exactly which nucleation mechanism(s) have been added, provide the nucleation rate equation(s) and the growth rate mechanisms.

2. The response of CCN in Extended Figure 7 is sensible and the point about the role of MSA in polar regions supported. CCN at 0.4% supersaturation looks to increase by 60-70% in large regions of the Southern Ocean at 2 km altitude which is indeed an important result.

That said the comment:

“Our findings suggest that this bias may be largely eliminated by including the contribution of MSA, which is unaccounted in current climate models”

Should be more measured in my view given that we are not seeing a doubling of CCN (i.e. to resolve the 50% low bias referenced by the authors) and indeed, when considering an area-weighted perspective, most of the Southern Ocean sees increases in CCN of <60% at 4 km. It should be rephrased to the something like the following:

“Our findings suggest that inclusion of this new nucleation mechanism, which is not considered in current climate models, may play an important role in reducing the Southern Ocean CCN bias.”

3. I understand this is primarily an experimental paper but, to demonstrate a clear process chain in the modelling study, the ratio (new mechanism / control) of total nucleation rate and total N50 and/or N100 should be plotted alongside the CCN.

Open Access This Peer Review File is licensed under a Creative Commons Attribution 4.0 International License, which permits use, sharing, adaptation, distribution and reproduction in any medium or format, as long as you give appropriate credit to the original author(s) and the source, provide a link to the Creative Commons license, and indicate if changes were made.
In cases where reviewers are anonymous, credit should be given to 'Anonymous Referee' and the source.
The images or other third party material in this Peer Review File are included in the article’s Creative Commons license, unless indicated otherwise in a credit line to the material. If material is not included in the article’s Creative Commons license and your intended use is not permitted by statutory regulation or exceeds the permitted use, you will need to obtain permission directly from the copyright holder.
To view a copy of this license, visit https://creativecommons.org/licenses/by/4.0/

Nature manuscript 2025-07-18931

Title: "Role of methanesulfonic acid in atmospheric particle formation"

We thank the reviewers for their careful evaluations and constructive comments, which have led to important improvements in our manuscript. Below, we present our point-by-point responses to each issue raised by the reviewers. Reviewer comments are shown in black, our replies are in green, the original manuscript text is in orange, and the revised text is in blue. The revised text is also coloured in blue in the "revised manuscript" file. All line and figure numbers refer to those for the revised manuscript. References cited in our responses are listed at the end of this document.

Referee #1 (Remarks to the Author):

This study investigated the role of methanesulfonic acid (MSA) in new particle formation and growth under different temperatures, in the presence of sulfuric acid (SA) and ammonia (NH_3). Experiments conducted with MSA-SA-NH_3 show that the nucleation rates at -30 °C and -10 °C are enhanced in the presence of MSA, by more than 2 orders of magnitude and tenfold, respectively, and scaling with MSA/SA ratio. The corresponding increase is weaker at 9 °C. At -10 °C, MSA is observed in the initial nucleating clusters with the dominating nucleation pathway involving multi-acid MSA-SA-NH_3 clusters. It is found that MSA does not drive nucleation at 9 °C, though it contributes to particle growth across the temperature range of -52 °C to 9 °C. Experiments were also performed with iodine oxoacids (IAx), in which the presence of MSA increases the nucleation rate by more than an order of magnitude compared to SA-NH3-IAx alone. Overall, these results highlight the potential contributions of MSA to new particle formation and particle growth in cold pristine marine regions.

- <u>General comments</u>

1) This is an interesting study and provides new insights into how MSA impacts new particle formation and growth at different temperatures. The experiments are well conducted. However, several aspects weaken the manuscript and reduce the novelty of this work. This is not the first study to investigate the role of MSA in atmospheric particle formation. Several prior studies are mentioned in the introduction (e.g., references 47-50), however, it is simply noted that those experiments were conducted above 20 °C and at MSA concentrations above 10^8 cm^{-3} (Though Johnson and Jen, ACS ESC, 2023, noted that their experiments were conducted with MSA concentrations ranging from 10^7 to 10^{10} cm^{-3}) Chen et al. (J Phys Chem B, 2015) demonstrated the enhanced J>2.5nm in MSA-SA-NH3 systems. While these prior studies were conducted at higher temperatures and MSA concentrations, there is no discussion regarding how the results of this work should be interpreted in the context of prior findings, with respect to our understanding of the role of MSA in new particle formation and growth. A more in-depth comparison and discussion will better highlight the novelty of the present study. (In general, majority of the manuscript focuses on describing and discussing results from the current study only, without placing them in a broader context).

We agree with the reviewer that our previous manuscript did not place the advances represented by our work sufficiently clearly in the context of prior studies and knowledge. In brief, our study provides four key advances over previous work: (1) atmospherically-relevant precursor concentrations, (2) free-tropospheric temperature conditions, (3) relative humidity (RH) conditions representative of the marine boundary layer, and (4) quantitative aerosol nucleation and growth rates versus vapour concentrations, which allow climate models to incorporate MSA in aerosol and CCN formation processes.

First, as shown by our survey of global atmospheric MSA concentrations (a modified Fig. 5 and a new Extended Data Fig. 6) [1–18], ambient MSA levels typically fall within the 10^5–10^8 cm^{-3} range. The experiments in our study span these concentrations and therefore allow quantitative determination of aerosol nucleation and growth rates without extrapolation uncertainties — one of the unique strengths of the CLOUD experiment. We note that although the Johnson and Jen study [19] represents, to our best knowledge, the first, and so far only, flow-tube investigation conducted at low sub-ppbv MSA levels, the lowest MSA concentration used in their experiments was *10^8 cm^{-3}*, as confirmed by all figures and captions, rather than *10^7 cm^{-3}*, as stated in their Methods section. Additionally, SA concentrations were between 5×10^7 and 8.4×10^8 cm^{-3}, and NH_3 concentrations were between 236 and 364 pptv. In contrast, our study represents the only set of experiments reaching MSA concentrations as low as 2×10^6 cm^{-3}, SA concentrations down to 4×10^5 cm^{-3}, and NH_3 concentrations down to below 4 pptv. Other studies [20–40], such as Chen et al. [24] and Chen and Finlayson-Pitts [28] involved MSA concentrations above 10^{10} cm^{-3}, four orders of magnitude above typical atmospheric concentrations.

The second key advance of our work is to present experimental results at temperatures representative of the free troposphere and cool boundary layer. As the reviewer notes, our results reveal a distinct temperature-dependent behaviour of MSA for particle nucleation and growth. Specifically, we find that MSA contributes to particle nucleation at temperatures ≤ −10 °C in the presence of NH_3 whereas, at 9 ℃ and atmospheric concentrations, MSA contributes to particle growth but not nucleation. The experiments of Johnson and Jen (as well as other earlier studies) were carried out at warm temperatures of 25 ℃ and so did not uncover the temperature dependencies of MSA-driven particle nucleation and growth under cooler conditions and atmospheric concentrations.

The third key advance of our work is to use RH conditions (40–80%) that are representative of marine environments, where humidity is typically high (see Extended Data Table 2 in the response to Comment 16). As originally found by Finlayson-Pitts et al and discussed in detail in our response to Comment 16, RH strongly influences MSA-driven nucleation and MSA is expected to rapidly evaporate from particles under low RH conditions. Therefore, experiments conducted at low RH (for example, the Johnson and Jen study at 20% RH) offer only a partial view of MSA's nucleation capability and are not suitable for assessing its role under marine conditions.

The fourth key advance concerns quantitative measurements of MSA nucleation and growth rates. Although particle formation rates have been reported in some earlier studies, robust quantitative measurements versus precursor vapour concentrations have not yet been made due to the experimental limitations of flow-tube setups, with their high vapour concentrations and short reaction times. In contrast, our measurements provide quantitative nucleation rates at 1.7 nm and growth rates from 1.8 nm onward, enabling the SA–MSA–NH_3 nucleation to be parameterised and included in global climate modelling.

We have now expanded the discussion of earlier MSA aerosol nucleation studies in the Introduction and better highlighted the novelty of our study in comparison to earlier work.

Original text (lines 111–116):

Experimentally MSA has been shown to enhance SA particle formation [19] *and form particles with amines and* NH_3 [24,28,35]*. However, these experiments were conducted above 20 °C and at MSA concentrations above* 10^8 *cm*$^{-3}$*, which are well above median MSA atmospheric concentrations of around* 10^6 *cm*$^{-3}$*. In summary, while laboratory and observational studies indicate that MSA contributes to the growth of pre-existing particles, the questions of its contribution to particle nucleation and early growth under atmospheric conditions remain open.*

Revised text (lines 109–123):

Experimentally, MSA has been shown to form particles with amines and NH_3 *at high (ppbv) precursor concentrations, and warm temperatures* [24,28,35]*. These studies have also shown the importance of water for formation and growth of MSA particles. A more recent study* [19] *has measured SA-MSA and MSA-MSA dimers in flow tube experiments at 25 °C and 20 % RH with* 2×10^8 *cm*$^{-3}$ *MSA, (2–8)×*10^8 *cm*$^{-3}$ *SA and base vapours (ammonia and amines). However, only relatively small (20–30 %) increases, or even decreases, in particle counts were measured when injecting* 2.5×10^8 *cm*$^{-3}$ *MSA into SA-base vapour mixtures* [19]*.*

In summary, ambient observations have established that MSA is an important contributor to particle mass, and laboratory studies have shown both the importance of high RH conditions and also that mixed SA-MSA dimers and larger particles form in the presence of base vapours at warm temperatures, and acid concentrations exceeding typical atmospheric levels. However, MSA has so far remained unaccounted in global climate models since quantitative experimental measurements of particle nucleation and growth rates with MSA have not yet been reported at the cool temperatures and low vapour concentrations representative of the polar boundary layer and free troposphere.

We have further demonstrated that our parameterised SA-MSA-NH_3 nucleation and growth rate measurements can be directly embedded into a global model (EMAC), revealing new insights on the potential source of the aerosols and CCN in the Southern Ocean:

Revised text (lines 320–333):

To evaluate the potential impact of MSA on global new particle and CCN formation, we have carried out an initial study with the EMAC (ECHAM/MESSy Atmospheric Chemistry) Earth system model, which incorporates multicomponent parameterisations of atmospheric new particle formation from CLOUD experiments. For the present study, the baseline CCN concentrations consider only SA(-NH_3*), calculated using the Dunne et al. parameterisation* [41]*, together with all primary aerosol emissions. After incorporating our new results in the EMAC model (see Methods for further details), our simulations indicate that MSA enhances CCN concentrations especially strongly in the Arctic and Antarctic coastal regions, from the boundary layer up to at least 4 km altitude (Extended Data Fig. 7). These results are supported by recent observations of elevated gaseous MSA from aerosol evaporation during katabatic outflow from the Antarctic continent, which draws in upper tropospheric air from over the Southern Ocean* [42]*. Earth system models systematically underestimate CCN by more than 50 % over the Southern Ocean, producing a large positive radiation bias in this climatically-important region* [43,44]*. Our findings suggest that this bias may be largely eliminated by including the contribution of MSA, which is unaccounted in current climate models.*

2) The chemical composition measurements at different temperatures are valuable in illustrating how MSA participates in new particle formation and growth. It appears that experiments were conducted at several discrete temperatures only (Figure 1), leaving the reader uncertain about the system's behavior between – 10 ^{o}C and 9 ^{o}C. It would be useful to provide some mechanistic insight and understanding to explain the differing observations at these two temperatures. For instance, Chen et al. performed chemical quantum calculations to determine the stability, formation energies, and enthalpies of initial MSA clusters.

Indeed, our results reveal a striking shift in the role of MSA at +9 °C, where it contributes only to aerosol growth, compared with −10 °C, where it contributes to both aerosol nucleation and growth. Although scanning a finer temperature grid was not possible within the available experimental time, our molecular cluster measurements at +9°C suggest that the temperature threshold at which MSA begins to participate in nucleation lies not far below +9°C. In particular, at an MSA concentration of 1.04×10^{7} cm^{-3}, SA of 1.6×10^{7} cm^{-3}, and NH_3 of 123 pptv, MSA was detected in clusters containing five SA molecules (Fig. 2a). This shows that MSA can contribute to particle growth immediately after the nucleation barrier represented by the negatively charged SA tetramer (*m/z* = 400–450 Th) has been crossed. At similar MSA concentration but with threefold lower NH_3 (43 pptv), a marked reduction in MSA of the smallest (5 SA) clusters was observed (Extended Data Fig. 3). Such a strong response to a small decrease in NH_3 indicates that, for these concentrations at +9 °C, MSA is rapidly evaporating and highly sensitive to NH_3, even at 100:1 base:acid ratio. This sensitivity and the appearance of MSA in clusters immediately above the nucleation barrier represented by $(SA)_4.(NH_3)_x$ implies that the system is close to the temperature at which MSA can begin to nucleate under atmospheric conditions. A small reduction in temperature (see Fig. R1), together with high NH_3 concentrations, is likely to provide sufficient stability for to MSA to nucleate. However, the precise temperature at which this transition occurs remains to be measured in future experiments. These observations are now added to the revised text.

Revised text (lines 205–215):

Nevertheless, in the experiment shown in Fig. 2a, MSA was detected in clusters containing five SA, showing that MSA can contribute to particle growth immediately after the nucleation barrier represented by the negatively-charged SA tetramer (m/z = 400–450 Th in Fig. 2a) has been crossed. Further insights are provided in Extended Data Fig. 3, which compares the molecular composition of the experiment in Fig. 2a with another experiment at +9 °C with similar MSA concentration but three times lower NH_3 *concentration (123 pptv versus 43 pptv). The lower* NH_3 *produces a marked reduction of MSA in* $(SA)_5(NH_3)_n$ *clusters, indicating that MSA rapidly evaporating from the five SA clusters at +9 °C and is highly sensitive to* NH_3 *concentration, even at base-to-acid ratios of more than 100. We infer from these molecular measurements that the temperature at which MSA begins to nucleate under atmospheric conditions is not far below +9 °C, and requires the presence of relatively high* NH_3 *concentrations.*

Following the reviewer's suggestion, we have also complemented our observations with theoretical analyses to investigate the mechanism underlying this transition. Most published quantum chemical studies have focused on neutral or dry MSA-containing clusters, which are known to be unstable under atmospheric conditions [45], making it essential to examine hydrated clusters as they can be markedly more stable [46,47]. Using the reported thermodynamic parameters (ΔH and ΔS) from Chen et al. (2020) [47] and Li et al (2007) [46], we calculated the standard Gibbs free energies (ΔG) and corresponding evaporation rates for relevant hydrated clusters across −40 °C to +9 °C using the ACDC code [48]. Because the ΔG values reported in the literature vary considerably depending on the methodology used as shown in Table R1, and accepting that these differences are beyond the scope of this study, we normalised all evaporation rates to their corresponding values at +9 °C. This approach highlights the temperature dependence of the rates rather than their absolute magnitudes. Across the studied clusters (Fig. R1), the evaporation rates of hydrated MSA-NH_3 clusters decrease exponentially with decreasing temperature - for example, by factors of ~27 and ~34 for $(MSA)_1(NH_3)_1$ and $(MSA)_2(NH_3)_2$, respectively, between +9 °C and −10 °C. Even the more conservative data from Li et al. (2007) [46] show a reduction by nearly one order of magnitude. Such decreases in evaporation enhance individual cluster survival and can collectively increase nucleation rates by several orders of magnitude. This theoretical trend qualitatively mirrors our measurements, where MSA-driven nucleation becomes prominent at −10 °C. We did not opt for calculating the absolute theoretical nucleation rates because of the uncertainty linked to the chosen thermodynamic dataset (Table R1). Nevertheless, the consistent exponential decrease in evaporation rates with decreasing temperature provides a strong theoretical argument to support our observation that MSA-driven nucleation does not proceed at +9 ºC but does at −10 °C. Additionally, the quantum chemical studies also show that the most stable MSA-NH_3 clusters involve 1:1 acid:base ratio, as we measure in our study.

Table R1. Cluster ΔG_{ref} at 298.15 K and 1 atm

	Elm (2021)[45]	Chen et al (2016)[24]	Chen et al. (2020)[47]	Li et al. (2007)[46]	Wang et al. (2007) [49]	Oussoumanou et al. (2025) [50]
$(MSA)_1(NH_3)_1$	−3.4	−5	−5.37	−3.7		
$(MSA)_1(NH_3)_2$	−2.6		−7.04			
$(MSA)_2$	−5.4		−8.92			
$(MSA)_2(NH_3)_1$	−12.4		−18.29			
$(MSA)_2(NH_3)_2$	−20.5		−31.38			
$(MSA)_1(NH_3)_1(H_2O)_1$		−6	−8.79	−5.8		
$(MSA)_1(NH_3)_1(H_2O)_2$			−14.49	−8.9		
$(MSA)_1(NH_3)_1(H_2O)_3$			−18.93	−10.6		
$(MSA)_1(H_2O)_1$			−3.33		−1.41	−0.45
$(MSA)_1(H_2O)_2$			−4.81		−1.73	−1.15
$(MSA)_1(H_2O)_3$			−6.78		−1.55	−1.11

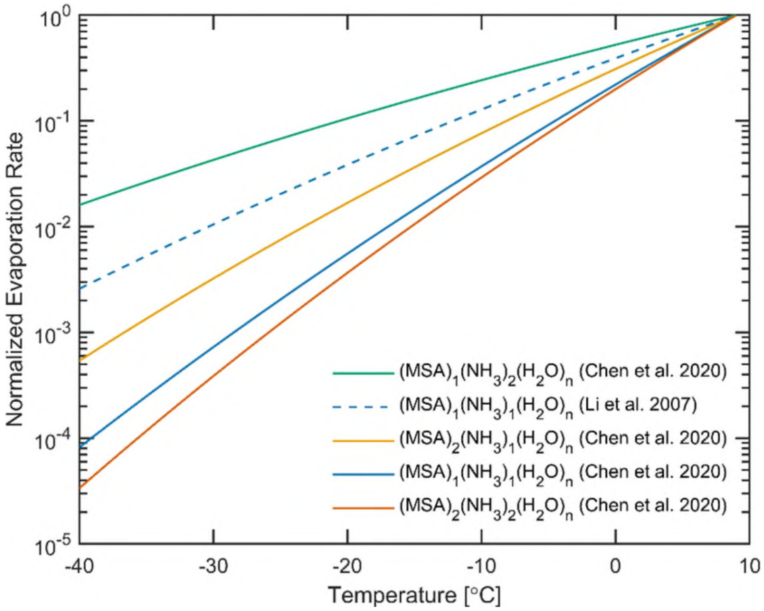

Figure R1. Temperature dependence of the total evaporation rates of MSA ($\Sigma\gamma$) for hydrated clusters normalised to the total evaporation rate at +9 °C for hydrated MSA-NH_3 clusters, where n corresponds to 1 to 3 water molecules.

3) The extrapolation of the experimental results to the role of MSA in new particle formation in ambient environments falls short. Figure 5 shows the concentrations of SA, MSA, and IA measurements from various ground stations, ship expeditions, and high-altitude observatories. However, these measurements alone do not support that "MSA is a key biogenic precursor for new particle formation in cold marine regions". In-depth analysis is needed to clearly demonstrate that MSA plays a key role in new particle formation in these ambient datasets.

We agree with the reviewer that this statement requires stronger justification. Briefly, we have now added Extended Data Fig. 6 (presented here under the answer to reviewer comment 18) to show global SA and MSA concentrations in the free troposphere from aircraft measurements. We have

also added a new lower panel in both that figure and Fig. 5 to show the *simultaneous* ratio of MSA to SA at these sites, which predominantly lies in a narrow range of around 0.7-1.3 i.e. MSA and SA concentrations in the remote marine atmosphere and free troposphere are comparable and tightly correlated. We have also carried out a global model study with EMAC to quantify the regional influence of MSA on CCN formation. We discuss this in more detail below.

Our study presents experimental measurements that show MSA contributes to early particle growth at temperatures below 9 °C and drives aerosol nucleation at temperatures below −10 °C. Ambient observations at the Ny-Ålesund station in the Arctic have shown that MSA contributes substantially to particle growth in springtime [11], although quantitative growth rates of sub 8 nm particles have remained unknown until our study. Because of instrumental limitations—particularly the absence of quantitative NH_3 measurements— we were unable to quantitatively determine the nucleation mechanism in Beck et al. (2021) [11], although the SA-NH_3 pathway was identified as the most plausible. By comparing the observed nucleation rates with concurrent vapour measurements, the authors inferred that NH_3 mixing ratios of several hundred pptv to above a few ppbv would be required to explain the total nucleation rates reported in Beck et al. (2021, Fig. S6A). Such concentrations are unlikely to be sustained in pristine Arctic environments, and other aerosol nucleation mechanisms such as SA-MSA-NH_3 mechanism could have played a role. Therefore, the overall conclusion from the Ny-Ålesund measurements is that MSA-driven particle early growth is clearly observed under cold conditions, whereas a contribution of MSA to particle nucleation cannot be excluded [11]. Because MSA-driven nucleation is highly sensitive to temperature, ambient MSA and NH_3 concentrations, and instrumental detection limits, long-term observations - including sensitive NH_3 measurements - are essential to quantify its role in the real atmosphere.

The available ambient observations (Fig. 5 and Extended Data Fig. 6) lack the comprehensive instrumentation or the spatial coverage to assess the global role of MSA-NH_3 in aerosol nucleation. Therefore, we employed the EMAC model to study the importance of MSA-NH_3 nucleation on a global scale, using conservative assumptions from our experimental measurements. In our simulations, we implemented the MSA volatility parameterisation of Hodshire et al. [51] which restricts the region where MSA contributes to particle growth to high relative humidities and cool temperatures. We further assume that MSA does not contribute to nucleation above −10 °C and that nucleation requires at least threefold higher NH_3 concentrations than MSA. Subject to these conditions, MSA is assumed to nucleate alongside SA, with comparable strength.

The observations presented in Fig. 5 are used to evaluate the model performance for realistically reproducing atmospheric SA and MSA. We note that modelling MSA is inherently challenging because global models typically overestimate gas-phase MSA concentrations [52,53]. In our simulations, despite updating the DMS oxidation mechanism (Shen et al. [54]; Fung et al. [55]) and implementing the most recent DMS and MeSH (Methanethiol) climatology [56] for the oceanic emissions, SA was overestimated by roughly a factor of 2.2 while MSA was overestimated by roughly a factor of 1.2 relative to atmospheric observations (Fig. R2). To avoid biases from these

errors in absolute concentrations, we show the *enhancement ratio* of the CCN concentrations for simulations that include MSA-driven nucleation relative to baseline simulations without MSA. The baseline CCN concentrations consider only SA-H_2O and SA-NH_3-H_2O nucleation together with all primary aerosol emissions, while those with MSA also include multi-acid MSA-SA-NH_3-H_2O nucleation.

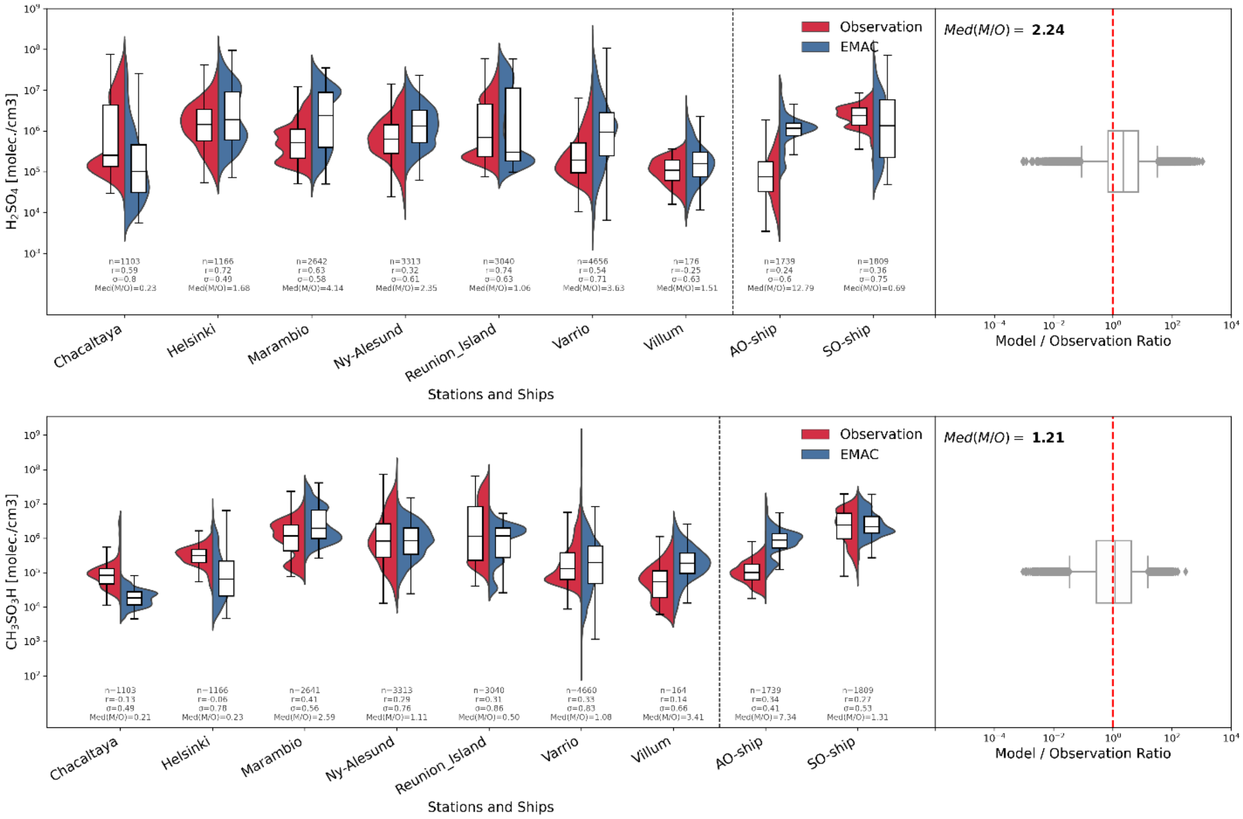

Figure R2. Modelled versus observed gas-phase SA (upper) and MSA (lower) concentrations at various locations where measurements are available. The left panels show gas-phase concentrations as split violin plots, where red indicates observations and blue represents EMAC simulations. Violin plots are overlaid with box–whisker elements showing the interquartile range (25th–75th percentiles; boxes) and the 5th–95th percentile bounds (whiskers). The Pearson correlation coefficient (r) and the standard deviation of the log difference (σ_{log}) between model and observations are given for each run while the number of data points in each observational dataset is indicated along the *x*-axis. The right panels display box–whisker plots of the model-to-observation ratios, with boxes showing the interquartile range and whiskers encompassing the full data range. AO-ship refers to the Arctic Ocean ship campaign[57] and SO-ship to the Southern Ocean ship campaign[10].

Our simulations reveal that MSA drives climatically-important increases in CCN at 0.4% supersaturation (equivalent to sizes of around 70–100 nm) across wide regions of the marine free troposphere and across the polar regions (Extended Data Fig. 7). The enhancement in the free troposphere is more pronounced over high southern latitudes than over high northern latitudes, reflecting reduced scavenging and a stronger vertical transport of precursors, driven by the katabatic outflow from the Antarctic continent. These simulations support our conclusion that MSA is a major contributor of aerosol and CCN in cool, pristine marine environments.

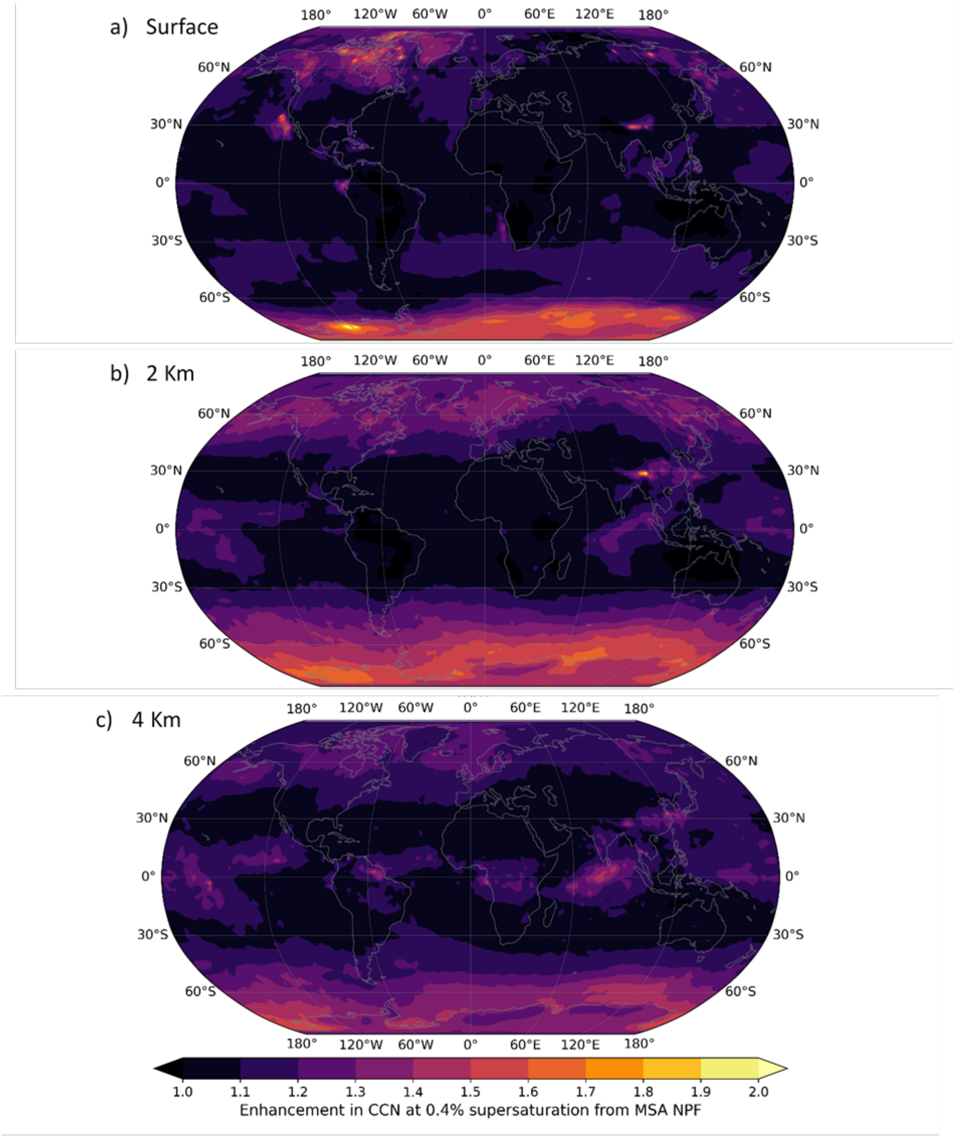

Extended Data Fig. 7. ***Yearly-averaged enhancement in CCN concentration at 0.4 % supersaturation due to MSA*** *Yearly-averaged enhancement in CCN concentration due to MSA as simulated by the EMAC model at a) the surface, b) 2 km altitude, and c) 4 km altitude, for the period 2016–2019. The baseline CCN concentrations consider only SA(-NH_3) particle formation, calculated according to the Dunne et al. parameterisation* [41]*, together with all primary aerosol emissions* [58]*. The enhancement ratio shows the CCN concentrations relative to the baseline, after including the SA-MSA-NH_3 nucleation and growth rates measured in the present study (see Methods for further details)*

Revised text in Methods section (Lines 856–891)**:**

Global simulations were performed using the EMAC model, a numerical chemistry and climate simulation system that includes submodels describing tropospheric and middle atmosphere processes and their interaction with oceans, land and human influences [59]*. The core atmospheric model is the 5th generation European Centre Hamburg general circulation model (ECHAM5* [60]*), nudged using Newtonian relaxation towards ECMWF ERA5 reanalysis over the years 2016–2019. For the present study we applied EMAC with a spherical truncation of T63 (corresponding to a*

quadratic Gaussian grid of approx. 1.88 degrees at the Equator) and 31 vertical hybrid pressure levels up to 10 hPa (~32 km). The applied model setup comprised the submodels GMXe for aerosol microphysics using seven log-normal modes [61]*, including treatment of multiphase processes on deliquescent aerosols within the GMXe sub-submodel AERCHEM* [62]*, (ii) NAN for the nucleation mechanisms* [63]*, (iii) IONS for ion pair production rates from galactic cosmic rays and radon decay* [63]*, (iv) AEROPT for aerosol optical properties* [64]*, (v) MECCA for gas phase chemistry* [65]*, using detailed DMS oxidation mechanism with extended halogen DMS reactions from literature* [66] *(vi) JVAL for photochemistry* [67]*, (vii) SCAV for cloud droplet multiphase chemistry and the wet deposition of gases and aerosols* [68]*, (viii) DRY-DEP for dry deposition* [69]*, (ix) SEDI for aerosol sedimentation* [69]*, (x) AIRSEA for emissions over the ocean* [70]*, following surface DMS climatology* [56]*. Sulfuric acid binary and ternary new particle formation is included* [41]*.*

For MSA nucleation, we use the parameterisation based on the E-AIM thermodynamic model to determine the MSA volatility transition [51]*, further requiring that the ambient NH_3 concentration is greater than three times MSA to nucleate. In addition, we restrict MSA nucleation only to happen at ambient temperatures below −10 °C. Under these conditions, the sum of MSA and SA concentrations available for nucleation are given to the "ternary" (neutral and ion-induced) $J_{1.7}$ nucleation channel described in Dunne et al. (2016)* [41]*. If the conditions are not met only SA concentrations are used to calculate the "ternary" $J_{1.7}$ rate.*

Particle growth is processed by GMXe's modal 2 moments aerosol scheme, which includes condensation and coagulation processes. Gas-aerosol partitioning is treated in ISORROPIA-II [71] *for semi-volatile inorganic species, while the submodel ORACLE* [72] *treats the partitioning of organic species using a volatility base approach. Condensation of inorganic and organic vapours increases particle mass and size within modal size bins, while coagulation transfers mass to larger modes and reduce number concentration. Under cold conditions MSA condenses as sulfuric acid condensation is described in Vignati et al. (2004)* [73]*, otherwise MSA is treated as semi-volatile inorganic species. Low-volatility vapours that do not condense onto existing particles are supplied to the NAN submodel, which computes nucleation rates to form new particles added to GMXe's nucleation mode. Finally, AERCHEM affects aerosol composition via non-equilibrium aqueous phase chemistry, indirectly influencing particle growth by altering hygroscopicity and water uptake. This integrated approach enables realistic simulation of aerosol size distribution dynamics and growth* [58,74]*.*

<u>Revised Main text (lines 320–333)</u>**:**

To evaluate the potential impact of MSA on global new particle and CCN formation, we have carried out an initial study with the EMAC (ECHAM/MESSy Atmospheric Chemistry) Earth system model, which incorporates multicomponent parameterisations of atmospheric new particle formation from CLOUD experiments. For the present study, the baseline CCN concentrations consider only SA(-NH_3), calculated using the Dunne et al. parameterisation [41]*, together with all primary aerosol emissions. After incorporating our new results in the EMAC model (see Methods*

for further details), our simulations indicate that MSA enhances CCN concentrations especially strongly in the Arctic and Antarctic coastal regions, from the boundary layer up to at least 4 km altitude (Extended Data Fig. 7). These results are supported by recent observations of elevated gaseous MSA from aerosol evaporation during katabatic outflow from the Antarctic continent, which draws in upper tropospheric air from over the Southern Ocean [42]. *Earth system models systematically underestimate CCN by more than 50 % over the Southern Ocean, producing a large positive radiation bias in this climatically-important region* [43,44]. *Our findings suggest that this bias may be largely eliminated by including the contribution of MSA, which is unaccounted in current climate models.*

Overall, this manuscript has the potential to be published, provided that all the comments are fully addressed. In particular, in light of prior studies on MSA-SA-NH_3 systems, it is important that the unique atmospheric relevance of the findings presented in this study can be clearly demonstrated to support its suitability for publication in Nature.

We thank the reviewer for their positive assessment of our manuscript. In response to Comment 1, we have clarified in detail the unique aspects of our study and updated the discussion to better place our findings within the context of previous work. Furthermore, in response to Comment 3. we have now carried out global model simulations based on our experimental measurements that demonstrate the importance of MSA-SA-NH_3 nucleation for CCN concentrations in the cool boundary layer and free troposphere.

- Specific Comments

4) The title of the manuscript should be revised to better reflect the specific focus of this study, as there are a number of prior studies investigating the role of MSA in atmospheric particle formation.

We agree with the reviewer that several previous studies have examined MSA aerosol formation. However, the strength of our work lies in addressing atmospherically relevant conditions—spanning temperature, humidity, and SA, MSA and NH_3 vapour concentrations, among others. Investigating MSA-driven aerosol nucleation under realistic and wide-ranging atmospheric conditions is therefore a central contribution of this study, as reflected by the original title "Role of methanesulfonic acid in atmospheric particle formation". Another important factor that distinguishes this study from earlier studies is that we quantitatively separate particle nucleation from early particle growth— the two elements that underlie particle formation at a given threshold size. Given Nature's 75-character limit for titles (including spaces), and upon the request from the reviewer, we have modified the title to "Role of methanesulfonic acid in atmospheric particle nucleation and growth" (74 characters).

5) a. Line 66. It is stated that "Moreover, at temperatures below 9 °C, MSA and SA nucleate synergistically by forming multi-acid molecular clusters together with NH3." While Figure 4 shows the growth rate vs. acid concentration across a wide range of temperatures, the nucleation rates are only presented for three discrete temperatures (Figure 1, -30 °C, -10 °C, and 9 °C). Thus, the above statement is not supported as the data only demonstrates that MSA and SA nucleate synergistically at -10 °C (and lower temperature), and that MSA does not drive nucleation at 9 °C, i.e., no information is provided for the intermediate temperature range between -10 °C and 9 °C.

We thank the reviewer for pointing out this error. The original statement has been corrected as follows:

Original text (lines 64–67):
… we find that MSA nucleates together with NH_3 at temperatures of −10 °C and below, at rates comparable to SA–NH_3 at similar concentrations. Moreover, at temperatures below 9 °C, MSA and SA nucleate synergistically by forming multi-acid molecular clusters together with NH_3.

Revised text (lines 64–67):
… we show that MSA nucleates together with ammonia (NH_3) below −10 °C at rates comparable to SA-NH_3. Moreover, MSA and SA can nucleate synergistically below −10 °C, forming multi-acid molecular clusters together with NH_3.

6) b. Lines 74-76 (and Line 326) This conclusion is overreaching and not supported by analysis. The results here show that MSA can potentially be a major driver of biogenic new particle formation in cold pristine marine regions, but this work did not demonstrate that it is a major driver in such ambient environments. [For data from Ny-Alesund shown in Figure 4D, they only support the role of MSA in particle growth in polar environments, but not in the nucleation process

We agree with the reviewer and have discussed our response and changes to the manuscript in Comment 3 above.

7) Line 130. What is the base-to-acid ratio of the diamond data points?

The base-to-acid ratio for the data points shown as diamonds was below unity. Upon closer examination, we found evidence that iodine oxoacid clusters were present in these experiments, likely leading to an artificial enhancement of the observed nucleation rates. As this contamination compromises the interpretation of nucleation in these specific experiments, we removed the two diamond points from nucleation rate data presented in Fig. 1. Similarly, one data point at +9 °C was found to contain IA concentrations of ~10^6 cm^{-3} and was therefore also excluded (updated Fig. 1 is presented under the response to comment 8). However, we retained these experiments in Fig. 4, as an IA concentration of ~10^6 cm^{-3} does not meaningfully affect the measured growth rates and, moreover, IA is explicitly included on the *x*-axis of Fig. 4.

To clarify which experiments contribute to each figure (as also requested in Comment 14 below) and their conditions, we have provided the full datasets in the Baalbaki et al. data folder: https://drive.google.com/drive/folders/1hIBQXM0h1LNgBjiOoZrJW7iVDeWE3eOV

These datasets will be uploaded to the Zenodo open repository upon publication. A summary table of the experimental conditions is also provided (Table R2) in our response to Comment 14.

8) Line 136. Were any experiments conducted with higher MSA/SA ratios at 9 ^{o}C? (just like those at -30 ^{o}C and -10 ^{o}C)? It appears that all experiments at 9 ^{o}C have relatively similar MSA/SA ratios (Figure 1), making it a little challenging to conclude that "such scaling was observed at +9 ^{o}C". Related to this, Lines 437-439, what is the highest MSA/SA ratio achieved?

We agree with the reviewer that the scaling of nucleation rates with MSA concentration is more evident at −10 °C and −30 °C, where the MSA/SA ratio is high, but less evident at +9 °C, where this ratio is lower. Accordingly, we have removed the sentence in the manuscript referring to this scaling effect.

Original text which is removed in the revised version (lines 135–137):
Figure 1B further demonstrates that, at −30 °C and −10 °C, the nucleation rates scale with MSA concentration, while such scaling was not observed at +9 °C, suggesting a weaker role of MSA at warm temperatures.

However, to address this comment directly, we have included some additional measurements that we made at +9 ^{o}C, precisely to achieve a wider range of MSA concentrations. These experiments necessitated direct injection of MSA vapour into the chamber from a temperature-controlled evaporator, together with SA produced by photolysis of sulfur dioxide in the chamber. Since they involved different experimental conditions than all the other experiments shown in Fig. 1 (for which MSA was produced by DMS oxidation), we have indicated these four experiments by hollow points in Fig. 1. A further reason why these four experiments were not included in Fig. 1 of the original manuscript is that they involve poorly-known base concentrations (<1 pptv contaminant dimethylamine, DMA, plus <4 pptv contaminant ammonia; Fig. R3). The contaminant DMA arose for a brief period following an intervention in the CLOUD chamber to replace a repaired component. Despite the uncertainty in their concentrations, the base vapours were sufficient to reach nucleation rates comparable to those for SA-NH_3 with around 50–100 pptv NH_3 at +9 ^{o}C. These four points confirm our direct molecular measurements (Fig. 2a) that MSA does not contribute to nucleation at +9 ^{o}C, as discussed in the accompanying revised text. Three of these points are shown in Fig. 3, and so we have added the time evolution of $J_{1.7}$ to that figure (the fourth point was made at higher SA and so is not shown in Fig. 3). We note that, although DMA strongly promotes nucleation with SA, its contribution to particle growth is negligible. For example, the expected contribution of DMA condensation to GR is only 0.26 nm.h^{-1} at 1.8 nm and 0.19 nm.h^{-1} at 3.2 nm.

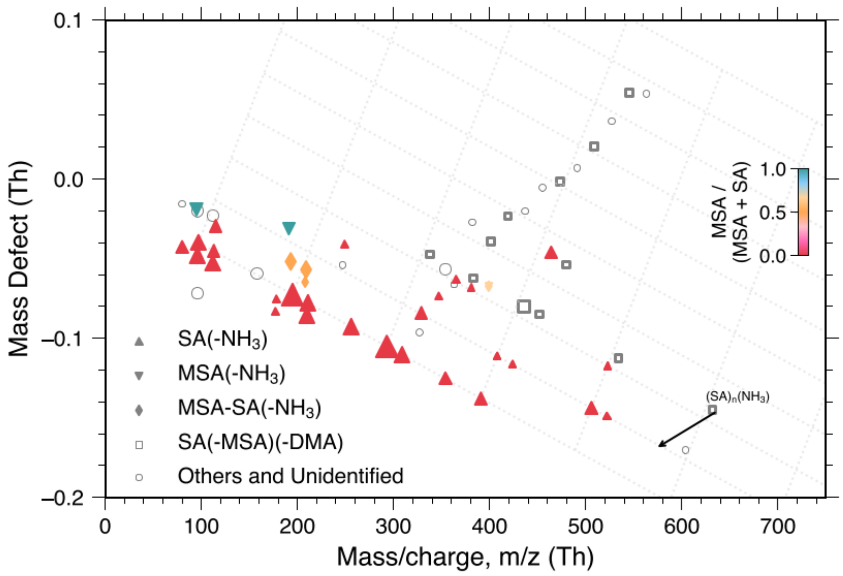

Figure R3. Molecular composition of negatively-charged clusters at +9 °C during run 2401.11 (also shown in manuscript Fig. 3j-l). The mass-defect plot indicates contaminant levels of both NH_3 and dimethylamine (DMA).

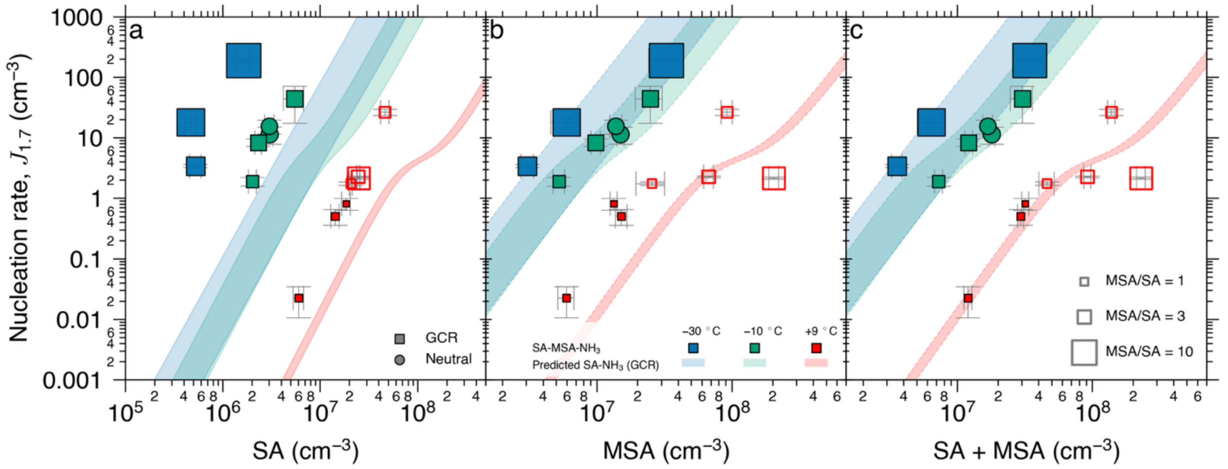

Revised Fig. 1 showing additional +9°C experiments at higher MSA-to-SA ratio (four hollow red points). The marker shape was also adjusted to clearly represent neutral (N) versus galactic cosmic rays (GCR) experiments.

<u>Revised text (lines 140–146):</u>

Experiments where SA and MSA were both generated from DMS are indicated by filled squares. To extend the MSA:SA range at +9 °C, some experiments were also carried out with direct injection of MSA vapour and with SA produced by oxidation of SO_2 (indicated by hollow red squares). The latter experiments include contaminant NH_3 (< 4 pptv) and contaminant dimethylamine (< 1 pptv), estimated from an Atmospheric Pressure interface Time-of-Flight (APi-TOF) mass spectrometer, which were sufficient to reach nucleation rates comparable to those for SA-NH_3 with 46–111 pptv NH_3 at +9 °C (as seen by the alignment of the filled and hollow red squares in Fig. 1a).

Revised text (lines 154–163):

Figures 1a and 1b show that the nucleation rates at −30 °C and −10 °C depend on MSA as well as SA, and align with those expected for SA-NH_3 when MSA is considered equivalent to SA (Fig. 1c). However, the behaviour is different at +9 °C; here the three hollow points at fixed 2×10^7 cm^{-3} SA show no change of nucleation rate after increasing the MSA concentration tenfold from 2.6×10^7 cm^{-3} to 2×10^8 cm^{-3} (Fig. 1b). In contrast, a doubling of the SA concentration under the same base vapour conditions increases the nucleation rate by one order of magnitude (Fig. 1a). The discrepancy between the measured and predicted SA-NH_3 nucleation rates at +9 °C (Fig. 1a) is within the combined systematic uncertainties of the measurements and parameterisation. We conclude that MSA at these concentrations is contributing to nucleation on an equal basis as SA below −10 °C but is not contributing to nucleation above +9 °C.

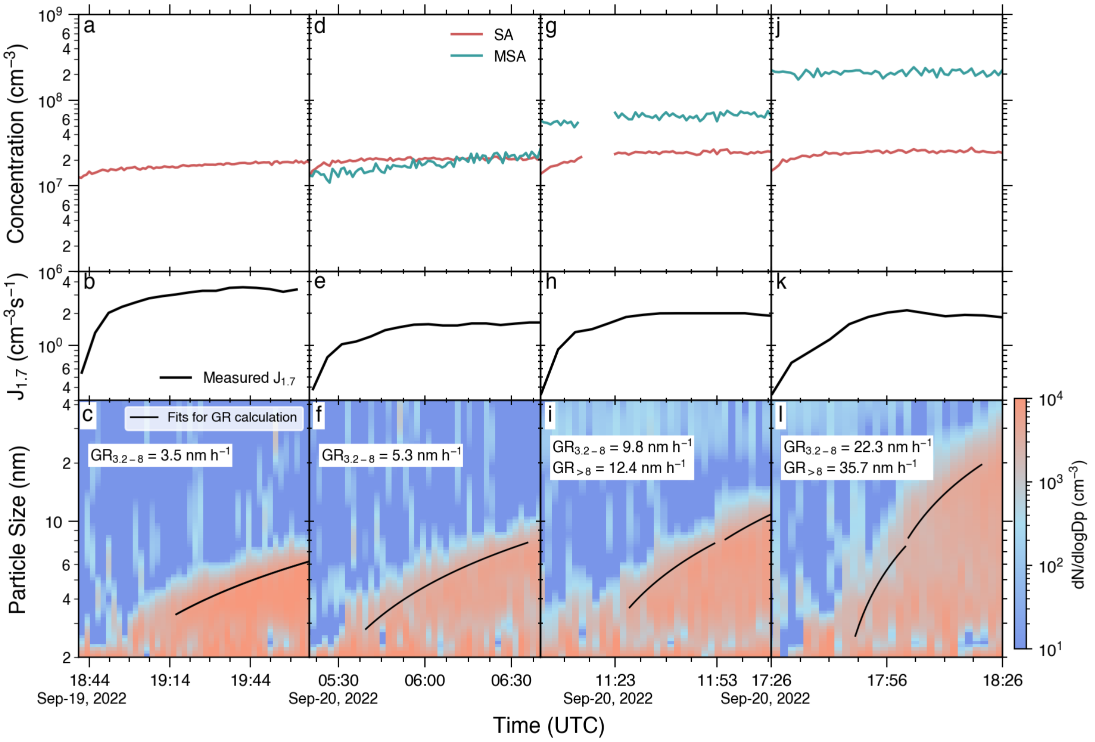

Revised Fig. 3 including middle panels showing the evolution of the nucleation rate measurements.

9) Line 137. With respect to “a weaker role of MSA at warm temperatures”, it would be important to provide some mechanistic insights into the drastically different roles of MSA at -10 °C (and lower) vs. 9 °C, such that one can better understand what to expect for the temperatures in between and the applicability of the results over a wide range of ambient temperatures and conditions. For instance, have theoretical calculations been conducted to investigate the stability of the clusters at different temperatures?

This point is addressed in our response to Comment 2 above.

10) Line 182. Were any experiments conducted without NH_3 (containment level) at -10 °C and -30 °C?

The manuscript does not include experiments without NH_3 at −30 °C. However, we have additional experimental data which confirms that MSA, at concentrations up to 1.4×10^7 cm^{-3}, does not nucleate at −30 °C in the absence of NH_3. Figure R4 shows that direct injection of 10^7 cm^{-3} MSA into the chamber at −30 °C produced no nucleation over more than three hours (between 15:00 and 18:00). When NH_3 was added, nucleation started immediately. Strong nucleation events were also observed after the NH_3 injection ceased, driven by contaminant residual ammonia remaining in the chamber.

We did not include this experiment in the manuscript because direct MSA injection at such low temperatures can cause localised supersaturation and potential nucleation near the injection port before the vapour becomes homogeneously mixed (a process taking ~2 min in the CLOUD chamber [75]). Nevertheless, this sensitivity test clearly demonstrates the essential role of NH_3 in MSA–NH_3 aerosol nucleation. As nucleation did not occur without NH_3 at −30 °C, it will also not occur at −10 °C, where MSA evaporation rates will be higher. We have now included the results from this experiment in the revised text:

Revised text (lines 186–190):

We also conducted a sensitivity test in which MSA was directly injected into the chamber at concentrations up to 1.4×10^7 cm^{-3} at −30 °C, in the absence of NH_3 and SA. No nucleation was observed until NH_3 was introduced to the chamber. These experiments show that, at concentrations below around 10^7 cm^{-3}, MSA does not contribute to particle nucleation in the atmosphere in the absence of a base vapour, even at temperatures as low as −52 °C.

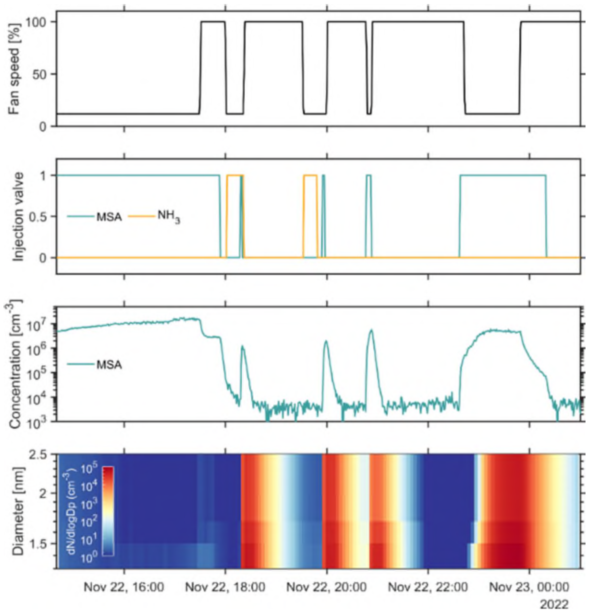

Figure R5. Experimental conditions during MSA injection at −30 °C before and after NH_3 addition. Shown are (top to bottom) fan speed, injection valve states for MSA and NH_3, MSA concentration, and particle size distribution measured by an nCNC. A fan speed of 12% corresponds to the standard setting, whereas 100% indicates cleaning stages.

11) Line 187 and Figure 2 caption appear to be inconsistent. Line 187 noted that Figure 2 shows the molecular composition of negatively-charged clusters during MSA-SA-NH_3 nucleation. This is mainly to show how the chemical composition differs at -10 ^oC vs. 9 ^oC. However, Figure 2 caption noted that iodic acid (IA) and iodous acid (IOA) are also presented in the -10 ^oC experiment in panel B? If the experimental conditions (chemical species) are different between panels A and B, how does one draw conclusions regarding the effect of temperature only?

Line 187 originally described Figure 2 as showing the molecular composition of negatively charged clusters during MSA–SA–NH_3 nucleation. However, the figure caption also indicated the presence of iodic acid (IA) and iodous acid (IOA) in the −10 °C experiment (panel B)—information that was not mentioned in the main text. We have now updated the text to explicitly note the presence of IA and IOA in these experiments.

However, it is important to note that, as seen from the MD plots in Figs. 2a and 2b, both experiments exhibited contaminant-level IA clusters (grey circles). IA concentrations in both experiments were ~10^6 cm^{-3}. Importantly, iodine-containing clusters were not the dominant nucleation pathway in either experiment, as their abundance was minor compared to MSA–SA–NH_3 clusters. Based on He et al.[76], with an IA to SA+MSA ratio of ~0.04, no enhancement of nucleation rate by iodine oxoacid is expected under the conditions of Figure 2b. Additionally, at IA concentration of 8×10^5 cm^{-3}, the ion-induced nucleation of IA would be too small to compete with MSA–SA–NH_3 ion-induced nucleation as quantitatively examined previously by He et al. [77]. Therefore, the comparison between −10 °C and 9 °C remains valid, even in the presence of the contaminant iodine-containing clusters.

We have added the following text to the manuscript to highlight the presence of IA and IOA in these experiments:

Revised text (Lines 195–197):

Contaminant iodine oxoacid was also present from previous measurements at concentrations around 10^6 cm^{-3} but represented a minor nucleation channel.

12) Line 267. It would be useful to elaborate further on the “hidden pool” of growth materials. What are these “hidden” clusters potentially?

The hidden pool comprises small acid-base clusters other than acid monomers that contribute to early particle growth through cluster-particle collision[78]. We addressed the potential ambiguity surrounding the term "hidden pool" by revising the sentence to explicitly describe these clusters and state their likely chemical composition as follows:

Original text (lines 266–268):

This enhanced growth results from collisions with molecular clusters, which represent a substantial, "hidden" pool of growth material that is unaccounted by the acid monomer measurements represented by the x-axis.

Revised text (lines 257–258):

This enhanced growth is due to the accretion of molecular acid-base clusters, which are unaccounted by the acid monomer concentration (x-axis).

13) Line 516. As the H3O+-PTR3-CIMS was not available during the -30 °C experiments, shouldn't the uncertainties in NH_3 measurements be larger in these experiments? (Figure 1, etc.)

The ammonia concentration estimated from the MFC settings for the −30 °C experiments has considerable uncertainty as the reviewer correctly points out. For a better representation of uncertainties, we used a more rigorous approach to estimate NH_3 concentrations that accounts for uncertainties in both injection rates and gas-wall interactions.

The ammonia concentration in the chamber depends on three factors: (1) the injection rate, (2) the dilution lifetime, and (3) gas-wall exchange. While the first two are known, gas-wall interactions were quantified by analysing decay data from a previous −30 °C experiment with PTR3-CIMS measurements. The ammonia decay following the stop of injection was fitted using a two-compartment (gas-wall) model that explicitly resolves reversible uptake and release from chamber walls. The fit was performed using a weighted least-squares approach that accounted for the 30 % measurement uncertainty of the PTR3-CIMS signal, yielding best-fit values and confidence intervals for the adsorption (k_{on}) and desorption (k_{off}) rate constants.

These fitted parameters, together with the ammonia injection rate derived from the MFC settings and the chamber dilution rate, were then used to predict the ammonia concentration in the −30 °C where PTR3-CIMS measurements were not available by numerically integrating the two-compartment mass balance equations. The uncertainties in k_{on} and k_{off} (from the fit) combined with a 10 % uncertainty in the MFC-derived injection rate were propagated through the model using Monte Carlo analysis to obtain confidence intervals for predicted ammonia concentrations.

As can be seen in Figure R5, NH_3 was injected in two pulses more than nine hours before the start of the −30 °C experiments (injection times indicated in the lower panel by vertical dashed black lines). During the subsequent equilibration period, the gas-phase ammonia concentration decayed to 52 pptv (95 % confidence interval: 0–121 ppt) by the beginning of the experiments, compared to 33 pptv (95 % confidence interval: 0–81 ppt) at the end of the experiments. We used the full range of the confidence intervals to estimate NH_3 concentrations, resulting in values between 0 and 121 pptv. However, because ammonia is prone to surface interactions, we set a more conservative lower limit of 4 pptv for these experiments.

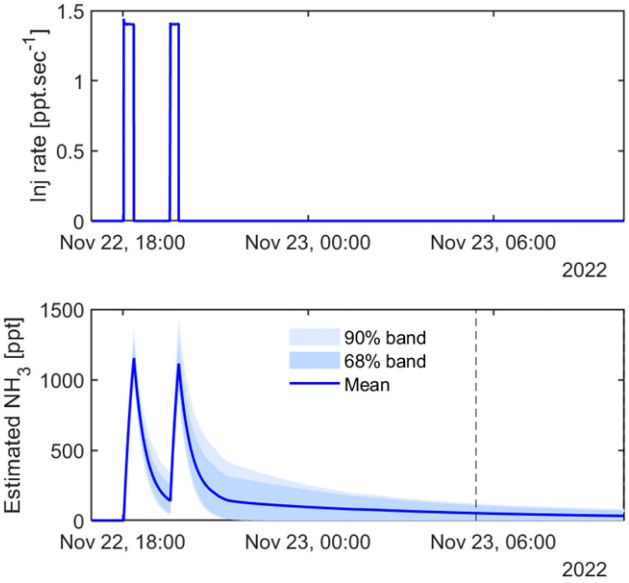

Figure R5. Modelled time evolution of ammonia (NH_3) in the −30 °C experiments. (Top) Ammonia injection rate into the chamber, showing two short pulses more than nine hours before the start of the nucleation experiments. (Bottom) Predicted gas-phase NH_3 concentration based on the two-compartment gas-wall model, including reversible wall uptake and release. The solid blue line represents the mean modelled concentration, while the shaded areas indicate the 68 % and 90 % confidence intervals obtained by Monte-Carlo propagation of the uncertainties and the ± 10 % uncertainty in the injection rate. The vertical dashed line marks the start of the −30 °C experiments presented in this study.

The following text was added to the Methods to explain the derivation of the NH_3 concentration at – 30 ^{o}C:

Revised text in methods section (lines 707–723):

Therefore, for these experiments, the NH_3 concentration in the chamber was estimated by numerically integrating a simple box model (gas and wall) with 1 second time steps. The model accounts for (i) continuous dilution due to chamber flow, (ii) reversible uptake to and re-evaporation from the chamber walls, and (iii) the NH_3 injection rate [ppt s^{-1}]. The two equations solved are:

$$\frac{dC}{dt} = S_{(t)} - \lambda_{(t)} C_{(t)} - k_{on} C_{(t)} + k_{off}$$

$$\frac{dW}{dt} = k_{on} C_{(t)} + k_{off}$$

Where

$C_{(t)}$ *denote the gas-phase NH_3 mixing ratio [pptv]*

$S_{(t)}$ *is the NH_3 injection rate calculated from the MFCs [ppt s^{-1}]*

$\lambda_{(t)} = \frac{Q}{V} = \frac{Chamber\ flow}{Chamber\ volume}$ *is the dilution rate [s^{-1}]*

k_{on} and k_{off} are the effective gas to wall uptake and wall to gas release rates, respectively [s^{-1}]. These values were estimated by fitting NH_3 decay at −30 °C when NH_3 measurements were present. The fit was performed using a weighted least-squares approach that accounted for the 30 % measurement uncertainty of the PTR3–CIMS signal, yielding best-fit values and confidence intervals for the k_{on} and k_{off} rate constants. These uncertainties combined with a 10 % uncertainty in the MFC-derived injection rate were propagated through the model using Monte Carlo analysis to obtain confidence intervals for predicted ammonia concentrations.

The following text was added to the caption of Figs. 1 and 4:

Revised text (line 523-524 and lines 562–563):
Ammonia was not measured during the −30 °C experiments but instead estimated using a box model (see Methods).

Additionally, the ammonia concentration quoted for Extended Data Fig 4 has been updated with the new estimated concentration range.

Revised text (line 936-937):
NH_3 was estimated from MFC settings to be between 4 and 119 ppbv (see methods for further details).

14) It would be very helpful to include an experimental table, listing the experimental conditions of all experiments presented in the figures. As of now, some experimental conditions are listed in the figure captions. However, it is not clear if the experiments in one figure are the exact same experiments as those shown in other figures.

We provide datasets for all figures in the Baalbaki et al. data folder: https://drive.google.com/drive/folders/1hIBQXM0h1LNgBjiOoZrJW7iVDeWE3eOV and in the Zenodo repository, which will be made publicly available upon publication. A summary table of the experiments is also included here (Table R2). It is important to note that not all experiments are equally suitable for deriving nucleation or growth rates. First, in some experiments the particles did not grow to 8 nm, preventing a reliable determination of growth rates. Second, in experiments with very rapid growth, the vapour condensation sink became exceptionally large, which made it difficult to accurately relate nucleation rates to vapour concentrations. For these reasons, only the highest-quality subsets of the data—where each quantity can be calculated with confidence—are included in our analysis.
It should be noted that the table lists median conditions over the full duration of each experiment, whereas the concentrations shown in the figures may differ because the averaging or calculation periods vary among them. For example, in Figure 1 (nucleation rates vs. acid concentrations), values are averaged over the period of steady-state nucleation, whereas in Figure 4 (particle growth rates), the averaging depends on the final particle size.

Table R2. Summary of experiments included in the study and their corresponding figures.

T	Run	Stage start	Stage end	Campaign	RH	MSA [cm^{-3}]	SA [cm^{-3}]	IA [cm^{-3}]	NH3 [pptv]	Figures
+9	2262.03	11/11/2019 19:33	11/11/2019 22:10	CLOUD14	36	7.0E+06	3.4E+06	<LOD	70	Fig. 1
+9	2262.06	12/11/2019 0:33	12/11/2019 2:53	CLOUD14	42	1.8E+07	1.3E+07	<LOD	50	Fig. 1, Ext. Fig. 3b
+9	2262.07	12/11/2019 2:53	12/11/2019 6:40	CLOUD14	42	1.7E+07	1.8E+07	<LOD	46	Fig. 1, Ext. Fig. 3b
+9	2262.09	12/11/2019 7:10	12/11/2019 10:31	CLOUD14	39	1.5E+07	1.6E+07	1.2E+06	111	Fig. 2a,4a-b, Ext. Fig. 3a
+9	2401.02	19/09/2022 18:39	19/09/2022 20:38	CLOUD15	63	<LOD	1.8E+07	<LOD	<4	Fig. 3 a-b
+9	2401.05	20/09/2022 5:12	20/09/2022 8:00	CLOUD15	62	2.4E+07	2.0E+07	<LOD	<4	Fig. 1,4a-b,4c-d, Ext. Fig. 5 a-b-c
+9	2401.07	20/09/2022 10:54	20/09/2022 13:14	CLOUD15	62	6.6E+07	2.4E+07	<LOD	<4	Fig. 1, 3e-f, 4a-b, 4c-d
+9	2401.11	20/09/2022 17:13	20/09/2022 19:19	CLOUD15	62	2.0E+08	2.3E+07	<LOD	<4	Fig. 1, 3 g-h, 4a-b
+9	2401.15	21/09/2022 9:03	21/09/2022 10:16	CLOUD15	62	5.7E+07	2.6E+07	<LOD	<4	Fig. 1, 4 a-b, 4c-d
−10	2119.03-2119.06	10/10/2018 21:48	11/10/2018 2:35	CLOUD13	62	2.5E+04	2.3E+06	2.5E+06	34	Ext. Fig. 1 a-b-c
−10	2121.01	12/10/2018 0:43	12/10/2018 5:26	CLOUD13	61	4.7E+06	3.7E+05	2.8E+06	67	Fig. 4 a-b,4c-d
−10	2121.02	12/10/2018 5:26	12/10/2018 8:40	CLOUD13	61	8.6E+06	5.2E+05	4.5E+06	77	Fig. 4 a-b
−10	2121.04	12/10/2018 12:05	12/10/2018 13:31	CLOUD13	58	7.5E+07	5.7E+06	2.5E+06	56	Fig. 4 a-b,4c-d
−10	2121.06	12/10/2018 19:08	12/10/2018 20:29	CLOUD13	58	2.5E+07	8.0E+06	1.7E+06	65	Fig. 4 a-b,4c-d, Ext. Fig. 1 d-e-f
−10	2122.01	12/10/2018 22:55	13/10/2018 1:07	CLOUD13	55	3.6E+06	1.6E+06	9.1E+06	82	Fig. 4 a-b,4c-d
−10	2122.03	13/10/2018 3:58	13/10/2018 6:02	CLOUD13	55	6.4E+06	2.1E+06	1.2E+07	91	Fig. 4 a-b,4c-d
−10	2123.01	13/10/2018 10:19	13/10/2018 10:41	CLOUD13	58	2.5E+07	1.5E+07	1.0E+06	74	Fig. 4 a-b,4c-d
−10	2123.02	13/10/2018 10:41	13/10/2018 11:53	CLOUD13	57	1.1E+07	8.5E+06	8.4E+05	71	Fig. 2b,4c-d
−10	2123.15-2123.18	14/10/2018 1:44	14/10/2018 3:30	CLOUD13	58	9.3E+06	6.7E+05	1.9E+06	88	Ext. Fig. 4a
−10	2263.01	12/11/2019 17:19	12/11/2019 21:46	CLOUD14	68	2.1E+07	4.6E+06	<LOD	239	Fig. 1,4 a-b,4c-d
−10	2263.03	12/11/2019 23:52	13/11/2019 3:29	CLOUD14	79	1.5E+07	3.0E+06	<LOD	127	Fig. 1,4c-d
−10	2263.04	13/11/2019 3:30	13/11/2019 5:01	CLOUD14	75	1.4E+07	3.0E+06	<LOD	222	Fig. 1
−10	2263.06	13/11/2019 7:15	13/11/2019 10:31	CLOUD14	71	7.5E+06	2.2E+06	<LOD	569	Fig. 1,4c-d

−10	2263.07	13/11/2019 10:31	13/11/2019 11:10	CLOUD14	72	5.2E+06	2.0E+06	<LOD	757	Fig. 1
−10	2263.01	12/11/2019 17:19	12/11/2019 21:46	CLOUD14	68	2.1E+07	4.6E+06	<LOD	239	Fig. 1,4c-d, Ext. Fig. 5 d-e-f
−10	2263.03	12/11/2019 23:52	13/11/2019 3:29	CLOUD14	79	1.5E+07	3.0E+06	<LOD	127	Fig. 1,4c-d
−10	2263.04	13/11/2019 3:30	13/11/2019 5:01	CLOUD14	75	1.4E+07	3.0E+06	<LOD	222	Fig. 1
−10	2263.06	13/11/2019 7:15	13/11/2019 10:31	CLOUD14	71	7.5E+06	2.2E+06	<LOD	569	Fig. 1
−10	2263.07	13/11/2019 10:31	13/11/2019 11:10	CLOUD14	72	5.2E+06	2.0E+06	<LOD	757	Fig. 1
−30	2468.17	23/11/2022 5:26	23/11/2022 6:38	CLOUD15	62	5.6E+06	4.6E+05	<LOD	48.8 [4-109]	Fig. 1,4 a-b,4c-d, Ext. Fig. 4b
−30	2468.19	23/11/2022 7:13	23/11/2022 8:28	CLOUD15	58	2.9E+06	4.6E+05	<LOD	40.7 [4-93]	Fig. 1,4 a-b
−30	2468.21	23/11/2022 9:40	23/11/2022 10:14	CLOUD15	61	2.7E+07	1.5E+06	<LOD	33.4 [4-79]	Fig. 1,4 a-b,4c-d
−10	2650.10	11/11/2023 5:35	11/11/2023 7:46	CLOUD16	59	3.1E+07	1.0E+07	1.6E+06	<4	Fig. 4 a-b,4c-d
−10	2650.13	11/11/2023 9:25	11/11/2023 12:05	CLOUD16	59	5.1E+07	2.0E+07	1.4E+06	<4	Fig. 4 a-b,4c-d
−50	2404.12	29/09/2022 6:11	29/09/2022 10:02	CLOUD15	70	<LOD	3.8E+06	<LOD	<1	Ext. Fig. 2 a-b-c
−50	2404.32	30/09/2022 18:41	30/09/2022 19:50	CLOUD15	70	3.7E+06	4.1E+06	<LOD	<1	Fig. 4 a-b,4c-d, Ext. Fig. 2 d-e-f

15) What is the role of RH in the observations (does RH impact the observations)? It seems that there is some variation in RH between different experiments, but its role is not explored and discussed. Chen et al. (J Phys Chem B, 2015) demonstrated a strong dependence of MSA-SA-NH3 new particle formation on water vapor concentration.

Indeed, RH can significantly influence MSA-driven new particle formation and growth. Several studies have demonstrated that water enhances MSA-related nucleation. For example, the Finlayson-Pitts group observed strong RH effects in both the MSA + trimethylamine[27] and MSA + amine/NH_3 systems[24], with Chen et al. (2016) [24] reporting a power-law dependence ($n \approx 2$) between water vapour and formation rate, implying that doubling RH increases *J* by roughly a factor of four. These findings, together with earlier flow-tube studies, show that hydration promotes MSA nucleation under dry-to-moderate RH conditions (< 60 %)[36–39].

TEXT REDACTED. However, it is important to note that most of the earlier work was comparing almost dry conditions to humid conditions, or else the experiments were conducted m ostly below 60 % RH.

Our present study focuses on marine environments, where RH is typically high (see Extended Data Table 2 in our reply to Comment 16). Within our high-RH range (40–80% in our experiments), we

observed no statistically-significant dependence of nucleation or growth rates on RH when we grouped all experimental data together. For the subset of MSA–SA–NH_3 experiments free of DMA contamination (Fig. 1), RH was nearly constant at each temperature, preventing a systematic evaluation of its influence on *J* (Figure R6). In terms of growth rate experiments, RH varied somewhat in the +9 °C and the −10 °C experiments (Figure R7), yet these experiments with RH spanning 40–60 % at +9 °C and 60–80 % −10 °C, showed comparable GRs (falling on the same line), suggesting a limited RH sensitivity under these conditions.

Mechanistically, RH affects NPF by stabilising small acid–base clusters through hydration and proton transfer, thereby lowering evaporation rates. This effect is strongest when clusters are weakly bound (e.g., low RH, low base availability) but diminishes once clusters are already stabilised by strong acids or bases or at collision-limited conditions [79]. Consequently, while RH can strongly influence MSA-driven NPF under low humidity conditions (below 40 % RH by our measurements), it has a weak effect at high relative humidity. Systematic studies of the RH effect on MSA nucleation and growth are certainly needed and will be the focus of future work by our group. This is especially important given that MSA can repartition from the particle-to-gas phase (at low RH or high acidity conditions), which allows for multiple contributions of MSA (recycling) to particle nucleation and growth [80].

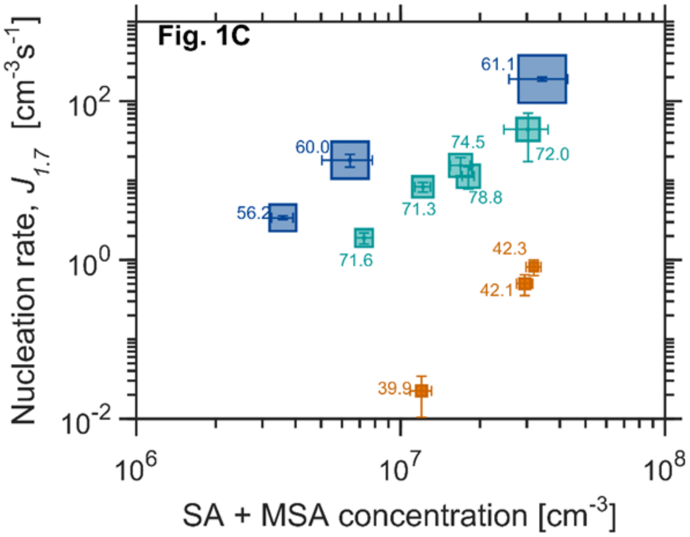

Figure R6. Adaptation of Fig. 1c showing nucleation rate versus total acid concentration for the DMS experiments, with the RH of each experiment indicated on the plot. The marker size indicates MSA/SA ratio

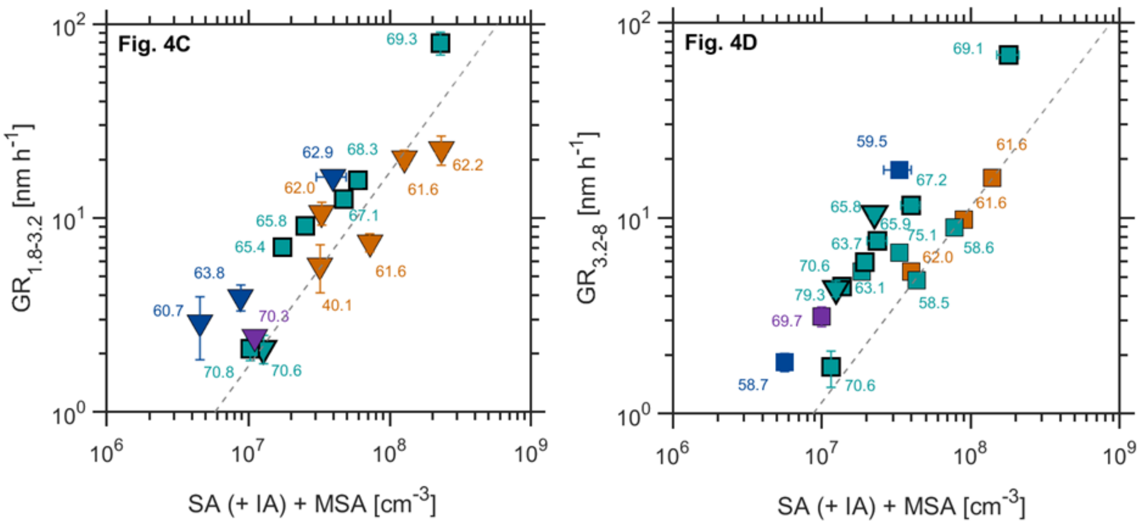

Figure R7. Adaptation of Fig. 4c and 4d showing growth rates versus total acid concentration, with the RH of each experiment indicated on the plot

16) Figure 5. It would be useful to note the temperature and RH for these datasets.

We have added the temperature and RH values for all datasets shown in the manuscript, including the additional ones requested by Reviewer 2, to Extended Data Table 2 in the manuscript.

Extended Data Table 2. *Sampling periods and corresponding temperature and relative humidity conditions for the atmospheric observations in Fig. 5 and Extended Data Fig. 6.*

Location /Mission	***From***	***To***	***Median temperature [min to max]***	***Median RH [min to max]***
Aboa	*21-Dec-2014*	*28-Jan-2015*	*−6 °C [−11 to −1]*	*N.A.*
CAPRICORN2 Exp.	*14-Jan-2018*	*21-Feb-2018*	*+3 °C [−5 to +17]*	*85 % [56 to 100]*
Chacaltaya	*05-May-2018*	*28-May-2018*	*−1 °C [−7 to +5]*	*42 % [3.1 to 101]*
Helsinki	*26-Jun-2019*	*17-Aug-2019*	*+17 °C [8 to +32]*	*66 % [22 to 99]*
MOSAIC Exp.	*20-Oct-2019*	*30-Sept-2020*	*−14 °C [−39 to +6]*	*86 % [59 to 104]*
Marambio	*29-Jan-2018*	*21-Feb-2018*	*−3 °C [−7 to +7]*	*93 % [46 to 100]*
Ny-Ålesund	*25-Mar-2017*	*25-Aug-2017*	*+3 °C [−18 to +12]*	*80 % [28 to 100]*
Reunion Island	*11-Apr-2018*	*17-Apr-2018*	*+13 °C [+8 to +18]*	*64 % [19 to 95]*
Southern Ocean Exp.	*22-Jan-2017*	*19-Mar-2017*	*+1 °C [−3 to +20]*	*85 % [52 to 101]*
Utqiaġvik	*10-Mar-2009*	*09-Apr-2009*	*−26 °C [−34 to −16]*	*77 % [60 to 101]*
Villum	*27-Feb-2015*	*24-Aug-2015*	*−8 °C [−40 to +13]*	*77 % [36 to 100]*
Värriö	*04-Apr-2019*	*24-Oct-2019*	*+6 °C [−11 to +29]*	*80 % [25 to 100]*
ACE-1 flights	*08-Nov-1995*	*22-Dec-1995*	*−2 °C [−33 to +28]*	*60 % [2 to 100]*
ARCTAS flights	*08-Apr-2008*	*13-Jul-2008*	*−16 °C [−55 to +35]*	*52 % [1 to 134]*
INTEX-B flights	*16-Mar-2006*	*15-May-2006*	*−3 °C [−48 to +29]*	*34 % [1 to 136]*
PASE flights	*03-Aug-2007*	*09-Sep-2007*	*+23 °C [−11 to +27]*	*78 % [1 to 110]*
PEM Tropics flights	*11-Mar-1991*	*11-Apr-1991*	*+14 °C [+10 to +15]*	*64 % [0 to 109]*
SAS flights	*22-Jun-2013*	*08-Jul-2013*	*+16 °C [−19 to +27]*	*58 % [1 to 101]*
TRACE-P flights	*24-Jan-2001*	*10-Mar-2001*	*−1 °C [−39 to +27]*	*40 % [0 to 100]*

Referee #2 (Remarks to the Author):

This paper presents novel and important new experimental evidence for the role of methyl sulfonic acid (MSA) in the formation of new particles in the atmosphere. The role of reduced sulfur in new particle formation has been known for several decades and this has been recognised as playing an essential role in repopulating the background atmosphere with new particles after loss via precipitation has occurred. Thus far, the main species identified as driving this cycle has been sulfuric acid, an oxidation product of di-methyl sulfide (DMS). This cycle has been speculated as being a very important climate feedback since warmer temperatures increase biological productivity and the increase in resulting emissions leads to greater particle formation rates and hence cloud droplet numbers, enhancing cloud brightness. This feedback, known as the CLAW hypothesis, has remained an important scientific challenge since it is the main particle generation mechanism over the ocean in the background, pre-industrial state. Hence ensuring it is well represented in global models is important. However, much remains uncertain.

What the paper demonstrates for the first time is that another oxidation product of DMS, MSA, can, at low temperatures, also play an important role in particle formation in marine environments The role of MSA in new particle formation has been speculated previously and some experimental and theoretical work has indicated it may be playing a role but this has never been unequivocally demonstrated previously. The experiments, carried out in the globally unique CLOUD experimental facility, show that at temperatures below -10C, MSA and ammonia gases can drive a particle formation, or nucleation, process that is competitive with that of the sulfuric acid-ammonia system previously considered in global models to drive marine particle formation. The paper also shows for the first time, that MSA can take part in synergistic multi-acid nucleation along with sulfuric acid at rates that are over an order of magnitude larger than the particle formation rates of sulfuric acid alone, since the total available vapour concentrations are elevated. Lastly, the paper shows that at temperatures up to 9C (the warmest temperatures presented in the work) MSA enhances particle growth at sizes above 1.8 nm at the kinetic limit and even when ammonia is limited. This latter finding reinforces the earlier work of Johnson and Jin (reference 47) but covers a wider range of temperatures. As the authors point out this is important since growing newly formed particles rapidly reduces losses by diffusion to larger particles and increases the fraction growing to sizes capable of acting as cloud condensation nuclei. Taken together, these findings are very important results that have wide importance across fields of global atmospheric and marine science, climate science and physical chemistry and would certainly be worthy of publication in Nature.

The experimental work is of extremely high quality, the methodology is clear and well described. The facility used and the instrumentation deployed have been published extensively elsewhere. Though they are state of the art and allow insight into the chemical drivers of systems such as this, the novelty is in the particular chemical system and its wide ranging importance to the global atmosphere. These are certainly the right experiments and methods to address the problem.

My main comments are on the discussion and atmospheric implications. The current focus of the discussion is on high latitude marine boundary layers. This is for obvious reasons since DMS oxidation increasingly favours the MSA channel over that of sulfuric acid at colder temperatures and the results show MSA nucleation becomes important at temperatures lower than -10C. However, the impact may be significantly less than the discussion section concludes (see below). On the other hand, the findings presented in the paper may have important ramifications in other regions such as the upper troposphere and the role of MSA in growing particle clusters formed by sulfuric acid-ammonia may be very important globally. These two important aspects are not considered in the same detail in the discussion yet may well be as, if not even more important. I have detailed some of this below. This is not to diminish the findings but rather it is important to explore the wider importance.

We thank the reviewer for highlighting the importance of this work for the climate. We agree with the reviewer that MSA may contribute to free tropospheric aerosol formation processes, including in the upper troposphere, which would extend the implications of this study to the global scale rather than confining them to polar regions. We also agree with the reviewer that the enhancement of particle growth rate—in particular since it does not require (rare) base vapours—is especially important and is likely to play an important global role in determining the fraction of new particles that reach CCN sizes and strongly influence climate. The importance of the contribution of MSA to free tropospheric particle formation is now discussed in detail in our responses below and in the revised manuscript, supported by aircraft measurements and modelling results.

- <u>General comments</u>

17) In the discussion section, the authors make a strong case for MSA being important in new particle formation at high latitudes. The case for this is based on their findings that MSA plays an important role in elevating nucleation rates when combined with sulfuric acid (and also iodine acids if available) along with the availability of a base at temperatures of -10C and below. They also show field observations of MSA from a number of coastal stations at high latitudes that show MSA exists at equivalent concentrations to sulfuric acid. Their conclusion is therefore that "Our measurements show that MSA is a key biogenic precursor for new particle formation in cold marine regions yet remains unaccounted in global climate models.". However, this is less convincing than first appears. Marine biochemistry is strongly seasonal, with maximum emissions of DMS taking place in summer months when sea-ice recedes, biological activity is at its strongest and temperatures are warmer. Under such conditions, air temperatures over the ocean in the polar regions are typically much warmer than -10C, often closer to, or even above, +10C. Under these conditions the experimental work presented shows no role for MSA in nucleation alone, though clear evidence of effective contribution to growth of particles above 1.8 nm. Of course, temperatures at boundary layer top will be cooler, likely by 5 to 10C, even so it is not obvious that the cold temperatures and reduced sulfur source strengths are coincident in space and time to have widespread impact. It would have been really good to see some model

predictions of the effect of including MSA in nucleation under the prevailing conditions and emissions, which would offer more evidence of the scale of the effects. At the very least the above needs careful discussion in the final section to highlight the need for further work to address the key questions.

We agree with the reviewer that our original statement needed refinement and further justification.

First, we have now added Extended Data Fig. 6 to show global SA and MSA measurements in the free troposphere from aircraft, and we also include a new lower panel in both that figure and Fig. 5 to show the *simultaneous* ratio of MSA to SA at these sites. This ratio predominantly lies in a narrow range of around 0.7–1.3 i.e. MSA and SA concentrations in the remote marine atmosphere and free troposphere are comparable and tightly correlated, so their comparable contribution to particle nucleation and growth below −10 °C is highly significant.

Second, to evaluate the global importance of MSA-driven nucleation, we have incorporated our experimental measurements of MSA-driven nucleation and growth in the EMAC Earth system model. Details of the model, including the representation of the DMS oxidation cycle and the parameterisation of our measurements, are provided in the response to Comment 3 above, along with comparisons between the modelled and observed SA and MSA concentrations (Fig. R2). As discussed in Comment 3, our simulations indicate climatically-important increases in CCN (0.4 %) due to MSA in the polar boundary layer and across extensive regions of the marine free troposphere (Extended Data Fig. 7). Our simulations support the conclusion that MSA is a major contributor of NPF and, moreover, CCN in cool, pristine marine environments.

<u>Revised text in Main text (lines 320–333)</u>:

To evaluate the potential impact of MSA on global new particle and CCN formation, we have carried out an initial study with the EMAC (ECHAM/MESSy Atmospheric Chemistry) Earth system model, which incorporates multicomponent parameterisations of atmospheric new particle formation from CLOUD experiments. For the present study, the baseline CCN concentrations consider only SA(-NH_3), calculated using the Dunne et al. parameterisation[41], together with all primary aerosol emissions. After incorporating our new results in the EMAC model (see Methods for further details), our simulations indicate that MSA enhances CCN concentrations especially strongly in the Arctic and Antarctic coastal regions, from the boundary layer up to at least 4 km altitude (Extended Data Fig. 7). These results are supported by recent observations of elevated gaseous MSA from aerosol evaporation during katabatic outflow from the Antarctic continent, which draws in upper tropospheric air from over the Southern Ocean[42]. Earth system models systematically underestimate CCN by more than 50 % over the Southern Ocean, producing a large positive radiation bias in this climatically-important region [43,44]. Our findings suggest that this bias may be largely eliminated by including the contribution of MSA, which is unaccounted in current climate models.

18) I also note that the data sets included in figure 5 all come from the co-authors yet there are many other data sets from other studies covering wider parts of the marine boundary layer across the globe that contextualise the global picture. These should be included or at least discussed.

We would like to clarify that open datasets that contain all three acids are extremely rare, considering the very recent finding of the importance of iodic acid [81]. Therefore, the data originally shown in Figure 5 were primarily compiled from recent (last 10 years) non-flight measurements, representing all openly-available observations of gas-phase SA, MSA, and IA concentrations measured by chemical ionisation mass spectrometry. However, in response to the reviewer's request, we have updated Figure 5 to include two additional datasets: one based on ground-based measurements from the OASIS Barrow Spring Intensive Campaign (2009) conducted at Utqiaġvik (formerly Barrow), Alaska [6], and another from a shipborne campaign in the Antarctic West Pacific Ocean sector (90–160 °E) during the CAPRICORN-2 (2018) Southern Ocean voyage which was recently made available by the authors [7]. We also removed the Hyytiälä measurements from the figure due to redundant geographic representation in comparison to the two nearby sites at Helsinki and Värriö. The updated plot is shown below.

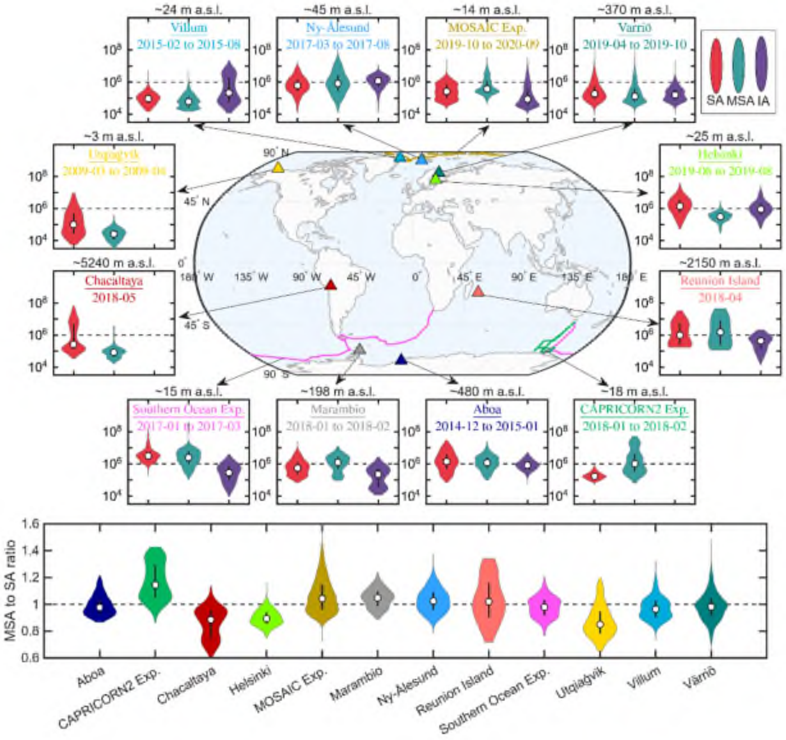

Revised Figure 5. Updated global map of SA, MSA and IA observations from ground stations and ship expeditions. The figure now includes two additional datasets (Utqiaġvik and CAPRICORN-2 expedition). A lower panel has also been added to show simultaneous MSA-to-SA ratios, included in response to Reviewer 3's suggestion.

Additionally, since most gas-phase MSA and SA measurements in the marine boundary layer have been obtained from airborne campaigns, we have included a new figure in the Supplement summarizing all available observations (Table R3, Extended Data Fig. 6). A discussion of these

airborne datasets and their relevance to our study have been added to the revised manuscript, addressing Referee Comment 19 regarding the free-tropospheric relevance of the proposed MSA aerosol nucleation mechanisms.

Table R3. Summary of openly available airborne datasets reporting gas-phase MSA and SA concentrations.

Mission / Campaign	Date	Measurement Region / Area	Platform
**ACE-1:** First Aerosol Characterization Experiment [2]	1995	Southern Ocean, near Tasmania (Cape Grim and Macquarie Island region)	NCAR C-130
**PEM-Tropics B**: Pacific Exploratory Missions [1]	1999	Tropical and subtropical Pacific Ocean	NASA P-3B
**TRACE-P:** The NASA Transport and Chemical Evolution over the Pacific[3]	2001	Eastern Asia and the western Pacific Ocean	NASA P-3B
**INTEX-B:** Intercontinental Chemical Transport Experiment – Phase B [8]	2006	Mexico City and Eastern Pacific Ocean near Vancouver.	NCAR/NSF C-130
**PASE**: Pacific Atmospheric Sulfur Experiment [4]	2007	Central tropical Pacific (around Christmas Island, 2° N, 157° W)	NCAR C-130
**ARCTAS:** Arctic Research of the Composition of the Troposphere from Aircraft and Satellites [5]	2008	Alaska, Thule Greenland, and Iqaluit Canada and Cold Lake, Edmonton Canada	NASA DC-8
**SAS:** Southeast Atmosphere Study [9]	2013	South-eastern United States	NSF/NCAR C-130

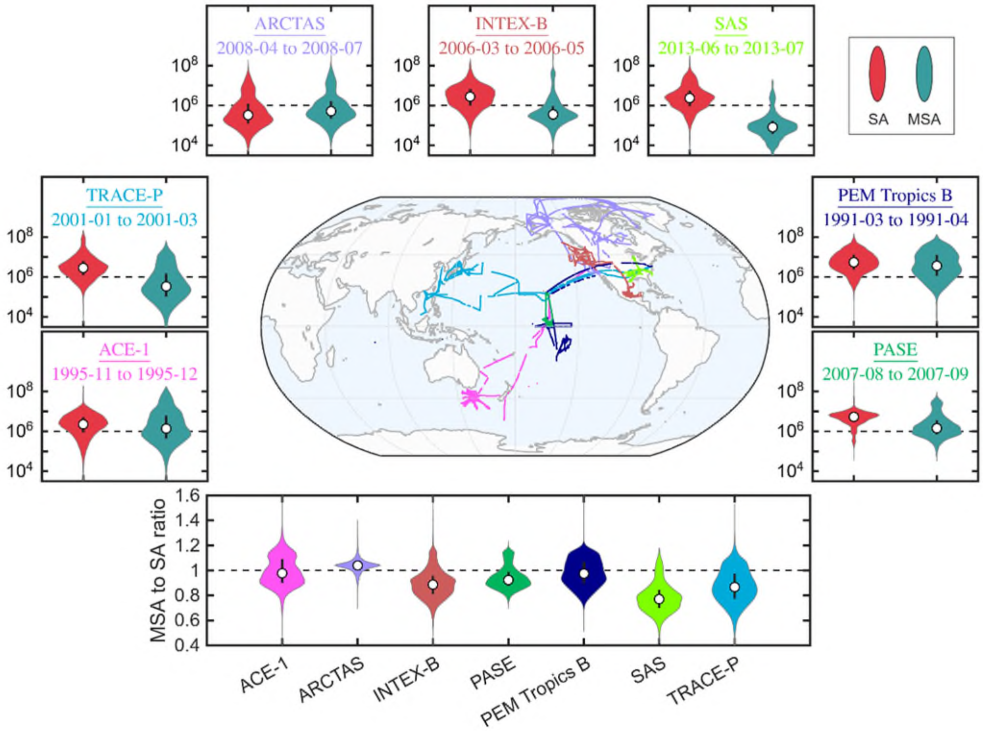

Extended Data Fig. 6. Global SA and MSA observations from aircraft. *Violin plots showing airborne measurements of sulfuric acid (red), and methanesulfonic acid (green) along the flight paths indicated on the map. The surrounding boxes include the name and dates of each campaign. The lower panel shows the simultaneous MSA/SA ratio for each campaign, with a linear y scale. The width of each violin is proportional to the frequency of observation at the corresponding vapour concentration indicated on the y axis. The white circles represent the median, and the attached vertical bars denote the interquartile range (25th to 75th percentiles). Details on the flight campaigns and data sources are provided in the Methods.*

19) The upper atmosphere may well be an important environment. There are some ground based observations at altitude presented (Scholz et al from Chacaltaya), though the air masses sampled are not of recently uplifted air. Nevertheless, it is worth considering these data since MSA is relatively low compared to SA suggesting less of a role. It is worth including an argument in the paper that there is a clear need to sample air masses of marine air that have recently been lifted to mid and high altitudes to examine ratios and abundances of MSA and SA in these environments. This is worth noting since there is a major question over whether MSA is important in these regions.

Indeed, the free troposphere (including the upper troposphere) is an important environment where elevated MSA concentrations can occur, as we discuss below. Before addressing this, we would like to note that Figure 5 shows two high-altitude sites - Chacaltaya and Réunion Island. For clarity, we have now added the altitudes of these sites directly to the figure rather than listing them only in the Methods. At Chacaltaya, the SA-to-MSA ratio cannot be meaningfully interpreted in the context of DMS oxidation, since SA levels there are strongly influenced by volcanic emissions. As the authors of that dataset note, "*our dataset does not allow any conclusions regarding the production of SA by DMS oxidation*." In contrast, the Maïdo station on Réunion Island shows elevated MSA concentrations with respect to SA.

The Maïdo observations are consistent with previous airborne observations (newly summarised in Extended Data Fig. 6) showing elevated gas phase MSA concentrations measured aloft. Such enhancements in free tropospheric MSA can occur when ocean-emitted DMS is efficiently transported upward from the surface mixed layer into the upper decoupled layer by rising thermals and cumulus clouds[82], where it is oxidised at low temperatures to form MSA [83]. Alternatively, elevated MSA concentrations aloft may result from particles transported from the boundary layer that subsequently release MSA by evaporation under dry [84,85] or acidic [84,86] free-tropospheric conditions.

Most marine flights were conducted over the Pacific Ocean and revealed noticeable variability in MSA concentrations and in the MSA-to-SA ratio. The highest MSA levels occurred during the PEM-Tropics campaigns (tropical Pacific, from Hawaii to near New Zealand), followed by ACE-1 (south of Australia and the southwest Pacific) and PASE (central tropical Pacific). These measurements over the remote tropical and subtropical Pacific reflect strong biogenic sulfur emissions from phytoplankton, with particularly high MSA concentrations observed during PEM-Tropics B, which focused on the tropical lower troposphere (< 6 km). Flights over the western North Pacific (East Asian outflow) recorded lower average MSA levels, though concentrations occasionally reached 6×10^{7} cm^{-3}. Elevated MSA was also observed during the ARCTAS campaign, which sampled Arctic regions (Alaska, Greenland, and Iqaluit, Canada) and sub-Arctic air masses (Cold Lake, Alberta). In contrast, flights over continental regions such as those in the INTEX-B campaign (Mexico City and the eastern Pacific near Vancouver Island) and the SAS campaign (southeastern US) measured lower MSA concentrations (average below 1×10^{6} cm^{-3}).

Both campaigns also exhibited the lowest MSA-to-SA ratios among all datasets. Across all observations, however, the MSA-to-SA ratio generally fell within the range 0.7–1.3.

In summary, aircraft observations confirm that MSA is present throughout the free and upper troposphere across both tropical and polar regions, with concentrations occasionally reaching 1×10^{8} cm^{-3}. The measurements span a broad domain from ~70 °N to 45 °S and 30 °E to 180 °W, encompassing the Pacific basin, Southern Ocean, and Arctic regions. However, airborne observations remain sparse over continental areas and other ocean basins. Concurrent measurements of precursor gases, clusters, and new particle formation are needed to quantify MSA's contribution to atmospheric nucleation across these diverse global environments.

Revised text (lines 294–300):
To assess the potential impact of MSA on new particle formation, we have assembled global concentrations of SA, MSA, and IA from cool marine regions, based on measurements made from ground stations and ship expeditions (Fig. 5) and from aircraft (Extended Data Fig. 6). These vapours generally coexist at all these locations with concentrations mainly between 10^{5} and 10^{7} cm^{-3}. Moreover, the MSA and SA concentrations are closely matched, with simultaneous MSA/SA ratios predominantly in the range 0.7–1.3 (bottom panels in Fig. 5 and Extended Data Fig. 6).

20) In the discussion, less emphasis is placed on the role of MSA in growing newly formed clusters at rates that exceed those observed in the sulfuric acid system alone. The authors observed this to happen at all temperatures and in the absence of any base in the experiments. Since the biggest loss of very small particles is diffusion to existing particle surfaces, faster growth leads to higher survival probabilities and hence the likelihood that more particles survive to act as cloud condensation nuclei. This may yet be of greater important over many geographical regions than its role in nucleation directly and a discussion of this would be very good to see.

We agree with the reviewer the influence of MSA on particle growth is likely to be far more widespread geographically than its contribution to nucleation, which requires lower temperatures and the presence of base vapours. We have therefore highlighted the significance of MSA-driven particle growth in a dedicated paragraph in the "Atmospheric implications" section as follows:

Revised text (lines 314–319):
On the other hand, even in the absence of base vapours, we find that MSA accelerates the growth rates of particles above 1.8 nm, at +9 °C and below. This may be important for CCN concentrations over wide geographical regions of the cool boundary layer and free troposphere since the major loss mechanism of small, highly mobile new particles is scavenging by pre-existing larger particles. Accelerated growth rates will result in reduced losses of newly-formed particles and a higher likelihood that they survive to reach CCN sizes.

21) As climate is warming, the pre-industrial background may well be different to the background of today since the MSA formation pathway is quite temperature dependent and increases at low temperature. This, combined with the enhanced role of MSA at colder temperatures and the greater expanse of sea ice may well mean that particle formation in the pre-industrial may need to be considered carefully and include MSA. The authors may want to include a short discussion on this.

We agree that the strong temperature dependence of MSA formation and the expanded cold ocean surface in the pre-industrial period likely increased the role of MSA in particle formation. We have revised the text accordingly:

Original text (lines 330–333):

Our findings also imply that the marine biosphere produced more CCN in the pristine pre-industrial marine atmosphere than presently thought. This may affect estimates of Earth's radiative forcing from anthropogenic aerosol and, in turn, projections of warming later this century.

Revised text (Lines 334–346):

Our results imply that purely biogenic marine sources produced more CCN in the pristine pre-industrial marine atmosphere than presently thought. Evaluating the influence of MSA on the baseline preindustrial aerosol state will require careful consideration of the effects of cooler temperatures, which would enhance the yield of MSA from DMS compared with the present-day, and of a greater extent of sea ice, which would displace phytoplankton and their DMS emissions. Increased biogenic CCN will tend to reduce estimates of Earth's effective radiative forcing from present-day anthropogenic aerosol, and affect projections of warming later this century. Sulfur dioxide emissions are now decreasing, which may return the future aerosol state of the atmosphere towards pre-industrial levels, buffered by biogenic particle formation in a warmer climate. Although future globally averaged seawater DMS concentrations are predicted to decrease, emissions of DMS into the atmosphere are expected to increase due to rising surface wind speeds and sea surface temperatures [87]. *Together with falling sulfur dioxide levels, this suggests an increasing importance of MSA and of ammonia emissions for aerosol effective radiative forcing in the post fossil fuel climate.*

22) Lastly, there is no discussion of the typical base concentrations at high latitudes. This would be good to see in the context of the importance of the processes being discussed in different parts of the atmosphere.

Ammonia concentrations in high-latitude regions exhibit pronounced spatial and temporal variability, driven by the interplay of strong but localised biogenic emissions and episodic long-range transport. On the one hand, local sources, such as seabird and penguin colonies, generate substantial hotspots with elevated mixing ratios. For example, hourly measurements in the summertime Arctic marine boundary layer have been reported between 40 and 870 pptv [88], while Antarctic coastal sites near penguin colonies have recorded values as high as 13,000 pptv [89]. The ammonia emissions are found to continue even after migration of the birds since they leave behind so-called ornithogenic soils, enriched with guano and vast amounts of organic matter and nutrients

brought from the ocean to the land. On the other hand, the potential for long-range transport of ammonia differs markedly between the two hemispheres. In the Southern Hemisphere, Antarctica's atmosphere is largely isolated from mid-latitude pollution by the Antarctic Circumpolar Vortex, which acts as an effective dynamical barrier. However, a strong katabatic outflow from the Antarctic continent draws vapours from the surface of the Southern Ocean and the coastal regions to the upper troposphere. In contrast, the Arctic atmosphere is more permeable to meridional inflow from lower latitudes. Observational evidence indicates that particulate ammonium in the Arctic free troposphere frequently originates from anthropogenic emissions in Europe and East Asia, implying uplift, oxidation, and subsequent northward transport of NH_3 [90]. In addition, mid-latitude outflow, particularly from boreal wildfire plumes, has been linked to enhanced total ammonia columns in the high Arctic during summer [91].

Revised text (lines 306–311):
An important source of NH_3 in polar regions is guano from ubiquitous seabird colonies, which can produce concentrations between 10 and 1000 pptv in both the Arctic [88] and Antarctic [89] boundary layers. The ammonia emissions persist even after migration of the birds since they leave behind so-called ornithogenic soils, enriched with guano and vast amounts of organic matter and nutrients brought from the ocean to the land. In the Arctic, long range transport of anthropogenic emissions can further elevate NH_3 levels [91]

- Minor comments

23) Lines 71-72: "At temperatures of 9 °C or lower and relative humidities (RH) above 40 %, MSA drives kinetic growth of particles above 1.8 nm diameter. These rapid growth rates were measured……" in the systems considered, would this be true of the ambient environment? The preceding sentence offers no insight into whether the growth rates were fast or slow. It would be helpful to say how much the rate is enhanced. Possibly something like: By a factor of 1.5 at equal concentrations of MSA and SA or similar.

We thank the referee for pointing out our lack of clarity here; "kinetic growth" is a confusing shorthand for growth rate at the kinetic limit determined by the molecular collision rate, i.e. the fastest growth rate possible. Following the reviewer's recommendation, we have also added a sentence in the abstract to quantify the contribution of MSA. The revised text is as follows:

Revised text (lines 67–68):
Under cool and humid conditions (below 9 °C and above 40 % RH), MSA drives the growth of particles above 1.8 nm at the kinetic limit.

Revised text (lines 70–73):
Since MSA and SA generally coexist at similar concentrations in cool marine regions, our findings indicate that nucleation rates may be accelerated by about tenfold and growth rates by about twofold compared with SA-NH_3 alone.

24) Lines 114-116: “In summary, while laboratory and observational studies indicate that MSA contributes to the growth of pre-existing particles, the questions of its contribution to particle nucleation and early growth under atmospheric conditions remain open.” The Johnson and Jin reference (#47) conducted experiments on nucleating particles and their conclusion, as stated on line 111, is that MSA does enhance particle growth at small sizes under warm temperature conditions, so perhaps the above statement needs re-wording to reflect that earlier finding.

We have rephrased this paragraph to better reflect the works of Finlayson-Pitts et al. and also Johnson and Jen.

Original text (lines 111–116):

Experimentally MSA has been shown to enhance SA particle formation[19] *and form particles with amines and NH3*[24,28,35]*. However, these experiments were conducted above 20 °C and at MSA concentrations above* 10^8 cm^{-3}*, which are well above median MSA atmospheric concentrations of around* 10^6 cm^{-3}*. In summary, while laboratory and observational studies indicate that MSA contributes to the growth of pre-existing particles, the questions of its contribution to particle nucleation and early growth under atmospheric conditions remain open.*

Revised text (lines 109–123):

Experimentally, MSA has been shown to form particles with amines and NH_3 *at high (ppbv) precursor concentrations, and warm temperatures* [24,28,35]*. These studies have also shown the importance of water for formation and growth of MSA particles. A more recent study* [19] *has measured SA-MSA and MSA-MSA dimers in flow tube experiments at 25 °C and 20 % RH with* 2×10^8 cm^{-3} *MSA, (2–8)*$\times10^8$ cm^{-3} *SA and base vapours (ammonia and amines). However, only relatively small (20–30 %) increases, or even decreases, in particle counts were measured when injecting* 2.5×10^8 cm^{-3} *MSA into SA-base vapour mixtures* [19]*.*

In summary, ambient observations have established that MSA is an important contributor to particle mass, and laboratory studies have shown both the importance of high RH conditions and also that mixed SA-MSA dimers and larger particles form in the presence of base vapours at warm temperatures, and acid concentrations exceeding typical atmospheric levels. However, MSA has so far remained unaccounted in global climate models since quantitative experimental measurements of particle nucleation and growth rates with MSA have not yet been reported at the cool temperatures and low vapour concentrations representative of the polar boundary layer and free troposphere.

25) Lines 301-303: Whilst the plot undoubtedly shows that MSA and SA vapours do contribute to particle growth, it is less clear why these vapours are “responsible for the particle growth” since there is no closure between the FIGAERO observations and the particle mass change. How have some other, undetectable species that co-varies with MSA and SA been ruled out?

Indeed, a mass closure is not possible because water and ammonia cannot be measured with the FIGAERO operating with the negative polarity chemical ionisation. Nonetheless, we concluded

that MSA and SA are the sole vapours responsible for particle growth because they were the only detected compounds in the particle phase. No other peaks appeared in the mass spectrometer in significant amounts when these particles were thermally evaporated. We have cross-checked the mass spectrometer with an expanded DMS oxidation product peak list, and the only peaks above the detection limit remain those of SA and MSA. We revised the text accordingly.

Original text (lines 301–303):

The FIGAERO measurements provide direct evidence that MSA and SA are the vapours responsible for particle growth, and not an unidentified compound from DMS oxidation.

Revised text (lines 279–281):

The only particle-phase compounds detected by the FIGAERO in these experiments were SA and MSA. This provides direct evidence that particle growth is driven by SA and MSA alone rather than by some other, unidentified oxidation products of DMS.

26) Lines 306-307: This statement rather misrepresents the geographical variations in the concentrations of the different precursors vapours. Iodine acids are widely seen in enhanced concentrations in coastal regions, however, are at much lower concentrations over the clean, open ocean. Since many of the sites in figure 5 are at coastal sites the state is true but doesn't reflect the spatial distribution observed. The cruise data in the figure show substantially lower iodine as does the Reunion site, which has very little near shore and lacks a continental shelf in the immediate ocean. This needs to be reflected somewhat better.

We agree with the referee and have revised the text to correct this mistake, as follows:

Original text (lines 306–307):

At almost every site, all three vapours coexist in the atmosphere at comparable concentrations.

Revised text (lines 297–298):

These vapours generally coexist at all these locations with concentrations mainly between 10^5 *and* 10^7 cm^{-3}.

We have also added a new panel at the bottom of both Fig. 5 and Extended Data Fig. 6 to show that, in the case of MSA and SA, these vapours do generally coexist in the atmosphere at comparable concentrations.

27) Line 331: this should read the "marine biosphere"

Yes indeed; the text has now been corrected as follows:

Original text (lines 330–332):

Our findings also imply that the biosphere produced more CCN in the pristine pre-industrial marine atmosphere than presently thought.

Revised text (lines 334–335):

Our results imply that purely biogenic marine sources produced more CCN in the pristine pre-industrial marine atmosphere than presently thought.

28) Line 332: Figure 5 does not show data from the upper troposphere, it would be good to include a discussion of concentrations in the upper troposphere based on published literature and examine potential impacts in more detail in these regions.

This point has been addressed in our responses to Comments 18 and 19 above.

29) Line 332: As currently presented the colour of the triangles in figure 5 have no meaning. This could be used to better illustrate which plot is from which location or even better use different colours to represent different sites.

The triangle colours in the figure do indeed correspond to different sites or studies. To make this clearer, we now use consistent colours for the subplot titles and for the new lower panel showing the MSA-to-SA ratio, added in response to Reviewer 3 (comment 37). Please see the updated figure in the answer to comment 18.

30) Line 661: Why does the MSA decrease by 50% during the course of the experiment (Ext data fig 2 panel D) but that of H_2SO_4 does not?

To address the reviewer's question, we added an extended time-series plot of MSA and SA concentrations around the experiment period (Figure R8). As shown in panel a, the observed decrease in MSA coincides with an increase in the condensation sink from particle growth. There is also a decrease in SA, but by a smaller amount. The decrease in MSA between the start and end of the steady-state measurement is around 15 % and the SA decrease is around 5 %. These are probably significant differences but nevertheless rather small variations and well within normal levels of experimental variation due to effects like changes of the condensation sink.

To investigate other potential causes of the variation of MSA and SA, we have plotted the intensity of UV illumination in the chamber, as measured by the photodiodes (Figure R8 B). The chamber was initially illuminated with Hamamatsu Hg–Xe UV lamps, and at 18:40 a krypton fluoride excimer laser (UVX) was activated to enhance MSA and SA production. This corresponds to an increase in photodiode counts from ~6,800 to ~12,500 (the photodiode counts do not directly reflect the O_3 photolysis rate). Subsequent photodiode reductions indicate the UV laser was close to the end of its gas filling. The DMS concentration also increased by around 5 % during this experiment. The net effect of these changes in experimental conditions and condensation sink resulted in the MSA and SA reductions of 15% and 5%, respectively. The main reason for the overall reduction is likely due to the falling intensity of the UV laser.

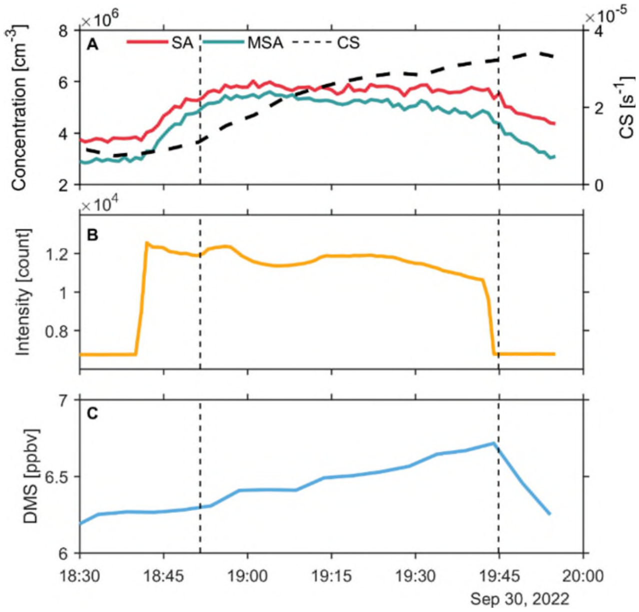

Figure R8. Time series of (A) gas-phase SA and MSA concentrations and condensation sink (CS), (B) Photodiodes signal intensity, and (C) DMS concentration. The vertical dashed lines indicate the start and end of the experiment originally presented in Ext. Data Figure 2.

Referee #3 (Remarks to the Author):

Baalbaki et al. present new and convincing measurements of the role of MSA in particle nucleation. It is well known that MSA contributes to particle growth to CCN sizes, but direct evidence that MSA contributes to particle nucleation has been elusive. This paper provides clear experimental proof and should certainly be published. It is an editorial decision of whether this is sufficient for publication in a broad interest journal such as Nature.

- General Comments:

31) I would expect for publication in Nature that the authors would show that this enhanced nucleation rate had an impact on radiative cooling or at least on CCN concentrations. Specifically, I was waiting to read this in the last section of the paper. The authors could use the measurements of MSA/SA (shown in Figure 5) to determine what the expected enhancement in the nucleation rate would be in these locations. They could then partner with an aerosol process level modeling team to determine the impact of that enhancement in nucleation rates on CCN (at least and ideally on radiative cooling). That would certainly be a nature paper if there was a sizeable impact. While this paper is certainly a great advance and is publishable as is in a specialized journal, it is not clear what the impact of an increase in the nucleation rate is on CCN number (and hence climate) in these regions of the atmosphere.

We thank the reviewer for highlighting the importance of assessing the climate relevance of enhanced nucleation. In response to this and to similar requests from the other referees (Comments 3 and 17), we have now incorporated our experimental measurements in the EMAC Earth system model. Details of the model, including the representation of the DMS oxidation cycle and the parameterisation of our measurements, are provided in the response to Comment 3 above, along with comparisons between the modelled and observed SA and MSA concentrations (Fig. R2). As discussed in Comment 3, our simulations indicate climatically-important increases in CCN (0.4 %) due to MSA in the polar boundary layer and across extensive regions of the marine free troposphere (Extended Data Fig. 7). As well as a description of the EMAC model in the Methods, we have added the following paragraph and figure to the manuscript to summarise the results of our simulations:

Revised text (lines 320–333):

To evaluate the potential impact of MSA on global new particle and CCN formation, we have carried out an initial study with the EMAC (ECHAM/MESSy Atmospheric Chemistry) Earth system model, which incorporates multicomponent parameterisations of atmospheric new particle formation from CLOUD experiments. For the present study, the baseline CCN concentrations consider only SA(-NH_3), calculated using the Dunne et al. parameterisation [41], together with all primary aerosol emissions. After incorporating our new results in the EMAC model (see Methods for further details), our simulations indicate that MSA enhances CCN concentrations especially

strongly in the Arctic and Antarctic coastal regions, from the boundary layer up to at least 4 km altitude (Extended Data Fig. 7). These results are supported by recent observations of elevated gaseous MSA from aerosol evaporation during katabatic outflow from the Antarctic continent, which draws in upper tropospheric air from over the Southern Ocean [42]. Earth system models systematically underestimate CCN by more than 50 % over the Southern Ocean, producing a large positive radiation bias in this climatically-important region [43,44]. Our findings suggest that this bias may be largely eliminated by including the contribution of MSA, which is unaccounted in current climate models.

- Detailed / Specific Comments:

32) Line 57: While DMS is a very large natural source of sulfur, passive volcanic emissions have recently been shown to be larger (at least in the Arctic). Reference is Jongebloed et al. GRL 2023, https://doi.org/10.1029/2022GL102061

We thank the reviewer for pointing this out. We have revised both the abstract and the introduction to clarify that volcanic emissions can be comparable to, or even exceed, DMS as a source of sulfur, based on the findings of Jongebloed et al. and recent results from the ATom mission on the marine sulfur cycle [92].

Original text (lines 57–58):
Dimethylsulfide (DMS; CH_3SCH_3), emitted by marine phytoplankton is the largest natural source of sulfur on a global scale.

Revised text (lines 60–61):
Dimethylsulfide (DMS; CH_3SCH_3) from marine phytoplankton is a major global source of atmospheric sulfur.

We now also include reference to Jongebloed et al. in the introduction section (see Comment 34 below).

33) Line 87: I am not sure that references 15 and 16 support the addition of “and, especially”. That implies that the nucleation rate is the key source of uncertainty in the impact of DMS on radiative cooling. I am not sure these references support that. I’d recommend removing “and, especially” unless there is a reference that states that nucleation rate is the limits our understanding. If that was the case, I’d actually recommend expanding on that point. It is not abundantly clear to me at this point in the paper what impact a doubling the nucleation rate in these regions would have on radiative cooling. It would be good to expand a bit on this point.

We agree with the referee and have now removed the phrase “and, especially”.

34) Line 95: Related to my comment on line 57, I’d add volcanic H2S/SO2 emissions.

In accordance with our response to Comment 32, the text has been revised as follows:

Original text (lines 93–96):
While MSA is primarily derived from DMS oxidation, SA can originate from other sources, such as anthropogenic sulfur dioxide (SO_2) and methanethiol, generally resulting in higher concentrations of SA compared with MSA.

Revised text (lines 92–94):
Although MSA originates mainly from DMS oxidation and, to a lesser extent from methanethiol (CH_3SH) [93], SA has additional sources such as volcanic sulfur dioxide (SO_2), hydrogen sulfide (H_2S) [94], and anthropogenic SO_2.

35) Figure 1: I really struggled to understand the coloured bands in panels B and C. As I understand, those coloured bands are for SA only. It makes it really hard to understand panels B and C since the x-axis as labeled is not SA. I get that the caption says all this, but I doubt the reader will understand this. I wonder if it would be better to remove the shaded regions from B and C? The point is clearly made in panel A when comparing the observed nucleation rate to SA. I think all readers will understand that the rate is faster than that supported by SA chemistry alone.

We thank the referee for pointing out these difficulties in understanding Fig. 1. We have therefore tried to clarify the figure better. We acknowledge that the shaded bands may be confusing without additional context. However, we have chosen to retain them for all three panels since they provide constant reference curves for each panel which i) demonstrate that SA alone cannot explain the measured nucleation rates, ii) that including MSA on an equal basis with SA brings the measurements into agreement with the curves and iii) that the nucleation rates are insensitive to MSA at +9 °C (new hollow red data points; see Comment 8 above for further discussion). We have accordingly modified the legend text and the caption as follows:

Revised text (lines 519–521):
The coloured bands indicate the expected SA-NH_3 nucleation rates under GCR conditions, calculated using the Dunne et al. parameterisation [41]. The bands are identical across all panels (with the x-axis interpreted as SA) and are colour-coded by temperature.

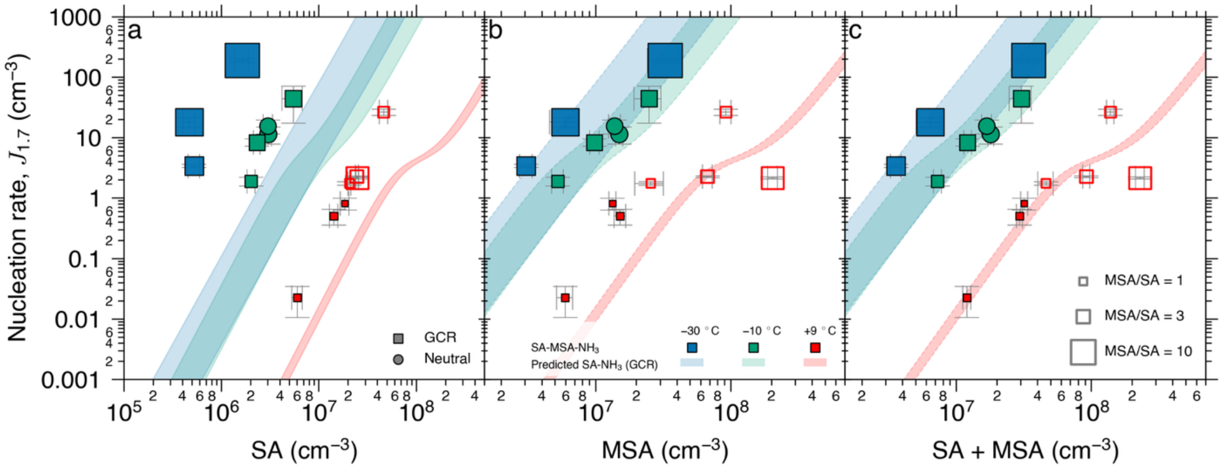

Revised Fig. 1 showing additional +9 °C experiments at higher MSA to SA ratio (hollow red squares, involving direct injection of MSA into the chamber from an evaporator) and updated legend.

36) Line 182: I'm not sure the experiments support the statement that MSA does not contribute to nucleation in the absence of base vapors like NH_3 even in the upper troposphere. Perhaps I am misunderstanding the experiment. Was nucleation not observed for MSA only experiments at -50C or was it that nucleation was not enhanced when MSA and SA were present in the chamber relative to SA alone? Is this still true at very high MSA/SA ratios that might be possible in the UT? I don't think there are many measurements of MSA/SA at 10km in convective outflow to be able to constrain what this ratio looks like? At -50C DMS oxidation proceeds almost exclusively down the OH addition pathway and it is possible that there are locations in the UT that have high MSA/SA ratios (this would require low sources of SO_2).

The −52 °C experiments presented in Extended Data Figure 2 show that nucleation was not enhanced when MSA was added to SA at equal concentrations of ~5×10^6 cm^{-3}; the rates remained indistinguishable from those for SA alone. We agree with the reviewer that, in the free troposphere, MSA concentrations could occasionally be substantially higher than those used in our experiments— up to ~1×10^8 cm^{-3} based on the compilation of aircraft datasets shown in Extended Data Fig. 6 (added in response to Comment 18).

In response to Comment 10, we have added a reference in the main text to additional experimental data showing that MSA alone, at concentrations up to 1.4×10^7 cm^{-3}, does not nucleate at −30 °C in the absence of NH_3 (Figure R4). We also measured that MSA does not nucleate without NH_3 at −52 °C, these results support our more-precise conclusion that MSA at atmospheric concentrations below ~10^7 cm^{-3} does not contribute to particle nucleation without a base vapour, even at temperatures as low as −52 °C. We have therefore corrected the text as described below.

Original text (lines 183–185):

These experiments show that MSA does not contribute to nucleation in the absence of base vapours like NH_3, even at upper tropospheric temperatures.

Revised text (lines 186–190):

We also conducted a sensitivity test in which MSA was directly injected into the chamber at concentrations up to 1.4×10^7 cm^{-3} at −30 °C, in the absence of NH_3 and SA. No nucleation was observed until NH_3 was introduced to the chamber. These experiments show that, at concentrations below around 10^7 cm^{-3}, MSA does not contribute to particle nucleation in the atmosphere in the absence of a base vapour, even at temperatures as low as −52 °C.

37) Figure 5: This figure is quite interesting, but I think some additional information or discussion is needed here. The violin plots cover an enormous dynamic range, so it is not clear that MSA/SA is similar in all locations. That is also not very intuitive given the wide range in altitude and temperatures. I think focusing on the MSA/SA ratio in these plots and discussing how that ratio connects back to the experiments would be of more interest.

We thank the referee for this excellent suggestion. We have now added a new lower panel to both Fig. 5 (ground stations and ship expeditions) and a new Extended Data Fig. 6 (aircraft) showing the violin plots for the simultaneous MSA/SA ratio at each station. These show that MSA and SA concentrations are comparable and tightly correlated, and are mainly in the range 0.7–1.3. We have also added the following text to indicate the implications of this ratio around 1.0:

Revised text (lines 297–303):

These vapours generally coexist at all these locations with concentrations mainly between 10^5 and 10^7 cm^{-3}. Moreover, the MSA and SA concentrations are closely matched, with simultaneous MSA/SA ratios predominantly in the range 0.7–1.3 (bottom panels in Fig. 5 and Extended Data Fig. 6). When combined with our results, it implies that present model studies may be underestimating the effective acid concentrations that drive particle nucleation and growth in cool marine regions by around a factor of 2, which translates to underestimating the nucleation rates by an order of magnitude, and growth rates by twofold.

References

1. Eisele, F. PEM Tropics B P-3B In-Situ Trace Gas Data. https://doi.org/10.5067/ASDC/SUBORBITAL/PEM-Tropics-B/TraceGas_AircraftInSitu_P3B_Data_1 (2025).
2. Eisele, F. & Mauldin III, R. L. ACE-1: NSF/NCAR C-130 OH, H_2SO_4, MSA Measurements. Version 1.0. null. https://doi.org/10.26023/FRZK-NVH9-CT0S. (2006).
3. Eisele, F. & Mauldin III, R. L. TRACE-P P3B-Aircraft EISELE.NCR data archive. https://www-air.larc.nasa.gov/pub/TRACEP/P3B-AIRCRAFT/AIRCRAFT/EISELE.NCR/ (2002).
4. Mauldin III, R. L. NCAR 4 Channel CIMS OH, H_2SO_4, and MSA Data. Version 1.0. NSF PASE study Aug-Sept 2007. https://doi.org/10.26023/P72H-HDDT-2Z0M (2011).
5. Mauldin III, R. L. ARCTAS OH, H_2SO_4, MSA DC-8 Aircraft In-situ Trace Gas Data. https://asdc.larc.nasa.gov/data/ARCTAS/TraceGas_AircraftInSitu_DC8_Data_1/ (2023).
6. Mauldin III, R. L. OH, MSA, and H_2SO_4 measurements during OASIS Barrow field intensive Spring 2009. NSF Arctic Data Center https://doi.org/10.5065/D6TM787P (2016).
7. Miljevic, B. & Osuagwu, C. G. CAPRICORN2 Gaseous methanesulfonic and sulfuric acid measurements from the RV Investigator voyage IN2018V01. v1.CSIRO. Data Collection. CSIRO. Data Collection. https://doi.org/10.25919/enc5-wh07 (2025).
8. Mauldin III, R. L. NSF/NCAR C-130 Measurements of OH, H_2SO_4, and MSA (TCIDC). https://data.eol.ucar.edu/dataset/93.238 (2006).
9. Mauldin III, R. L. SAS: CU CIMS OH, H_2SO_4, sCIs. Version 1.0. null. https://doi.org/10.26023/A58C-EF19-A80V (2014).
10. Baccarini, A. *et al.* Low-Volatility Vapors and New Particle Formation Over the Southern Ocean During the Antarctic Circumnavigation Expedition. *J. Geophys. Res. Atmos.* **126**, e2021JD035126 (2021).
11. Beck, L. J. *et al.* Differing Mechanisms of New Particle Formation at Two Arctic Sites. *Geophys. Res. Lett.* **48**, (2021).
12. Boyer, M. *et al.* The annual cycle and sources of relevant aerosol precursor vapors in the central Arctic. *EGUsphere* 1–49 (2024) doi:10.5194/egusphere-2023-2953.
13. Jokinen, T. *et al.* Ion-induced sulfuric acid–ammonia nucleation drives particle formation in coastal Antarctica. *Sci. Adv.* **4**, eaat9744 (2018).
14. Jokinen, T. *et al.* Measurement report: Long-term measurements of aerosol precursor concentrations in the Finnish subarctic boreal forest. *Atmos. Chem. Phys.* **22**, 2237–2254 (2022).
15. Quéléver, L. L. J. *et al.* Investigation of new particle formation mechanisms and aerosol processes at Marambio Station, Antarctic Peninsula. *Atmos. Chem. Phys.* **22**, 8417–8437 (2022).

16. Salignat, R. *et al.* Measurement report: Insights into the chemical composition and origin of molecular clusters and potential precursor molecules present in the free troposphere over the southern Indian Ocean: observations from the Maïdo Observatory (2150 m a.s.l., Réunion). *Atmos. Chem. Phys.* **24**, 3785–3812 (2024).
17. Thakur, R. C. *et al.* An evaluation of new particle formation events in Helsinki during a Baltic Sea cyanobacterial summer bloom. *Atmos. Chem. Phys.* **22**, 6365–6391 (2022).
18. Scholz, W. *et al.* Measurement report: Long-range transport and the fate of dimethyl sulfide oxidation products in the free troposphere derived from observations at the high-altitude research station Chacaltaya (5240 m a.s.l.) in the Bolivian Andes. *Atmos. Chem. Phys.* **23**, 895–920 (2023).
19. Johnson, J. S. & Jen, C. N. Role of Methanesulfonic Acid in Sulfuric Acid–Amine and Ammonia New Particle Formation. *ACS Earth Space Chem.* **7**, 653–660 (2023).
20. Wen, H. *et al.* Formation of atmospheric molecular clusters consisting of methanesulfonic acid and sulfuric acid: Insights from flow tube experiments and cluster dynamics simulations. *Atmospheric Environment* **199**, 380–390 (2019).
21. Hoppel, W. A., Fitzgerald, J. W., Frick, G. M., Larson, R. E. & Wattle, B. J. *Preliminary Investigation of the Role That DMS (Dimethyl Sulfide) and Cloud Cycles Play in the Formation of the Aerosol Size Distribution.* 33 https://apps.dtic.mil/sti/citations/ADA185002 (1987).
22. Hoppel, W. A. Nucleation in the MSA-water vapor system. *Atmos. Environ. (1967)* **21**, 2703–2709 (1987).
23. Dawson, M. L. *et al.* Simplified mechanism for new particle formation from methanesulfonic acid, amines, and water via experiments and ab initio calculations. *Proc. Natl. Acad. Sci. U.S.A.* **109**, 18719–18724 (2012).
24. Chen, H., Varner, M. E., Gerber, R. B. & Finlayson-Pitts, B. J. Reactions of Methanesulfonic Acid with Amines and Ammonia as a Source of New Particles in Air. *J. Phys. Chem. B* **120**, 1526–1536 (2016).
25. Dawson, M. L. *et al.* Amine–Amine Exchange in Aminium–Methanesulfonate Aerosols. *J. Phys. Chem. C* **118**, 29431–29440 (2014).
26. Ezell, M. J., Chen, H., Arquero, K. D. & Finlayson-Pitts, B. J. Aerosol fast flow reactor for laboratory studies of new particle formation. *J. Aerosol Sci.* **78**, 30–40 (2014).
27. Chen, H. *et al.* New particle formation and growth from methanesulfonic acid, trimethylamine and water. *Phys. Chem. Chem. Phys.* **17**, 13699–13709 (2015).
28. Chen, H. & Finlayson-Pitts, B. J. New Particle Formation from Methanesulfonic Acid and Amines/Ammonia as a Function of Temperature. *Environ. Sci. Technol.* **51**, 243–252 (2017).

29. Arquero, K. D., Xu, J., Gerber, R. B. & Finlayson-Pitts, B. J. Particle formation and growth from oxalic acid, methanesulfonic acid, trimethylamine and water: a combined experimental and theoretical study. *Phys. Chem. Chem. Phys.* **19**, 28286–28301 (2017).
30. Arquero, K. D., Gerber, R. B. & Finlayson-Pitts, B. J. The Role of Oxalic Acid in New Particle Formation from Methanesulfonic Acid, Methylamine, and Water. *Environ. Sci. Technol.* **51**, 2124–2130 (2017).
31. Xu, J., Finlayson-Pitts, B. J. & Gerber, R. B. Nanoparticles grown from methanesulfonic acid and methylamine: microscopic structures and formation mechanism. *Phys. Chem. Chem. Phys.* **19**, 31949–31957 (2017).
32. Xu, J., Perraud, V., Finlayson-Pitts, B. J. & Gerber, R. B. Uptake of water by an acid–base nanoparticle: theoretical and experimental studies of the methanesulfonic acid–methylamine system. *Phys. Chem. Chem. Phys.* **20**, 22249–22259 (2018).
33. Nishino, N., Arquero, K. D., Dawson, M. L. & Finlayson-Pitts, B. J. Infrared Studies of the Reaction of Methanesulfonic Acid with Trimethylamine on Surfaces. *Environ. Sci. Technol.* **48**, 323–330 (2014).
34. Perraud, V., Li, X., Jiang, J., Finlayson-Pitts, B. J. & Smith, J. N. Size-Resolved Chemical Composition of Sub-20 nm Particles from Methanesulfonic Acid Reactions with Methylamine and Ammonia. *ACS Earth Space Chem.* **4**, 1182–1194 (2020).
35. Perraud, V., Xu, J., Gerber, R. B. & Finlayson-Pitts, B. J. Integrated experimental and theoretical approach to probe the synergistic effect of ammonia in methanesulfonic acid reactions with small alkylamines. *Environ. Sci.: Processes Impacts* **22**, 305–328 (2020).
36. Kreidenweis, S. M. & Seinfeld, J. H. Nucleation of sulfuric acid-water and methanesulfonic acid-water solution particles: Implications for the atmospheric chemistry of organosulfur species. *Atmos. Environ. (1967)* **22**, 283–296 (1988).
37. Kreidenweis, S. M., Flagan, R. C., Seinfeld, J. H. & Okuyama, K. Binary nucleation of methanesulfonic acid and water. *J. Aerosol Sci.* **20**, 585–607 (1989).
38. Wyslouzil, B. E., Seinfeld, J. H., Flagan, R. C. & Okuyama, K. Binary nucleation in acid–water systems. I. Methanesulfonic acid–water. *J. Chem. Phys.* **94**, 6827–6841 (1991).
39. Wyslouzil, B. E., Seinfeld, J. H., Flagan, R. C. & Okuyama, K. Binary nucleation in acid–water systems. II. Sulfuric acid–water and a comparison with methanesulfonic acid–water. *J. Chem. Phys.* **94**, 6842–6850 (1991).
40. Hatakeyama, S., Izumi, K. & Akimoto, H. Yield of SO2 and formation of aerosol in the photo-oxidation of DMS under atmospheric conditions. *Atmos. Environ. (1967)* **19**, 135–141 (1985).
41. Dunne, E. M. *et al.* Global atmospheric particle formation from CERN CLOUD measurements. *Science* **354**, 1119–1124 (2016).

42. Miljevic, B. *et al.* Aerosol acidity controls methanesulfonic acid evaporation from aerosols during Antarctic katabatic outflow. *Commun Earth Environ* **6**, 1057 (2025).
43. Mallet, M. D. *et al.* Untangling the influence of Antarctic and Southern Ocean life on clouds. *Elementa: Sci. Anthropocene* **11**, 00130 (2023).
44. Fiddes, S. L. *et al.* The ACCESS-AM2 climate model underestimates aerosol concentration in the Southern Ocean; improving aerosol representation could be problematic for the global energy balance. *Atmos. Chem. Phys.* **25**, 16451–16477 (2025).
45. Elm, J. Clusteromics II: Methanesulfonic Acid–Base Cluster Formation. *ACS Omega* **6**, 17035–17044 (2021).
46. Li, S., Zhang, L., Qin, W. & Tao, F.-M. Intermolecular structure and properties of the methanesulfonic acid–ammonia system in small water clusters. *Chem. Phys. Lett.* **447**, 33–38 (2007).
47. Chen, D., Li, D., Wang, C., Liu, F. & Wang, W. Formation mechanism of methanesulfonic acid and ammonia clusters: A kinetics simulation study. *Atmos. Environ.* **222**, 117161 (2020).
48. McGrath, M. J. *et al.* Atmospheric Cluster Dynamics Code: a flexible method for solution of the birth-death equations. *Atmos. Chem. Phys.* **12**, 2345–2355 (2012).
49. Wang, L. Clusters of Hydrated Methane Sulfonic Acid CH3SO3H·(H2O)n (n = 1−5): A Theoretical Study. *J. Phys. Chem. A* **111**, 3642–3651 (2007).
50. Oussoumanou, O., Holtomo, O., Etindele, A. J. & Motapon, O. Atmospheric implications of the formation of water – sulphuric acid and water – methanesulfonic acid binary clusters. *Molecular Physics* **0**, e2520947 (2025).
51. Hodshire, A. L. *et al.* The potential role of methanesulfonic acid (MSA) in aerosol formation and growth and the associated radiative forcings. *Atmos. Chem. Phys.* **19**, 3137–3160 (2019).
52. Chin, M. *et al.* Atmospheric sulfur cycle simulated in the global model GOCART: Comparison with field observations and regional budgets. *J. Geophys. Res. Atmos.* **105**, 24689–24712 (2000).
53. Hoffmann, E. H., Heinold, B., Kubin, A., Tegen, I. & Herrmann, H. The Importance of the Representation of DMS Oxidation in Global Chemistry-Climate Simulations. *Geophys. Res. Lett.* **48**, e2021GL094068 (2021).
54. Shen, J. *et al.* High Gas-Phase Methanesulfonic Acid Production in the OH-Initiated Oxidation of Dimethyl Sulfide at Low Temperatures. *Environ. Sci. Technol.* **56**, 13931–13944 (2022).
55. Fung, K. M. *et al.* Exploring dimethyl sulfide (DMS) oxidation and implications for global aerosol radiative forcing. *Atmos. Chem. Phys* **22**, 1549–1573 (2022).
56. Hulswar, S. *et al.* Third revision of the global surface seawater dimethyl sulfide climatology (DMS-Rev3). *Earth Syst. Sci. Data* **14**, 2963–2987 (2022).

57. Baccarini, A. *et al.* Frequent new particle formation over the high Arctic pack ice by enhanced iodine emissions. *Nat. Commun.* **11**, 4924 (2020).
58. Kohl, M. *et al.* Numerical simulation and evaluation of global ultrafine particle concentrations at the Earth's surface. *Atmos. Chem. Phys.* **23**, 13191–13215 (2023).
59. Jöckel, P. *et al.* Development cycle 2 of the Modular Earth Submodel System (MESSy2). *Geosci. Model Dev.* **3**, 717–752 (2010).
60. Roeckner, E. *et al.* Sensitivity of Simulated Climate to Horizontal and Vertical Resolution in the ECHAM5 Atmosphere Model. *J. Climate* **19**, 3771–3791 (2006).
61. Pringle, K. J. *et al.* Description and evaluation of GMXe: a new aerosol submodel for global simulations (v1). *Geosci. Model Dev.* **3**, 391–412 (2010).
62. Rosanka, S. *et al.* How non-equilibrium aerosol chemistry impacts particle acidity: the GMXe AERosol CHEMistry (GMXe–AERCHEM, v1.0) sub-submodel of MESSy. *Geosci. Model Dev.* **17**, 2597–2615 (2024).
63. Ehrhart, S. *et al.* Two new submodels for the Modular Earth Submodel System (MESSy): New Aerosol Nucleation (NAN) and small ions (IONS) version 1.0. *Geosci. Model Dev.* **11**, 4987–5001 (2018).
64. Dietmüller, S. *et al.* A new radiation infrastructure for the Modular Earth Submodel System (MESSy, based on version 2.51). *Geosci. Model Dev.* **9**, 2209–2222 (2016).
65. Sander, R. *et al.* The community atmospheric chemistry box model CAABA/MECCA-4.0. *Geosci. Model Dev.* **12**, 1365–1385 (2019).
66. Shen, J. *et al.* High Gas-Phase Methanesulfonic Acid Production in the OH-Initiated Oxidation of Dimethyl Sulfide at Low Temperatures. *Environ. Sci. Technol.* **56**, 13931–13944 (2022).
67. Sander, R. *et al.* The photolysis module JVAL-14, compatible with the MESSy standard, and the JVal PreProcessor (JVPP). *Geosci. Model Dev.* **7**, 2653–2662 (2014).
68. Tost, H., Jöckel, P., Kerkweg, A., Sander, R. & Lelieveld, J. Technical note: A new comprehensive SCAVenging submodel for global atmospheric chemistry modelling. *Atmos. Chem. Phys.* **6**, 565–574 (2006).
69. Kerkweg, A. *et al.* Technical Note: An implementation of the dry removal processes DRY DEPosition and SEDImentation in the Modular Earth Submodel System (MESSy). *Atmos. Chem. Phys.* **6**, 4617–4632 (2006).
70. Pozzer, A. *et al.* Technical Note: The MESSy-submodel AIRSEA calculating the air-sea exchange of chemical species. *Atmos. Chem. Phys.* **6**, 5435–5444 (2006).
71. Fountoukis, C. & Nenes, A. ISORROPIA II: a computationally efficient thermodynamic equilibrium model for K+–Ca2+–Mg2+–NH4+–Na+–SO42−–NO3−–Cl−–H2O aerosols. *Atmos. Chem. Phys.* **7**, 4639–4659 (2007).

72. Tsimpidi, A. P., Karydis, V. A., Pozzer, A., Pandis, S. N. & Lelieveld, J. ORACLE (v1.0): module to simulate the organic aerosol composition and evolution in the atmosphere. *Geosci. Model Dev.* **7**, 3153–3172 (2014).
73. Vignati, E., Wilson, J. & Stier, P. M7: An efficient size-resolved aerosol microphysics module for large-scale aerosol transport models. *J. Geophys. Res.: Atmospheres* **109**, (2004).
74. Pozzer, A. *et al.* Simulation of organics in the atmosphere: evaluation of EMACv2.54 with the Mainz Organic Mechanism (MOM) coupled to the ORACLE (v1.0) submodel. *Geosci. Model Dev.* **15**, 2673–2710 (2022).
75. Voigtländer, J., Duplissy, J., Rondo, L., Kürten, A. & Stratmann, F. Numerical simulations of mixing conditions and aerosol dynamics in the CERN CLOUD chamber. *Atmos. Chem. Phys.* **12**, 2205–2214 (2012).
76. He, X.-C. *et al.* Iodine oxoacids enhance nucleation of sulfuric acid particles in the atmosphere. *Science* **382**, 1308–1314 (2023).
77. He, X.-C. *et al.* Determination of the collision rate coefficient between charged iodic acid clusters and iodic acid using the appearance time method. *Aerosol Sci. Tech.* **55**, 231–242 (2021).
78. Lehtipalo, K. *et al.* The effect of acid–base clustering and ions on the growth of atmospheric nano-particles. *Nat. Commun.* **7**, 11594 (2016).
79. Henschel, H., Kurtén, T. & Vehkamäki, H. Computational Study on the Effect of Hydration on New Particle Formation in the Sulfuric Acid/Ammonia and Sulfuric Acid/Dimethylamine Systems. *J. Phys. Chem. A* **120**, 1886–1896 (2016).
80. Kecorius, S. *et al.* Rapid growth of Aitken-mode particles during Arctic summer by fog chemical processing and its implication. *PNAS Nexus* **2**, pgad124 (2023).
81. Sipilä, M. *et al.* Molecular-scale evidence of aerosol particle formation via sequential addition of HIO3. *Nature* **537**, 532–534 (2016).
82. Davis, D. *et al.* DMS oxidation in the Antarctic marine boundary layer: Comparison of model simulations and held observations of DMS, DMSO, $DMSO_2$, H_2SO_4 (g), MSA(g), and MSA(p). *J. Geophys. Res. Atmos.* **103**, 1657–1678 (1998).
83. Zheng, G. *et al.* New particle formation in the remote marine boundary layer. *Nat Commun* **12**, 527 (2021).
84. Mauldin, R. L., Tanner, D. J., Heath, J. A., Huebert, B. J. & Eisele, F. L. Observations of H_2SO_4 and MSA during PEM-Tropics-A. *J. Geophys. Res. Atmos.* **104**, 5801–5816 (1999).
85. Zhang, Y. *et al.* Surface and free tropospheric sources of methanesulfonic acid over the tropical Pacific Ocean. *Geophys. Res. Lett.* **41**, 5239–5245 (2014).
86. Miljevic, B. *et al.* Aerosol Acidity Controls Methanesulfonic Acid Evaporation From Aerosols During Antarctic Katabatic Outflow. *Nat. Commun. Earth & Environ., accepted*

manuscript https://doi.org/10.21203/rs.3.rs-6905825/v1 (2025) doi:10.21203/rs.3.rs-6905825/v1.

87. Joge, S. D. *et al.* Climate warming increases global oceanic dimethyl sulfide emissions. *Proc. Natl. Acad. Sci. U.S.A.* **122**, e2502077122 (2025).

88. Wentworth, G. R. *et al.* Ammonia in the summertime Arctic marine boundary layer: sources, sinks, and implications. *Atmos. Chem. Phys.* **16**, 1937–1953 (2016).

89. Boyer, M. *et al.* Penguin guano is an important source of climate-relevant aerosol particles in Antarctica. *Commun Earth Environ* **6**, 368 (2025).

90. Fisher, J. A. *et al.* Sources, distribution, and acidity of sulfate–ammonium aerosol in the Arctic in winter–spring. *Atmos. Environ.* **45**, 7301–7318 (2011).

91. Lutsch, E., Dammers, E., Conway, S. & Strong, K. Long-range transport of NH3, CO, HCN, and C2H6 from the 2014 Canadian Wildfires. *Geophys. Res. Lett.* **43**, 8286–8297 (2016).

92. Bian, H. *et al.* Observationally constrained analysis of sulfur cycle in the marine atmosphere with NASA ATom measurements and AeroCom model simulations. *Atmos. Chem. Phys.* **24**, 1717–1741 (2024).

93. Tashmim, L., Porter, W. C., Bertram, T. H., Kilgour, D. B. & Rollins, A. Global Impacts of Marine Methanethiol Emissions and Chemistry in the Atmosphere. *Environ. Sci. Technol.* **59**, 20421–20428 (2025).

94. Jongebloed, U. A. *et al.* Underestimated Passive Volcanic Sulfur Degassing Implies Overestimated Anthropogenic Aerosol Forcing. *Geophys. Res. Lett.* **50**, e2022GL102061 (2023).

Nature manuscript 2025-07-18931

Title: "Role of methanesulfonic acid in atmospheric particle formation"

We thank the reviewers for evaluating our revised manuscript and responses to the first round of review. We also thank Reviewers 4 and 5 for their additional assessment and constructive suggestions, which helped to improve the manuscript.

Below, we provide point-by-point responses to all reviewer comments. Reviewer comments are shown in **black**, our responses in **green**, the original manuscript text in **orange**, and the revised text in **blue**.

Data correction

During the revision of the manuscript, we identified that two data points at −10 °C in the Fig. 4 growth rate plot required correction. The MSA concentration for these two points from the CLOUD16 campaign needed to be adjusted by roughly a factor of two due to a data analysis error in peak area assignment. This has now been corrected, as presented in Fig. R1 and the revised main text. All other data remain unaffected, as they were derived from different CLOUD campaigns with correct procedures.

These two data points were introduced to support the conclusion that MSA growth occurs at −10 °C under low base-to-acid ratio conditions (<< 1), complementing the existing 10 °C data points (red squares), which show the same behaviour. The key message - *that under low base-to-acid ratio conditions, MSA condenses efficiently at close to the kinetic limit at or below 10 °C* - remains unchanged. However, since the corrected data now lie slightly below the dashed line, likely due to inter-campaign systematic uncertainties, we have made minor adjustments to the main text to ensure our conclusions remain conservative for −10 °C conditions.

Original (lines 254 – 258):
However, once MSA is included in the total acid concentration, the measurements either align with or slightly exceed the kinetic limit (Figs. 4c and d), indicating that MSA drives particle growth at the kinetic limit below +9 °C. At −10 °C and below, we measure growth rates exceeding the kinetic limit for condensation of acid monomers.

Revised (lines 242 – 246):
However, once MSA is included in the total acid concentration, the measurements either align with or approach the kinetic limit (Figs. 4c and d), indicating that MSA drives particle growth above 1.8 nm size at or near the kinetic limit, below +9 °C. Growth rates measured above the kinetic limit result from the accretion of acid-base molecular clusters at base-to-acid ratio > 1, which are unaccounted by the acid monomer concentration (*x*-axis).

Original (lines 258 – 261):
Notably, the two measurements at −10 °C that fall directly on the kinetic line in Fig. 4d correspond to experiments with base-to-acid ratios near or below unity (NH_3 < 4 pptv) and with nucleation rates below 10 $cm^{-3}s^{-1}$.

Revised (lines 246 – 248):
Two measurements (green squares) at −10 °C that fall slightly below the kinetic line in Fig. 4d correspond to experiments with base-to-acid ratios near or below unity (NH_3 < 4 pptv) and with nucleation rates below 10 $cm^{-3}s^{-1}$.

Additionally, we have added the calibration factors and uncertainties for the relevant CLOUD16 data, as shown below:

Revised (lines 656 – 661):
During CLOUD 16, two SA calibration experiments were performed before and after the experiments, yielding calibration factors of 1.25×10^{10} and 1.85×10^{10} cm^{-3} cps cps^{-1}. An independent SA calibration of the Br-MION2-CIMS yielded a calibration factor that was 74 %

of the lower value (1.25×10^{10} cm^{-3} cps cps^{-1}). Accordingly, we adopted a final SA calibration factor of 1.25×10^{10} cm^{-3} cps cps^{-1} with an estimated uncertainty of 74–148 %.

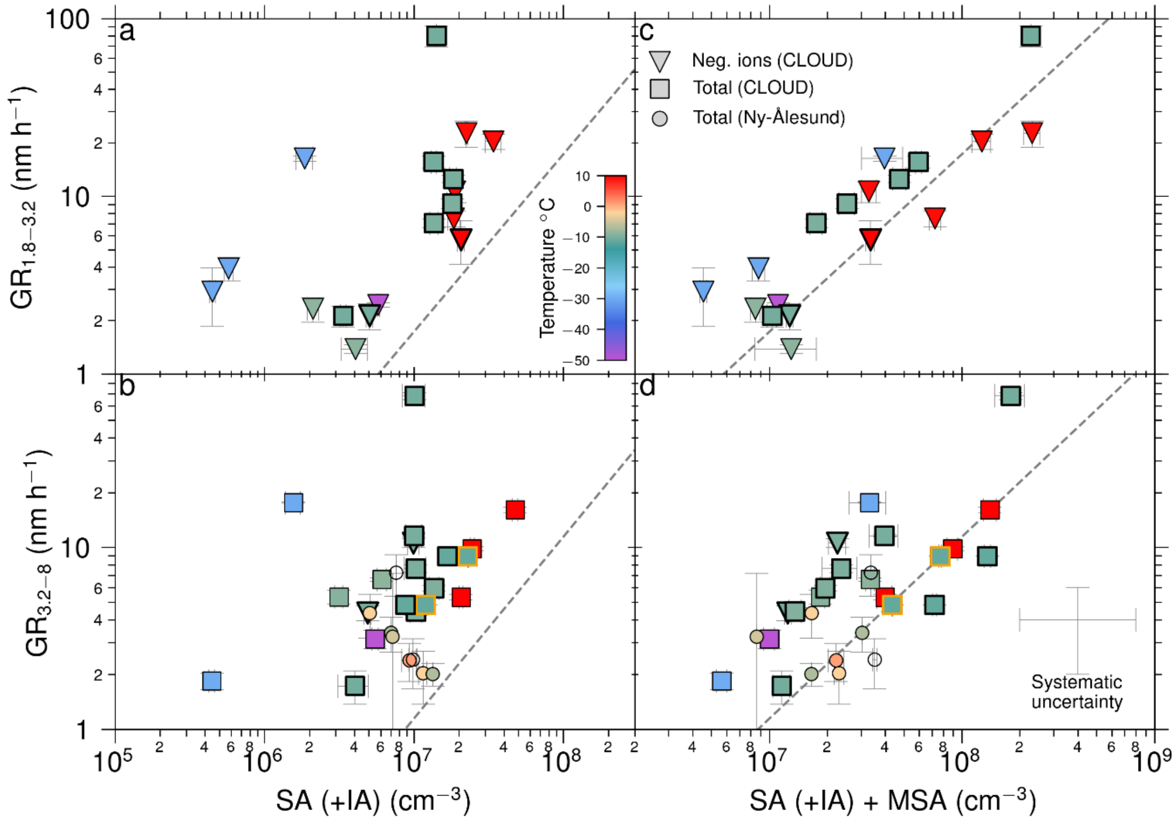

Fig. R1. Fig. 4 updated to include the uncertainty range, now indicated in the legend. Two data points at −10 °C with previously uncorrected MSA concentrations are highlighted with a thick orange outline. The corrected values points are indicated with a thick black outline, consistent with the other data points. The position of these points is unchanged in panel b, whereas in panel d they shift to the right due to correction of an error in the MSA peak area assignment.

We additionally corrected an error in the legend of Extended Data Fig. 5, where the NH_3 concentration reported for panels a–c was incorrect. These panels correspond to the same experiment partially shown in Fig. 3d–f (as can be verified from the experiment timestamps). The experiments were conducted under MSA direct injection with background base concentrations of <4 ppt NH_3 and <1 ppt DMA. The legend has been updated accordingly. This was a typo and does not affect the conclusion of this paper.

Original (lines 947 – 948):

The experimental conditions for panels a–c are 38 ppbv O_3, 6.4 ppbv SO_2, 243 pptv NH_3, and 62 % RH.

Revised (lines 970 – 971):

The experimental conditions for panels a–c are 38 ppbv O_3, 6.4 ppbv SO_2, <4 pptv NH_3, <1 pptv DMA and 62 % RH.

Referee #3 (Remarks to the Author):

I have reviewed the revised manuscript and appreciate the authors careful attention to the three reviewers' comments. It is especially noteworthy that they were able to connect the laboratory measurements of MSA nucleation to global estimates of CCN number in the revision. I support publication of the revised MS.

We thank the reviewer for evaluating the revised manuscript and for the positive assessment. We additionally appreciate the constructive comments raised in the previous round, particularly the suggestion to connect our experimental results with global simulations of CCN, which has strengthened the manuscript and broadened its impact.

Referee #4 (Remarks to the Author):

In my view, the authors have thoroughly and satisfactorily addressed nearly all of the points raised by Referee #1 in the previous review round. I commend the authors for their careful and detailed responses. I would, however, suggest clarification of the following points:

We thank the reviewer for the positive evaluation. We have addressed the points raised in the point-by-point responses below.

(1) Page 4, second paragraph: The authors conclude that MSA does not contribute to nucleation above 9 °C, partly based on the statement: "The discrepancy between the measured and predicted SA–NH_3 nucleation rates at +9 °C (Fig. 1a) is within the combined systematic uncertainties of the measurements and parametrisation." While I agree with the authors' overall conclusion based on all evidence presented in the paper, I suggest that, if feasible, the systematic uncertainties associated with both the measured and parameterized nucleation rates be included in the Fig. 1a. In addition, please clarify whether the discrepancy falls outside the combined systematic uncertainties at the other two temperatures, particularly at −10 °C.

We thank the reviewer for this suggestion. We agree that three filled square markers in Fig. 1a lie above the parameterisation line for the SA-NH_3 system, which could suggest a possible enhancement of nucleation by MSA at this temperature. However, the interpretation of whether a species enhances particle nucleation must be considered in light of the experimental and parameterisation uncertainties associated with the nucleation rate and vapour measurements. Following the reviewer's suggestion, we included systematic uncertainty estimates associated with both the measured and parameterized nucleation rates (Fig. R2). The Dunne et al. parameterisation carries an overall uncertainty of approximately a factor of 2.5 in J, while the CLOUD measurements have an estimated systematic uncertainty of ~30% in J and a factor of 2 in precursor concentrations (iodine oxoacids, SA and MSA). When these uncertainties are considered, the apparent deviation of the data points at 9 °C is not sufficiently large to distinguish them from the SA-NH_3 parameterisation. In contrast, the data at −10 °C, and especially at −30 °C, show a clear MSA-induced enhancement of nucleation that exceeds the combined uncertainties.

In addition to a comparison of the measured nucleation rates with those predicted for SA-NH_3, Fig. 1b shows a clear dependency of $J_{1.7}$ on MSA concentration at −10 °C and −30 °C, but the absence of dependency on MSA at +9 °C (where the variation in $J_{1.7}$ can be entirely explained by changes of SA, as shown in panel a).

As the reviewer recognises, a direct molecular measurement of the nucleation mechanism at each temperature is provided by the APiTOF. As discussed in detail in the section "Composition of molecular clusters", we find that MSA does not contribute to cluster nucleation at 9 °C, but participates in cluster nucleation at −10 °C and below. This observation is consistent with the conclusion derived from the nucleation rate measurements presented in Fig. 1.

We do not include systematic errors on the data points (as in Fig. R2) because they are mainly common to all measurements in a particular campaign and so represent a common scale shift

for all points rather than individual point-to-point scatter. Therefore, we have instead indicated the systematic uncertainties in the figure legend (see Fig. R3), and revised the main text to explicitly acknowledge these uncertainties.

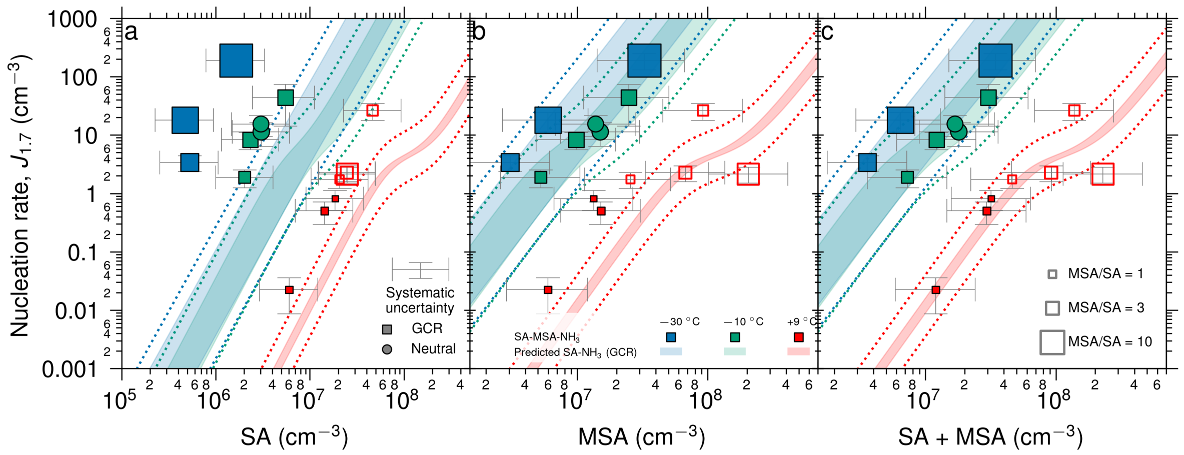

Fig. R2. Same as Fig. 1, but including systematic uncertainty estimates for the measured CLOUD nucleation rates and for the SA-NH_3 parameterization.

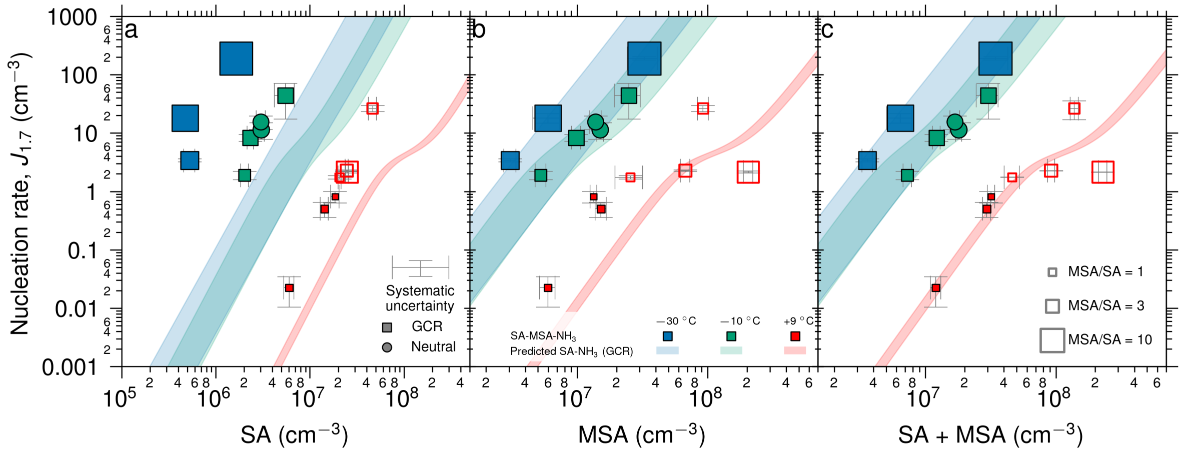

Fig. R3. Fig. 1 updated to include the systematic uncertainties in the legend od panel a.

In addition to adding the systematic uncertainties in Fig. 1, we have similarly added systematic uncertainties to the legend of Fig. 4 (growth rates versus acid concentrations).

(2) Lines 172–173: Please provide an appropriate reference for the parameterisation of the nucleation rate for the IAx–SA–NH_3 system. Alternatively, the parameterisation could be briefly described in the Methods section.

We now cite the study by He et al. (2023) [1] as the source of the parameterisation for the nucleation rate of the IA_x–SA–NH_3 system. In addition, a brief description of the parameterisation has been added to the Methods section as shown below:

<u>Revised (lines 812 – 818):</u>

The measured particle nucleation rates were compared with those expected for the SA-NH_3 system using the parameterisation of Dunne et al. [27], and with those expected for the IA_x-SA-NH_3 system based on the He et al. [40]. In the latter case, $J_{1.7}$ was estimated using the empirical relationship between the nucleation rate and the concentrations of SA, IA, and IOA. Specifically, He et al. showed that log10 [J] scales almost linearly with $\log_{10}[(HIO_3+H_2SO_4)\times HIO_2]$, such that the nucleation rate can be approximated as:

$$J = 10^{-12.54}[(HIO_3 + H_2SO_4)HIO_2]^{1.15}$$

(3) Line 712: It appears that the last term in the equation should read "−k_off" rather than "+k_off." I also suggest revising "dC/dt" in Line 711 to "dC_(t)/dt" for consistency in notation.

We thank the reviewer for identifying this typographical error. The equation has been corrected accordingly. In addition, the notation has been revised from *dC/dt* to *dC(t)/dt* for consistency following the reviewer's suggestion.

Additional Comments:

(4) Lines 172–177: I suggest that the authors calculate J1.7 using the sum of SA and MSA concentrations in the parameterisation of the IAx–SA–NH_3 system. If the calculated $J_{1.7}$ agrees with the measured values, this would indicate that MSA participates in nucleation as efficiently as SA in such IAx–SA–MSA–NH_3 systems. Such a finding would represent an important result.

We thank the reviewer for this insightful comment. To address this point, we calculated the expected $J_{1.7}$ using the parameterisation for the IA_x-SA-NH_3 system, with the sum of SA and MSA concentrations treated as the effective acid concentration (Fig. R4). They are generally consistent with the measured steady-state nucleation rates. This suggests that MSA does additionally contribute to SA-IA_x-NH_3 aerosol nucleation.

However, in this multicomponent system, it remains unclear whether such enhancement proceeds via the MSA-HIO_2 pathway or the MSA-SA-NH_3 pathway presented in this study, or some combination thereof. **TEXT REDACTED**. We therefore conclude that the apparent agreement seen in Fig. R4, panel e - when MSA is treated as equivalent to SA or iodic acid - is coincidental, arising from the additional presence of NH_3 rather than reflecting the true mechanistic role of MSA with HIO_2. Given the complexity of multicomponent MSA-SA-IA_x-NH_3 nucleation and the insufficient constraints currently available for parameterisation, we have chosen not to include the dotted line in Extended Data Fig. 1. Including it could mislead readers into applying (MSA + SA + HIO_3) × HIO_2 within the parameterisation, which would be physically unjustified. Fully constraining this multi-component nucleation system will be the subject of future work.

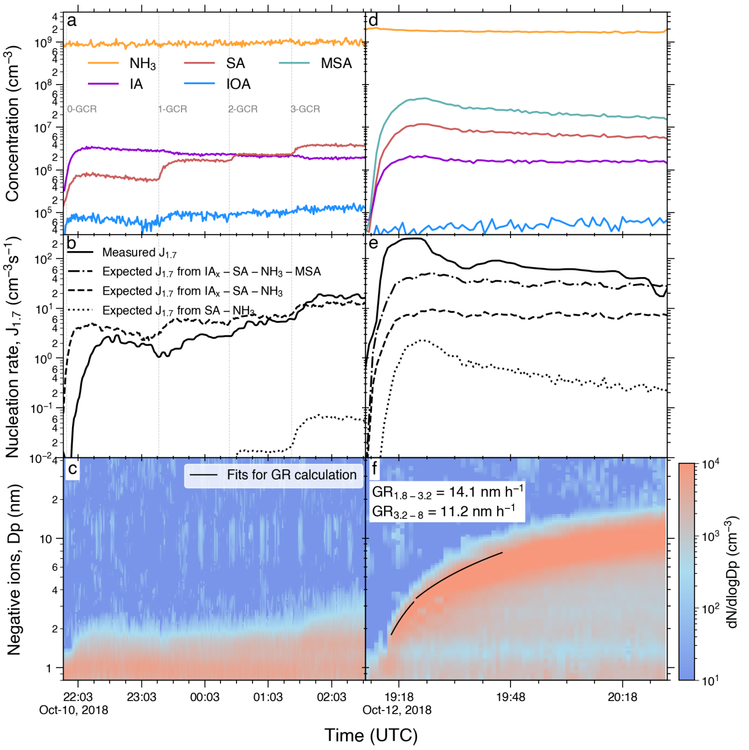

Fig. R4. Similar to Extended Data Fig. 1, but with an additional dot-dashed line in panel e. The dot-dashed line treats MSA as equivalent to SA or iodic acid, with all three acids included in the total acid concentration.

Referee #5 (Remarks to the Author):

This is an interesting paper and I have concentrated my focus on the modelling of MSA's impact on CCN with EMAC. Broadly the results are reasonable and make the impact of the new nucleation mechanism clearer. However, I have a few comments which should be attended to before the manuscript is considered for publication.

We thank the reviewer for the positive evaluation. We have addressed the points raised in the point-by-point responses below.

(1) The phrase "after incorporating our new results (see Methods for further details)" should be more specific given it is the main text. It should state concisely exactly which nucleation mechanism(s) have been added, provide the nucleation rate equation(s) and the growth rate mechanisms.

We have revised the main text to specify the nucleation mechanism implemented in the EMAC simulations. In the updated description, MSA nucleation is represented by allowing the sum of MSA and SA concentrations to enter the ternary (neutral and ion-induced) $J_{1.7}$ nucleation parameterisation of Dunne et al. (2016) [2], when thermodynamic conditions allow MSA to participate in nucleation. These conditions include temperatures below −10 °C and an ambient NH_3 concentration exceeding three times the MSA concentration, based on the E-AIM thermodynamic treatment of MSA volatility[3]. Particle growth by MSA is treated as sulfuric-acid-like condensation under cold conditions following Vignati et al. (2004), whereas at higher temperatures MSA is treated as a semi-volatile inorganic species. The revised main text now concisely states these mechanisms.

Original (lines 320 – 328):

To evaluate the potential impact of MSA on global new particle and CCN formation, we have carried out an initial study with the EMAC (ECHAM/MESSy Atmospheric Chemistry) Earth system model, which incorporates multicomponent parameterisations of atmospheric new particle formation from CLOUD experiments. For the present study, the baseline CCN concentrations consider only SA(-NH_3), calculated using the Dunne et al. parameterisation [30], together with all primary aerosol emissions. After incorporating our new results in the EMAC model (see Methods for further details), our simulations indicate that MSA enhances CCN concentrations especially strongly in the Arctic and Antarctic coastal regions, from the boundary layer up to at least 4 km altitude (Extended Data Fig. 7).

Revised (lines 307 – 314):

To evaluate the impact of MSA on global NPF, we have carried out an initial study with the EMAC (ECHAM/MESSy Atmospheric Chemistry) Earth system model (extended Data Fig. 7). Our baseline simulation considers only SA(-NH_3) nucleation [27], (labelled "without" MSA). We compare this with a simulation that adds MSA-SA-NH_3 nucleation (labelled "with" MSA). Here the sum of MSA and SA drives nucleation when the temperature is below −10 °C and [NH_3] > 3×[MSA]; outside these conditions, MSA does not contribute to nucleation. Under conditions where MSA volatility is low [15], MSA is considered to contribute equally with SA to

particle growth, whereas it is treated as semi-volatile at higher temperatures and dry conditions (see Methods for further details).

(2) The response of CCN in Extended Figure 7 is sensible and the point about the role of MSA in polar regions supported. CCN at 0.4% supersaturation looks to increase by 60-70% in large regions of the Southern Ocean at 2 km altitude which is indeed an important result.

That said the comment: "*Our findings suggest that this bias may be largely eliminated by including the contribution of MSA, which is unaccounted in current climate models*" should be more measured in my view given that we are not seeing a doubling of CCN (i.e. to resolve the 50% low bias referenced by the authors) and indeed, when considering an area-weighted perspective, most of the Southern Ocean sees increases in CCN of <60% at 4 km. It should be rephrased to the something like the following:

"*Our findings suggest that inclusion of this new nucleation mechanism, which is not considered in current climate models, may play an important role in reducing the Southern Ocean CCN bias*."

We agree with the reviewer and have revised the sentence accordingly.

Revised (lines 321 – 323):
Our findings suggest that inclusion of MSA-driven new particle formation, which is not considered in current climate models, may substantially reduce their radiation bias in the Southern Ocean.

(3) I understand this is primarily an experimental paper but, to demonstrate a clear process chain in the modelling study, the ratio (new mechanism / control) of total nucleation rate and total N50 and/or N100 should be plotted alongside the CCN.

We agree that examining intermediate quantities such as total nucleation rates and particle number concentrations (e.g., N50) helps illustrate the process chain linking nucleation to CCN, and we have now included these results in the updated Extended Data Fig. 7. The newly included nucleation rates, and especially the N50 ratio, closely track the CCN enhancement pattern. Taken together, our modelling results indicate that the largest enhancements from MSA-driven aerosol nucleation and growth occur in polar regions and extend from the boundary layer up to at least 4 km altitude.

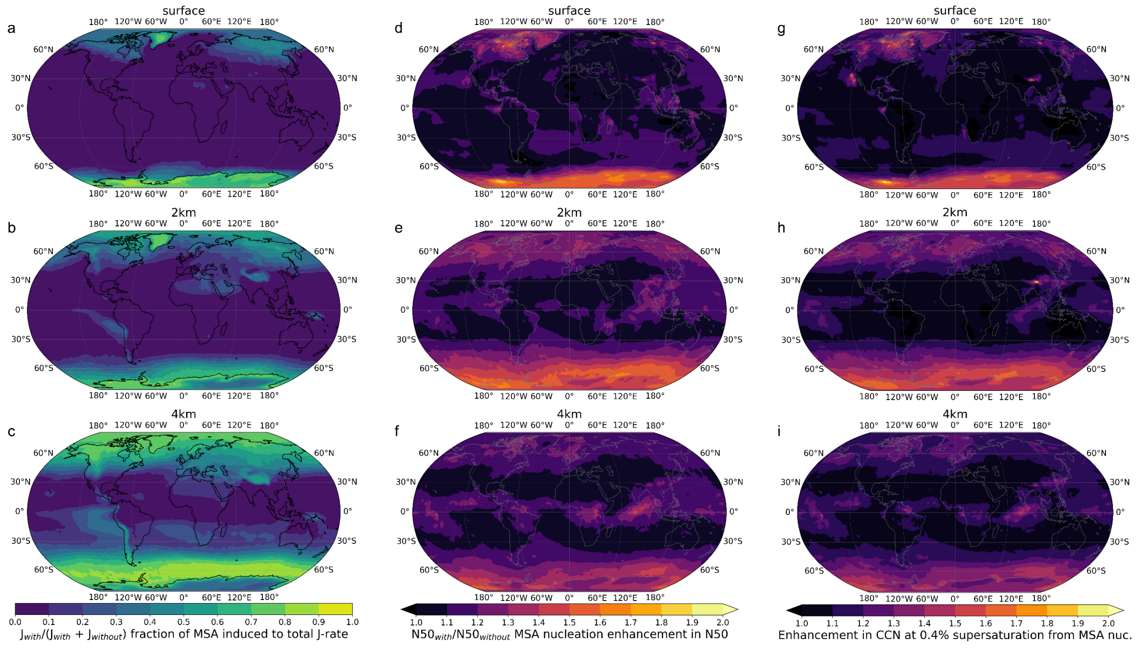

Extended Data Fig. 7. Yearly-averaged contribution of MSA to particle nucleation rates, N50 and CCN concentration simulated with the EMAC model. *Panels a–c show the relative importance of MSA by comparing nucleation rates (J) with and without MSA, expressed as [$J_{with\ MSA}$ / ($J_{with\ MSA}$+$J_{without\ MSA}$)] at a) the surface, b) 2 km altitude, and c) 4 km altitude for the period 2016–2019. Panels d–f show the corresponding yearly averaged enhancement in N50 concentration due to MSA at d) the surface, e) 2 km altitude, and f) 4 km altitude for the period 2016–2019. Panels g–i show the corresponding yearly-averaged enhancement in CCN concentration at 0.4% supersaturation due to MSA at g) the surface, h) 2 km altitude, and i) 4 km altitude, for the period 2016–2019. The CCN enhancement ratio represents CCN concentrations relative to the baseline simulation, after accounting for SA-MSA-NH_3 nucleation and MSA-driven particle growth measured in this study (see Methods for details). The baseline simulation considers only SA(-NH_3) particle formation, calculated according to the Dunne et al. parameterisation [27], together with all primary aerosol emissions [103]*
